# Designing and immuno-informatics evaluation of a multi-epitope vaccine targeting lipoprotein A-4′-phosphatase (LpxF) for *Helicobacter pylori* infection control

**DOI:** 10.3389/fbinf.2026.1779654

**Published:** 2026-03-18

**Authors:** Pavan Gollapalli, Tamizh Selvan Gnanasekaran

**Affiliations:** 1 Nitte (Deemed to be University), Nitte University Centre for Science Education and Research (NUCSER), Department of Bioinformatics and Biostatistics, Paneer Campus, Mangalore, Karnataka, India; 2 Nitte (Deemed to be University), KS Hegde Medical Academy (KSHEMA), Centre for Bioinformatics, Mangalore, Karnataka, India; 3 Nitte (Deemed to be University), KS Hegde Medical Academy (KSHEMA), Central Research Laboratory, Mangalore, Karnataka, India

**Keywords:** *Helicobacter pylori*, *in silico* epitope prediction, lipid A-4′phosphatase (LpxF), molecular dynamic simulation, multi-epitope vaccine, vaccine development

## Abstract

**Introduction:**

The WHO has classified *Helicobacter pylori* as a category 1 carcinogen and a major causative agent of gastrointestinal ulcers, gastric adenocarcinoma, and gastric lymphoma. While antibiotics and proton pump inhibitors are effective treatments, they are associated with risks of reinfection, patient dissatisfaction, and increasing antibiotic resistance. Due to the bacterium's extremophile nature, designing potent drugs remains challenging. Therefore, an effective vaccine represents the most suitable prophylactic option for mass administration.

**Methods:**

A subtractive proteomics pipeline was employed to identify appropriate antigenic proteins for the development of a multi-epitope vaccine (MEV). Lipid A-4'phosphatase (LpxF) was selected as a potential target. Various bioinformatics and immunoinformatics databases were used to predict T and B cell epitopes. A 757 amino acid MEV was then constructed by combining eight cytotoxic T cell (CTL), nineteen helper T cell (HTL), and fourteen linear B cell (LBL) epitopes using appropriate adjuvants and linkers. The vaccine's interaction with human immunological receptors (TLR2, TLR4, and TLR5) was evaluated via molecular docking and molecular dynamics (MD) simulations. Finally, the pET-28a(+) plasmid vector from Escherichia coli was used to assess expression capabilities.

**Results:**

The proposed MEV was found to be non-allergic, stable, and highly antigenic for human use. Computational simulations, including molecular docking and MD, demonstrated strong binding affinity and stable molecular interactions between the MEV and target immune receptors. In silico cloning results further confirmed the expression potential of the vaccine within the *E. coli* system.

**Discussion:**

Based on these computational findings, the designed MEV shows significant promise for establishing protective immunity against *H. pylori*. The multi-epitope approach addresses the challenges posed by the bacterium's resilient nature. However, while the in silico results are encouraging, further *in vitro* and *in vivo* investigations are required to fully comprehend and validate its immune-protective efficacy in biological systems.

## 1 Introduction

Despite decades of effort, overcoming *H. pylori* infections has been an arduous task for the scientific community. More than half of the world’s population is infected with *H. pylori*, a leading cause of duodenal and gastric ulcers and gastric cancer (Hathroubi et al., 2018). Persistent infection drives a significant inflammatory response in the gastric mucosa, characterized by chronic gastritis, which significantly contributes to global cancer-related morbidity and mortality. Targeting the bacterium has become quite challenging because of its ability to adapt to adverse environmental conditions, such as pH, temperature, adhesion and phenotypic forms (Prasad et al., 2019). The previous unsuccessful treatment attempts and growing antibiotic resistance impede the pathogen’s eradication and development of multidrug resistance (MDR) strains. The WHO has classified *H. pylori* as a category 1 carcinogen, significantly contributing to the 1.03 million new cases of gastric cancer reported annually (Boyanova et al., 2019; Rawla and Barsouk, 2019). Several factors and mechanisms contribute to MDR development by *H. pylori*, like point mutations, efflux pumps, and biofilm formation. The emergence of MDR has become a significant challenge in managing *H. pylori* infection. The alarming *H. pylori* antibiotic resistance levels have been found with a prevalence of 80% and 40% in developing and industrialized countries, respectively (Thung et al., 2016). Scientific evidence indicates that *H. pylori* infection is primarily managed through multi-drug regimens, typically involving two antibiotics and a proton pump inhibitor (Elbehiry et al., 2024; Huang et al., 2024). However, the efficacy of these standard therapies is declining due to increasing antimicrobial resistance, high reinfection rates, and patient non-compliance (Huang et al., 2024; Sukri et al., 2021). Epidemiological data reflects a significant disparity in infection and resistance patterns globally. While infection rates in wealthy, industrialized nations generally range from 15% to 25%, they reach as high as 75%–90% in underdeveloped or developing countries (Elbehiry et al., 2024). Specifically, prevalence rates of approximately 80% have been documented in the adult populations of several developing nations, such as Saudi Arabia and Iran, whereas rates in industrialized regions like the United States are notably lower, often cited between 35% and 40% (Mirzaei et al., 2013).

Recognizing this global threat, the World Health Organization (WHO) designated clarithromycin-resistant *H. pylori* as a “high-priority” pathogen in 2017 to catalyze the research and development of novel antimicrobial agents (Sukri et al., 2021; Savoldi et al., 2018). The outer membrane of Gram-negative bacteria like *H. pylori* is generally constituted with macromolecules like lipopolysaccharide (LPS), which represents one of the essential virulence factors. This LPS is composed of lipid A embedded in the outer membrane, known as endotoxin, that induces fatal reactions in the human immune system at low concentrations. The latter is a core oligosaccharide followed by O-antigen (Raetz and Whitfield, 2002). This bacterium can form chronic colonization in the human stomach by modifying its surface structure, i.e., lipid A, by removing phosphate groups from the 1- and 4′-positions of the lipid A backbone. LpxF modifies lipid A in the outer membrane LPS, enabling *H. pylori* colonization by evading host innate immunity and antimicrobial peptides, as shown by Cullen et al. (2011). While LpxF is localized in the inner membrane, it remains a viable vaccine target because internal bacterial proteins are released during natural cell turnover or immune-mediated lysis. Once released, these proteins are captured and processed by professional antigen-presenting cells (APCs) for MHC presentation (Roche and Furuta, 2015; Comber and Philip, 2014). LpxF is an essential 4′-phosphatase that modifies the lipid A head group, a modification crucial for *H. pylori* to resist cationic antimicrobial peptides (CAMPs) and evade TLR4 detection (Needham et al., 2013). Although inner membrane localized, epitopes from LpxF can be processed by antigen-presenting cells (APCs) for MHC presentation, inducing Th1-biased T-cell responses observed in immune simulations with high IFN-γ, IL-2, and memory T-cells (Cullen et al., 2011).

TLRs are essential for pathogen identification, host-pathogen interaction, as well as regulating downstream signals (Bhattacharya et al., 2020). Pattern recognition receptors that identify the pathogen’s molecular patterns associated with it are primarily responsible for the innate immune response (Nürnberger et al., 2004). Nod1, an intracellular pattern recognition receptor, is triggered by CagA strains and recognizes the PAMP. The most studied pathogen recognition receptors (PRRs) are toll-like receptors (TLRs) (Netea et al., 2004). The TLR family includes eleven proteins, each of which binds to a distinct PAMP and is produced on the surface of cells. Transfected cell line investigations reveal that *H. pylori* uses TLRs (TLR2, TLR4 and TLR5) to induce the production of pro-inflammatory genes (Torok et al., 2005; Ding et al., 2005; Ishihara et al., 2004). The pathogenic role of the predicted proteins in *H. pylori* infections makes them more appropriate for vaccine development that will provide long-term protection against *H. pylori* infections. There is currently no vaccine that effectively prevents *H. pylori* infection. Traditional vaccine candidates such as UreB and VacA have been extensively studied; however, their efficacy can be limited by strain-specific variability and the bacterium’s ability to induce localized immunosuppression (Zhang et al., 2024; Sutton and Boag, 2019). In contrast, targeting Lipid A-4′-phosphatase (LpxF) offers a novel advantage. LpxF is an essential enzyme responsible for the chemical modification of lipid A, a process that allows *H. pylori* to evade host innate immunity by resisting cationic antimicrobial peptides and avoiding TLR4 recognition (Cullen et al., 2011; Needham and Trent, 2013). By focusing on a highly conserved protein critical for immune evasion, this strategy aims to provide a more robust and universal protective response that complements existing antigen-based designs. To develop a powerful vaccine, investigation into the link between *H. pylori* and human immune responses is required (Sutton and Boag, 2019). The host immune system recognizes the antigenic regions of pathogens known as epitopes and causes humoral or cell-mediated immunity to be activated against them (Larsen et al., 2006). Antigen-presenting cells (APCs) stimulate cytotoxic T lymphocytes as soon as an infection occurs; these cells play a major part in eliminating the infected cells (Khan et al., 2018). The surface of *H. pylori*-infected cells is acted upon by peptides linked to MHC (Ali et al., 2019). MHC molecules thus exhibit a broad variety of high-binding affinity epitopes.

There is currently no licensed vaccine that effectively prevents *H. pylori* infection. Traditional vaccine candidates targeting antigens such as UreB and VacA have been extensively studied, yet their clinical efficacy is often limited by significant strain-specific variability and the bacterium’s ability to induce localized immunosuppression. In contrast, our approach focuses on Lipoprotein A-4′-phosphatase (LpxF), a highly conserved enzyme essential for the chemical modification of lipid A. By modifying its surface, the bacterium evades host innate immunity and resists cationic antimicrobial peptides. Our multi-epitope vaccine (MEV) design aims to dismantle this primary defense mechanism by combining highly conserved CTL, HTL, and B-cell epitopes, thereby providing a robust and universal protective response that circumvents the limitations of single-antigen designs. The multi-epitope vaccine targeting LpxF represents a sustainable vaccine alternative for *H*. *pylori* infection control by enabling targeted, long-lasting immunity while reducing dependence on antibiotics and minimizing antimicrobial resistance.

## 2 Materials and methods

**FIGURE 1:**
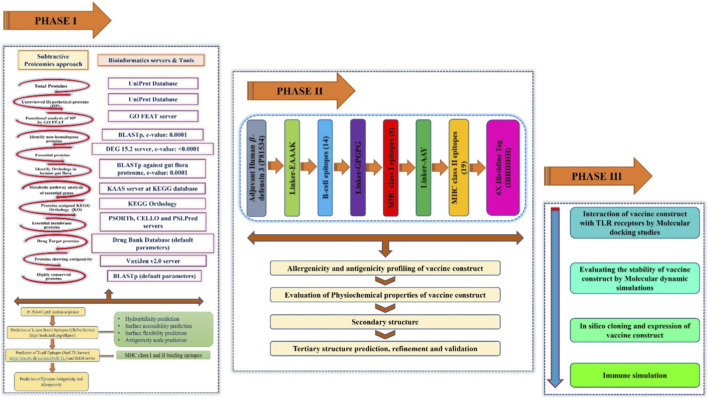
Comprehensive computational workflow for the design of a multi-epitope vaccine against *H. pylori*. The flowchart illustrates the three major phases of the study: Phase I (Subtractive Proteomics for target identification and epitope mapping), Phase II (Vaccine construction, physicochemical profiling, and structural validation), and Phase III (Molecular docking, dynamics, and immune simulation). Each step specifies the bioinformatics servers and databases utilized to ensure reproducibility.

Figure 1 illustrates the pipeline that was employed for constructing the vaccine candidate. Phase I involved using a subtractive proteomics approach to identify candidate antigenic proteins; Phase II involved the construction of a vaccine candidate, predicting its structure, and validating it; and Phase III involved docking with the immune receptor, immune simulation, and *in silico* expression.

### 2.1 Subtractive proteomics approach for identifying candidate protein for vaccine design

#### 2.1.1 Retrieval of the proteomic data set

The whole proteome (unreviewed) of *H. pylori* (strain ATCC700392/26695)/*Campylobacter pylori* was retrieved from the UniProt database (proteome ID: UP00000429). The reference proteome contains a total of 1,115 proteins and 944 unreviewed proteins.

#### 2.1.2 Functional annotation of hypothetical proteins

Functional annotation of the 944 unreviewed hypothetical proteins (HP) was performed using the GO FEAT 1.0 server (http://computationalbiology.ufpa.br/gofeat/) (e-value 1e-03) (Araujo et al., 2018), integrating data from UniProt (https://www.uniprot.org/) (Chen et al., 2017), InterPro (https://www.ebi.ac.uk/interpro/) (Finn et al., 2017), Pfam (http://pfam.xfam.org/) (El-Gebali et al., 2019), NCBI (https://www.ncbi.nlm.nih.gov/) and EMBL (https://www.ebi.ac.uk/) databases. The HPs set describing the functional domain and/or protein family were considered for further analysis.

#### 2.1.3 Identification of essential non-homologous proteins

Candidate proteins were screened for non-homology against the human proteome (BLASTp, e-value <0.0005) and the human gut microbiome (e-value < 0.0001) to ensure safety (Anishetty et al., 2005). Essentiality was determined *via* BLASTp against the Database of Essential Proteins (DEG) with a threshold e-value <0.0001 (Zhang et al., 2004).

#### 2.1.4 Identification of orthologs in the human gut microbiome

Gastrointestinal digestion in humans mainly depends upon microbes' activity in the gut region. In human gastrointestinal digestion, gut microbiota plays a critical role. The gastrointestinal tract of healthy humans houses about 1010–1014 microbes and exists in a symbiotic association with the host (Fujimura et al., 2010). Adverse pharmacokinetic side effects in the host may be developed due to the interaction and binding of the pathogenic homologous proteins with human gut microbiota proteins. Hence, the above-identified essential proteins were screened for non-homology with human gut flora using BLASTp with an e-value<0.0001 (Altschul et al., 1990; Raman et al., 2008).

#### 2.1.5 Metabolic pathway analysis

The Kyoto Encyclopedia of Genes and Genomes (KEGG) pathway database was used for the metabolic pathway comparison of *H. pylori* 26695 (hpy) and *H. sapiens* (hsa) (Paduano and Forbes, 2015). This allows for identifying the pathogen-specific unique pathways. The non-homologous essential proteins were further screened by performing BLASTp analysis using the KASS server (Moriya et al., 2007), and the only proteins involved in pathogen-specific pathways were sorted for further analysis.

#### 2.1.6 Prediction of protein subcellular localization

The five major localizations of Gram-negative bacterial proteins include cytoplasm, inner membrane, peripheral membrane, outer membrane and extracellular region (Yu et al., 2010). Subcellular localization prediction is essential for categorizing the proteins as a drug target or vaccine candidate. Generally, the proteins in the cytoplasm can be referred to as drug targets and those on the membrane as vaccine targets (Barh et al., 2011). In this study, subcellular localization of the shortlisted proteins is identified using PSORTb v3.2 server (Yu et al., 2010), CELLO v2.5 server (Yu et al., 2004) and PSLPred (Bhasin et al., 2005). The exact position prediction provided by all three servers, or at least any two servers, is used for protein localization. Only membrane proteins were considered a potential drug target and vaccine candidates and used for druggability analysis. This inner membrane protein LpxF does not show any homology with the human proteome to avoid a potential autoimmune response.

#### 2.1.7 Druggability analysis

Using the BLASTp software with an e-value of 0.001, the DrugBank 5.0 database (Wishart et al., 2018) was used to classify novel drug targets or vaccine candidates from membrane proteins with default parameters.

#### 2.1.8 “Anti-target” analysis

The adverse pharmacological effects may be developed in the human host due to the binding and interaction of the therapeutic compounds instead of acting against pathogenic proteins due to their homology. Anti-targets are analogous to human proteins, and a total of 210 proteins were identified *via* a literature search (Shanmugham and Pan, 2013). BLASTp at NCBI was used for this analysis by submitting the identified novel membrane proteins against these ‘anti-targets’ with e-value<0.005 and identity <25%.

#### 2.1.9 Antigenicity and allergenicity of druggable proteins

The vaccine construct was evaluated for allergenicity using AlgPred (threshold 0.4) (https://webs.iiitd.edu.in/raghava/algpred2/) (Saha and Raghava, 2006) and AllergenFP (https://ddg-pharmfac.net/AllergenFP/) (Dimitro et al., 2014). Antigenicity was cross-validated using Vaxijen 2.0 (https://www.ddg-pharmfac.net/vaxijen/VaxiJen/VaxiJen.html) (Doytchinova and Flower, 2007) and ANTIGENpro (https://scratch.proteomics.ics.uci.edu/) (Magnan et al., 2010). This step reveals the possible antigenic proteins from the set of druggable membrane proteins, by which further immunodominant peptides can be screened for peptide-based vaccine development. The protein showing hits >0.4 was used for conservative analysis.

#### 2.1.10 Comparison of predicted sequences with other strains

The spectrum of drugs in the entire homologous bacterial community can be determined by estimating conservation patterns among predicted sequences with different classically used strains of the same species. BLASTp at NCBI was used for this analysis by keeping all the parameters as default except “*Helicobacter pylori*” in the organism option.

#### 2.1.11 Virulence factor analysis

A comprehensive data set of virulence factors defined by sixteen dominant bacterial pathogens was provided by the Virulence Factor Database (VFDB) (Chen et al., 2004). These virulence factors cause the colonization of bacterial pathogens and are detrimental to the host cell.

### 2.2 Immunoinformatic approach for epitope-based peptide design and validation

#### 2.2.1 Antigenicity of selected LpxF

The antigenicity of the selected structural proteins was predicted using the VaxiJen v.2.0 (http://www.ddgpharmfac.net/vaxijen/) server, and a default viral threshold value of 0.4 was used (Doytchinova and Flower, 2007; Magnan et al., 2010). We chose the structural protein sequences with a high antigenic score for the next stage of the LpxF protein investigation.

#### 2.2.2 Cytotoxic T-lymphocyte epitope prediction and assessment

CTLs are among several immune system cell types that have the ability to directly engage with and eradicate infected cells (Xu et al., 2020). Because CTLs may infiltrate pathogenic cells, they are essential to the host defence mechanism. A web-based program called NetCTL1.2 (http://www.cbs.dtu.dk/services/NetCTL/) was used to predict CTL epitopes for the *H. pylori* LpxF protein (Larsen et al., 2007). The service predicts the 9-mer epitopes of the query protein based on its transport-associated protein, C-terminal cleavage scores, and MHC-I binding scores. The weight matrix establishes the Tap transport efficiency, and the server employs an artificial neural network for C-terminal cleavage and peptide binding to MHC-I. The epitopes were predicted using the default threshold of 0.75.

#### 2.2.3 Helper T-lymphocyte epitope prediction and evaluation

The recognition of foreign antigens by HTLs triggers the activation of B cells and cytotoxic T cells, which eliminate pathogenic pathogens and are crucial for inducing adaptive immunity. The CONSENSUS approach, which is based on a percentile rank of 5% (Wang et al., 2010), was used to select the HTL epitopes following the MHC class II binding allele prediction tool IEDB (http://tools.iedb.org/mhcii/) was used to identify the HTL epitopes. A set of seven human HLAs, including HLA-DRB1 * 15:01, HLA-DRB4 * 01:01, HLA-DRB3 * 01:01, HLA-DRB5 * 01:01, HLA-DRB1 * 03:01, HLA-DRB3 * 02:02, and HLA-DRB1 * 07:01, were predicted to have 15-mer epitopes for MHC-II binding of LpxF proteins. The tool calculates the affinity of a peptide for the MHC-II based on the IC50 score. IC50 values below 50 nM indicate the highest binding affinity for MHC-II, IC50 values below 500 nM indicate medium affinity, and IC50 values below 5,000 nM indicate the lowest binding affinity.

#### 2.2.4 Linear B-lymphocyte epitopes prediction

B-cell epitopes significantly influence the onset of humoral or antibody-mediated immunity. Through their interactions with secreted antibodies, B cells activate the immune system and eliminate infectious agents (Nain et al., 2020). We utilized the kernel method to predict 20-mer linear B-cell epitopes by employing a web-based application called BCPred (http://ailab.ist.psu.edu/bcpred/) (EL‐Manzalawy et al., 2008). BCPred adopts the support vector machine (SVM) method to predict B-cell (linear) epitopes.

To predict discontinuous B cell epitopes, the ElliPro web server (http://tools.iedb.org/ellipro/) was utilised. The ElliPro suite predicts B-cell (conformational) epitopes by combining Thornton’s methodology with residue clustering methods. The service uses Modeller v9.20 to create 3D coordinates of the epitopes it predicts. The tool provides a PI (protrusion index) value for each predicted epitope (Ponomarenko et al., 2008). However, only predicted linear B-cell epitopes (LBEs) were selected for inclusion in the final vaccine construct. This approach was adopted because linear epitopes maintain their immunogenic potential regardless of the final global fold of the synthetic multi-epitope protein, whereas conformational epitopes are highly dependent on the native 3D architecture of the parent protein which may not be preserved in a chimeric vaccine design.

#### 2.2.5 Multi-epitope vaccine construct, structural modelling and validation

A set of CTL and HTL epitopes were selected based on their high binding score and non-allergic properties. The final multi-epitope vaccine design was designed by using AAY linkers to link the selected CTL epitopes and GPGPG linkers to link the HTL epitopes. Linkers facilitate appropriate separation and epitope representation. For two reasons, linkers are important: (1) they enhance epitope presentation, and (2) they successfully inhibit the generation of neo-epitopes or junctional epitopes (Saadi et al., 2017; Eslami et al., 2019). Human β-defensins were also added to the vaccine at the N-terminus using an adjuvant called EAAAK linker to increase immunogenicity. The EAAAK linker was utilized to ensure the separation of domains, while GPGPG linkers were used to prevent junctional epitope formation and enhance immune processing (Arai et al., 2001).

We employed the highly accurate PSIPRED online service (http://bioinf.cs.ucl.ac.uk/psipred/) to compute the secondary structure for the vaccine sequence (Buchan and Jones, 2019). Using PSI-Blast, proteins resembling our vaccine design were identified; these sequences were then used to construct PSSM (position-specific scoring matrix). To process the PSSM and forecast the secondary structure elements, the server further employs the feed-forward neural network method.

The tertiary structure of the vaccine construct has been generated using the SWISS-Model server (https://swissmodel.expasy.org/). Discovery Studio 2023 was used to visualize the potential peptide construct for vaccine design (https://www.3ds.com/products-services/biovia/products/molecular-modeling-simulation/biovia-discovery-studio/visualization/). Utilizing QMEAN (https://swissmodel.expasy.org/qmean/), the predicted 3D structure was evaluated. The 3D structure was submitted to the online Galaxy Refine server (http://galaxy.seoklab.org/) for further refining (Ko et al., 2012). The server uses the CASP10 technique to enhance the query 3D structure. The CASP10 approach is employed to reassemble protein side chains, which are subsequently repackaged and relaxed using 3D structural simulations. Galaxyrefine was used to improve the global and structural quality of the 3D structure. The YASARA energy minimization server (https://www.yasara.org/minimization server.htm) was used to do the structural rectification and energy minimization (Krieger and Vriend, 2014). ProSA-web analysis (Wiederstein and Sippl, 2007) and the ERRAT score (Colovos and Yeates, 1993) were used to validate the tertiary structure. ProSA-web uses the expected Z-score to validate the structure. Additionally, utilizing RAMPAGE server’s Ramachandran plot analysis, the overall quality of the vaccine 3D model that was developed was assessed.

#### 2.2.6 Allergenicity and antigenicity profiling of vaccine construct

The safety profile of the final vaccine construct was evaluated by predicting its allergenicity and toxicity. Allergenicity was predicted using the AlgPred 2.0 server (https://webs.iiitd.edu.in/raghava/algpred2/) (Sharma et al., 2021), which utilizes a hybrid approach combining amino acid composition, dipeptide composition, and IgE epitope mapping to distinguish between allergens and non-allergens. This step is crucial to ensure that the vaccine remains safe for human administration and does not induce hypersensitivity. Additionally, the toxicity of the construct was analyzed using the ToxinPred server to confirm the non-toxic nature of the selected epitopes (Gupta et al., 2013).

#### 2.2.7 Evaluation of physiochemical properties of vaccine construct

Several physicochemical properties of the vaccine sequence were assessed using the publicly accessible web server ProtParam (http://www.expasy.org/protparam/) (Gasteiger et al., 2005). The molecular weight, aliphatic score, theoretical pI, *in vitro* and *in vivo* half-life, instability index, GRAVY, and amino acid content are all computed by the server.

#### 2.2.8 Solubility and disorder prediction

The solubility of the multi-epitope vaccine was evaluated using the SoluProt v1.0 server (Hon et al., 2021) to ensure its suitability for downstream expression and purification. Additionally, intrinsically disordered regions (IDRs) were predicted using the PrDOS server (Ishida and Kinoshita, 2007) with a false-positive rate of 5.0% to confirm the structural integrity of the construct while identifying flexible segments that may enhance epitope accessibility.

#### 2.2.9 Interaction analysis of vaccine with TLRs

The interaction between the vaccine and human toll-like receptors 2, 4, and 5 was evaluated by the ClusPro server (https://cluspro.org) (Kozakov et al., 2017). The server executes three computing phases: (1) rigid body docking by sampling billions of conformations; (2) clustering of the 1000 lowest energy structures produced by root-mean-square deviation (RMSD) based on identifying the largest clusters that will represent the most likely models of the complex; and (3) refinement of selected structures based on energy minimization. The docking algorithm utilized in the rigid body docking step, known as PIPER, is based on the Fast Fourier Transform (FFT) correlation approach (Kozakov et al., 2006). To quantify the binding strength, the binding affinity ( Δ G) and dissociation constant (K_d_) of the vaccine-TLR complexes were predicted using the PRODIGY (PROtein binDIng enerGY) web server (Vangone and Bonvin, 2015). Furthermore, the molecular interactions at the binding interface, including hydrogen bonds, salt bridges, and non-bonded contacts, were analyzed using PDBsum (https://www.ebi.ac.uk/thornton-srv/databases/cgi-bin/pdbsum/GetPage.pl) (Laskowski et al., 2018) and visualized *via* PyMOL v2.5 to determine the residues contributing to the stability of the complex.

#### 2.2.10 Molecular dynamics simulation

Molecular Dynamics Simulation (MDS) and energy minimization were carried out using the Linux-based application GROMACS (Groningen MAchine for Chemical Simulations) (Abraham et al., 2015). The behaviour of the vaccine structure in an *in vivo* biological system was investigated using MDS. The OPLS-AA (Optimised Potential for Liquid Simulation-All Atom) force field restrictions were used to generate the topology file required for energy minimization and equilibration. The solvent utilised to simulate the vaccine was spc216, an equilibrated three-point water model with periodic boundary conditions. Once the net charge of the vaccine construct was determined, charged ions were added to neutralize the system. A 100 ns simulation run was performed for the energy-minimized structure to find the Root Mean Square Deviation (RMSD) of the backbone and the Root Mean Square Fluctuation (RMSF) of the side chain.

#### 2.2.11 Reverse translation, codon optimization and *in silico* cloning of the vaccine

The Java Codon Adaptation Tool (JCat) (https://www.jcat.de/) was used to optimize codons and reverse translate the vaccine’s cDNA sequence, which can be efficiently expressed in *E. coli* K-12 strain129. The outcome includes GC content and a codon adaptation index (CAI) score, both of which help figure out the levels of protein expression. Furthermore, the optimized MEV was added to the pET-28a (+) vector using the SnapGene program.

#### 2.2.12 Immune simulation

To predict the real-life immunogenicity and immune profile of the vaccine, an *in silico* immune simulation was performed using the C-ImmSim server (Rapin et al., 2011). The simulation was configured using the following parameters: three injections were administered at 4-week intervals, corresponding to time steps 1, 84, and 168 (each step = 8 h). The total simulation time was set to 1,050 steps to monitor the sustained immune response over approximately 1 year. The host HLA alleles were set to match the most frequent global distribution, and all other parameters, including the random seed and simulation volume, were kept at default settings to ensure reproducibility.

#### 2.2.13 Population coverage analysis

To determine the global applicability of the designed vaccine, a population coverage analysis was performed using the IEDB population coverage tool (Bui et al., 2006). This analysis calculated the percentage of individuals in different geographical regions expected to respond to the selected T-cell epitopes based on their specific HLA allele distributions across the IEDB database (Vita et al., 2019).

## 3 Results

### 3.1 Candidate protein for vaccine design

#### 3.1.1 Analysis of the *H. pylori* HPs

A preliminary prediction for the functional annotation was carried out using the GO FEAT platform. From the total *H. pylori* 26695 reference proteome (1, 115 proteins), 944 unreviewed HPs were used for the analysis. Further, the proteins of the known domain and/or families and their GO terms were selected (542 proteins) for analysis (Supplementary Table S1). These functionally annotated proteins may play an important role in the cell and are thus labelled as HPs. The functional annotation of HPs assists in gaining knowledge of structure, function and pathways participating in the pathogenesis of bacterium and thus aid in identifying novel therapeutic targets. The human homologous proteins in the pathogen and proteins in metabolic pathways associated with the pathogen and host in common were determined using various web-based bioinformatics resources. In the present study, we used the entire set of uncharacterized HPs to analyze and predict the drug candidate protein. The primary step employed is to find the functional domains/families of the HPs. The function depends on the domains, which are the structural, functional, and evolutionary protein units. Understanding the function of the protein domain is essential to explore its role cellular level.

#### 3.1.2 Selection of non-homologous human proteins

All the HP sequences analyzed for functional domain/family were then screened only for the non-homologous sequences. BLASTp screening against the human proteome yielded 412 non-homologous proteins (Supplementary Table S2). Striking homology exists between bacteria and human proteins since these proteins are involved in a typical cellular system. Hence, the cross-reactivity with host homologous proteins must be avoided during therapeutic development and administration to bind pathogen-specific target proteins.

#### 3.1.3 Essential protein analysis

A total of 77 essential genes were identified by performing BLASTp against the DEG database (e-value<0.0001). These proteins of *H. pylori* are considered crucial for pathogen survival and are unique for that organism (Supplementary Table S3). Thus, these proteins are believed to aid as species-specific drug targets/vaccine candidates (Judson and Mekalanos, 2000). The critical genes were defined from the non-homologous set collected since essential genes are required for the bacterial proteome’s cellular processes to continue functioning (Zhang and Ren, 2015).

#### 3.1.4 Human gut microbiota analysis

The inadvertent blockage of the gut floral proteins due to homologous proteins of the pathogen may lead to hostile effects. To avoid this, homologous gut microbial proteins with essential proteins of *H. pylori* were omitted for further analysis. This step is accomplished using BLASTp by choosing the search set against human gut metagenome 16S ribosomal RNA with an expected threshold of 0.05. Here, no significant matches are found, suggesting that the entire protein set of essential proteins is unique for the pathogen. Further, gut microbiota refers to the large population of bacteria that colonize the human intestinal tract (Jandhyala et al., 2015). Pathogens that cause human inflammatory diseases are closely associated with gut microbiota; these pathogens co-evolve and self-multiply in a symbiotic relationship with gut microbiota (Pickard et al., 2017).

#### 3.1.5 Analysis of metabolic pathways

The KEGG database retrieved 342 human metabolic pathways and 95 *H. pylori*-specific metabolic pathways. In comparison, the pathways explicitly present in *H. pylori* were 25, which are unique and hence termed pathogen-specific pathways (Supplementary Table S4). For screening novel therapeutic targets, the proteins exclusively involved in the pathways specific to the pathogen were considered. In our study, 77 essential proteins were subjected to the KAAS server for assigning KEGG ontology (KO) and specific metabolic pathways. A total of 55 proteins were assigned with KO (Supplementary Table S5) and 42 proteins were involved in pathways common to *H. sapiens*. These 42 proteins were omitted for further screening to circumvent cross-reactivity with other human pathogens. Finally, we arrived at 12 unique proteins and found them involved in pathogen-specific pathways (Table 1).

**TABLE 1:** Proteins involved only in *H. pylori* specific unique pathways.

Sl. no	KO assignment	Protein name	Gene symbol	Pathway
01	K00172	Pyruvate ferredoxin oxidoreductase gamma subunit	porC	Biosynthesis of secondary metabolites; Microbial metabolism in diverse environments; Methane metabolism
02	K00175	2-oxoglutarate/2-oxoacid ferredoxin oxidoreductase subunit beta	korB	Biosynthesis of secondary metabolites; Microbial metabolism in diverse environments
03	K00177	2-oxoglutarate ferredoxin oxidoreductase subunit gamma	korC	Biosynthesis of secondary metabolites; Microbial metabolism in diverse environments
04	K01808	Ribose 5-phosphate isomerase B	rpiB	Biosynthesis of secondary metabolites; Microbial metabolism in diverse environments
05	K11753	Riboflavin kinase/FMN adenylyltransferase	ribF	Biosynthesis of secondary metabolites
06	K11784	Cyclic dehypoxanthinylfutalosine synthase	mqnC	Biosynthesis of secondary metabolites
07	K02527	3-deoxy-D-manno-octulosonic-acid transferase	kdtA	Lipopolysaccharide biosynthesis
08	K23159	Lipid A 4′-phosphatase	lpxF	Lipopolysaccharide biosynthesis
09	K05366	Penicillin-binding protein 1A	mrcA	Peptidoglycan biosynthesis; beta-Lactam resistance
10	K03203	Type IV secretion system protein VirB8	virB8	Bacterial secretion system
11	K00407	Cytochrome c oxidase cbb3-type subunit IV	ccoQ	Two-component system
12	K12368	Dipeptide transport system substrate-binding protein	dppA	Bacterial chemotaxis

#### 3.1.6 Analysis of subcellular location

The prediction of protein localization serves as a vital parameter in identifying therapeutic targets because many pathogens can span multiple locations (Golkar et al., 2014). Subcellular localization analysis identified two inner membrane, seven cytoplasmic, and three periplasmic proteins (Table 2). The inner membrane protein LpxF was selected as the primary vaccine candidate due to its conservation and role in immune evasion. The choice of LpxF is further justified by its high conservation across strains and its essential role in modifying lipid A to evade host innate immunity. Targeting an essential inner membrane protein ensures that the vaccine remains effective even under selective pressure, as the pathogen cannot easily mutate such a critical survival factor. While cytoplasmic proteins may be potential drug targets, we will focus on membrane proteins for this study because membrane proteins account for more than 60% of therapeutic targets (Michael et al., 2014). Considering membrane proteins as targets for a variety of reasons that include: (1) protein functions will be calculated using machine learning and computer-based methods before *in vivo* or *in vitro* laboratory trials; (2) A nature of the unique structure of membrane proteins will facilitate to predict and generate their secondary structure (Bartlett et al., 2013). The mutational change and exchange of genes among pathogens occur due to the overuse of broad-spectrum therapeutics. The emergence of the antibiotic resistance crisis is mainly because of the misuse of antibiotics and the lack of new therapeutics.

**TABLE 2:** The subcellular location of 12 non-homologous, essential proteins is only involved in the unique metabolic pathways of the pathogen.

Sl. no	Protein	PSORTb	CELLO v.2.5	PSLpred
1	tr|O30648	Cytoplasm	Cytoplasm	Cytoplasm
2	tr|O25312	Cytoplasm	Cytoplasm	Cytoplasm
3	tr|D7FDT9	Cytoplasm	Cytoplasm	Cytoplasm
4	tr|AOA024C7N3	Cytoplasm	Cytoplasm	Cytoplasm
5	tr|AOA3Q9Y0Z5	Cytoplasm	Cytoplasm	Cytoplasm
6	tr|O25370	Cytoplasm	Cytoplasm	Cytoplasm
7	tr|AOA7G1HQN3	Cytoplasm	Cytoplasm	Cytoplasm
8	tr|Q9ZJ31	Cytoplasmic membrane	Inner membrane	Inner membrane
9	tr|AOA3Q9Y2E2	Cytoplasmic membrane	Outer membrane	Inner membrane
10	tr|AOA086RT63	Cytoplasm	Outer membrane	Extracellular protein
11	tr|O24953	Unknown	Cytoplasm	Periplasmic membrane
12	tr|AOA1Q2RQ85	Periplasma	Periplasma	Periplasmic membrane

#### 3.1.7 Analyzing the druggability of HPs

The novelty of the membrane proteins as a drug target was analyzed using the Drug Bank database (Supplementary Table S6). The proteins with significant similarity in the database were excluded and our study, the penicillin-binding protein 1A, exhibited similarity above the threshold under consideration. However, the second protein, LpxF, reported no similarity with any current drug targets in the database.

#### 3.1.8 “Anti-target” analysis of the novel drug target

Due to the carcinogenic side effects, various drug candidates were withdrawn from the market. The cross-reactivity and carcinogenesis check is crucial in selecting an effective drug molecule (Fenner, 1989; Bakke et al., 1984). The toxicity caused by the inadvertent binding of drugs to host ‘anti-targets’ instead of pathogenic targets must be avoided. In this concern, this step resulted in no similarity with any of the human ‘anti-target’ proteins, and thus, LpxF is considered the host ‘non-anti-target’ protein.

#### 3.1.9 Antigenicity and allergenicity prediction of LpxF

The reverse vaccination method is considered one of the most powerful approaches in designing a candidate vaccine (Galan et al., 2005; Rappuoli, 2000). The small antigenic protein sequences were considered for developing a safe recombinant vaccine with the potency to fight against infectious diseases (Rappuoli, 2000). The identified novel drug target of *H. pylori*, LpxF, is subjected to the VaxiJen v2.0 server. The results reported that the LpxF was the probable antigen protein of *H. pylori* with a score of 0.5232 (threshold:0.4). Further, this membrane protein can be used to detect the high immunodominant peptide to develop an efficient subunit vaccine against *H. pylori*. Moreover, the absence of cross-reactivity with other pathogenic antigens and suitable antigenic and adhesion properties are essential for the pathogenesis of the microbe and protection against infectious disease.

#### 3.1.10 Conservation analysis of *H. pylori* 26695 LpxF sequence with other strains

A very high conservation pattern of LpxF was found among various strains of *H. pylori*. This wide range of conservatives found for the predicted LpxF ensures it is a potential drug and/or vaccine target against *H. pylori* (Oyelade and Ezugwu, 2020). The results of protein-protein BLAST showing LpxF conservation from all *H. pylori* strains from diverse geographical locations were tabulated in Supplementary Table S7. Hence, LpxF protein might be a potential vaccine candidate/therapeutic target against *H. pylori*.

#### 3.1.11 Virulence factors of pathogenic *H. pylori*

The virulence mechanism of the non-homologous, essential protein can be explored by submitting it to the virulence factor database. For the query protein LpxF, the virulence factors retrieved were listed in Supplementary Table S8.

### 3.2 Multi-epitope vaccine design

The protein sequence of Lipoprotein A-4′-phosphatase (LpxF) was from the UniProt database using the Accession Number O25261 (strain ATCC 700392/26695). This sequence served as the template for all subsequent epitope predictions and vaccine construction.

#### 3.2.1 Screening of MHC-1 (CTL) and MHC-2 (HTL) binding epitopes

NetCTL 1.2 was used to predict 190 CTL epitopes for the LpxF protein. Only eight of the 190 total epitopes were selected using the predefined criteria for MHC binding score and non-allergenicity (Table 3). The HTL epitopes were predicted for seven HLAs: HLA-DRB1 * 03:01, HLA-DRB4 * 01:01, HLA-DRB1 * 07:01, HLA-DRB3 * 02:02, HLA-DRB1 * 15:01, HLA-DRB3 * 01:01, and HLA-DRB5 * 01:01. HTL epitopes are shown in Table 4. Nineteen distinct HTL epitopes from the LpxF protein were predicted by the IEDB server.

**TABLE 3:** MHC-1I binding epitopes (HTL) peptides.

Sl. no	Allele	Start	End	Peptide	Score	Rank
1	HLA-DRB1*03:01	177	191	YPEISSDFKGSSRYG	0.9261	0.23
2	HLA-DRB1*03:01	176	190	LYPEISSDFKGSSRY	0.9069	0.33
3	HLA-DRB1*03:01	175	189	MLYPEISSDFKGSSR	0.8532	0.55
4	HLA-DRB1*03:01	178	192	PEISSDFKGSSRYGV	0.8494	0.57
5	HLA-DRB1*07:01	39	53	FLPIFVGTVSLAMRD	0.8209	0.42
6	HLA-DRB1*07:01	40	54	LPIFVGTVSLAMRDY	0.7981	0.48
7	HLA-DRB1*03:01	131	145	ALAILTDTSRVVAGQ	0.7785	0.93
8	HLA-DRB3*01:01	131	145	ALAILTDTSRVVAGQ	0.7315	0.32
9	HLA-DRB1*07:01	41	55	PIFVGTVSLAMRDYR	0.7133	0.93
10	HLA-DRB1*03:01	130	144	IALAILTDTSRVVAG	0.7013	1.4
11	HLA-DRB3*01:01	130	144	IALAILTDTSRVVAG	0.6731	0.46
12	HLA-DRB4*01:01	15	29	VYPLKSEPINEGAYI	0.6364	0.4
13	HLA-DRB4*01:01	14	28	WVYPLKSEPINEGAY	0.6251	0.43
14	HLA-DRB1*07:01	38	52	RFLPIFVGTVSLAMR	0.6191	1.6
15	HLA-DRB4*01:01	62	76	VGTLVTQGVIYGLKG	0.607	0.43
16	HLA-DRB1*03:01	132	146	LAILTDTSRVVAGQH	0.5448	2.3
17	HLA-DRB3*01:01	129	143	VIALAILTDTSRVVA	0.5267	0.83
18	HLA-DRB1*03:01	129	143	VIALAILTDTSRVVA	0.5205	2.5
19	HLA-DRB1*15:01	12	26	LLWVYPLKSEPINEG	0.5166	2

**TABLE 4:** MHC-I binding epitopes (CTL) peptides.

Sl. no	Peptide sequence	Prediction score
1	FLSLLLWVY	2.4309
2	TVSLAMRDY	1.315
3	TLVTQGVIY	0.9996
4	FSAAGFVYY	2.8343
5	AAGFVYYRY	0.9594
6	SLIAWGFAY	1.1712
7	FAYLFTSRY	1.2459
8	YKPKRWMLY	0.9297

#### 3.2.2 B-cell epitope and antibody epitope prediction

BCPred identified 10 (linear) B-cell epitopes with scores ≥0.90, each with a different length of amino acids. Three discontinuous B-cell epitopes were also predicted using the ElliPro suite. We used the server’s default settings to forecast these epitopes. The anticipated linear and conformational epitopes are included in Tables 5, 6. For the final vaccine construct, only top-ranked Linear B-cell Epitopes (LBEs) were selected based on their high VaxiJen antigenicity scores (>0.5) and non-toxic/non-allergic properties. These LBEs are critical for inducing a humoral immune response, as they allow to produce antibodies that can recognize the primary sequence of the LpxF protein during an active *H. pylori* infection.

**TABLE 5:** B-cell Epitopes prediction using BCPred. The predicted B-cell epitopes are shown in blue colour and underlined.

Sequence	MKKLKGLFLSLLLWVYPLKSEPINEGAYILEEIGDVLRFLPIFVGTVSLAMRDYRGLGELAVGTLVTQGVIYGLKGAF STAHKDGARVGFAKRPCCNSWRGMPSGHAGGAFSAAGFVYYRYGWKPALPVIALAILTDTSRVVAGQHTILQVT IGSLIAWGFAYLFTSRYKPKRWMLYPEISSDFKGSSRYGVGFSYQW
Hydrophilicity	MKKLKGLFLSLLLWVYPLKSEPINEGAYILEEIGDVLRFLPIFVGTVSLAMRDYRGLGELAVGTLVTQGVIYGLKGAFSTAHKDGARVGFAKRPCCNSW RGMPSGHAGGAFSAAGFVYYRYGWKPALPVIALAILTDTSRVVAGQHTILQVTIGSLIAWGFAYLFTSRYKPKRWMLYPEISSDFKGSSRYGVGFSYQW
Flexibility	MKKLKGLFLSLLLWVYPLKSEPINEGAYILEEIGDVLRFLPIFVGTVSLAMRDYRGLGELAVGTLVTQGVIYGLKGAFSTAHKDGARVGFAKRPCCNSW RGMPSGHAGGAFSAAGFVYYRYGWKPALPVIALAILTDTSRVVAGQHTILQVTIGSLIAWGFAYLFTSRYKPKRWMLYPEISSDFKGSSRYGVGFSYQW
Accessibility	MKKLKGLFLSLLLWVYPLKSEPINEGAYILEEIGDVLRFLPIFVGTVSLAMRDYRGLGELAVGTLVTQGVIYGLKGAFSTAHKDGARVGFAKRPCCNSW RGMPSGHAGGAFSAAGFVYYRYGWKPALPVIALAILTDTSRVVAGQHTILQVTIGSLIAWGFAYLFTSRYKPKRWMLYPEISSDFKGSSRYGVGFSYQW
Turns	MKKLKGLFLSLLLWVYPLKSEPINEGAYILEEIGDVLRFLPIFVGTVSLAMRDYRGLGELAVGTLVTQGVIYGLKGAFSTAHKDGARVGFAKRPCCNSW RGMPSGHAGGAFSAAGFVYYRYGWKPALPVIALAILTDTSRVVAGQHTILQVTIGSLIAWGFAYLFTSRYKPKRWMLYPEISSDFKGSSRYGVGFSYQW

**TABLE 6:** Predicted Linear Epitope(s): ElliPro-Antibody epitope prediction.

Sl. No	Peptide	Start	End	Number of residues	Score
1	YYRYGWKPALP	118	128	11	0.727
2	LLLWVYPLKSEPINEGAYI	11	29	19	0.721
3	LPIFVGTVSLAMRDYRGLGE	40	59	20	0.688
4	GLKGAFSTAHKDGARVGFAKRPCCNSWRGMP	73	103	31	0.637

#### 3.2.3 Vaccine construct

**FIGURE 2:**
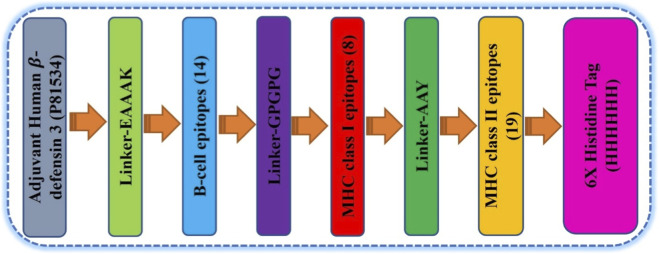
Schematic diagram of final multi-epitope peptide vaccine construct.

The adjuvant and the B-cell epitopes are linked using the EAAAK linker. The GPGPG linker was utilized to connect the CTL and B-cell epitopes. Using AAY linkers, 19 HTL and 8 CTL epitopes were combined to develop the final multi-epitope vaccination based on their binding scores, antigenic nature, and non-allergic characteristics. The strategic selection of GPGPG and AAY linkers ensures effective separation of epitopes to prevent the formation of neo-epitopes while facilitating independent folding of individual domains. This design minimizes potential misfolding risks typically associated with large recombinant proteins of this size (∼83.8 kDa). The adjuvant was attached to the vaccine’s N-terminus to prevent deterioration. The C-terminal was linked with a 6X-histidine tag was added to carry out the expression and binding of histidine protein. To increase the immunogenicity of the multi-epitope vaccine, Human β-defensin 3 (HBD3) was incorporated as a built-in adjuvant at the N-terminal end. The sequence for HBD3 was retrieved from the UniProt database using Accession Number P81534. Defensins serve as potent chemoattractants for immature dendritic cells and T-cells, thereby bridging the gap between innate and adaptive immunity, which is vital for a prophylactic *H. pylori* vaccine. The complete vaccine construct structure is shown in Figure 2. To prevent epitopes from being homologous with human proteins, the predicted epitopes were evaluated using BLASTp, and no significant similarity was detected.

#### 3.2.4 Assessment of the Vaccine’s physiochemical properties, allergenicity, and antigenicity

A crucial factor in the design of vaccine construct that normally stimulate humoral and cell-mediated immune responses against *H. pylori* is antigenic propensity. According to the predicted figures, our vaccine is antigenic, with individual antigenic probability scores of 0.6324 and 0.602676 predicted by the ANTIGENPro and VaxiJen v2.0 servers. While AllerTOP v.2.0 provided evidence for the vaccine’s non-allergenic character, AllergenFP v.1.0 determined that the vaccine construct as a non-allergenic sequence. Several essential properties were computed, including molecular weight, half-life, instability, and allergenicit. By predicting a score of −0.42, the server concluded that the finished vaccine construct is non-allergic. The standard threshold of −0.4 was used. Finding the isoelectric point allowed for the confirmation of the vaccine’s fundamental nature. With a molecular weight (MW) of 83.81 kDa, the server computed a PI score of 9.68. Conversely, half-lives >30 > 20 and >10 h were computed for yeast, *E. coli* (*in vivo*), and mammal reticulocytes *in vitro*. The vaccine would remain stable in the experimental context, as shown by the instability index of 22.06. It was determined that the aliphatic coefficient was 88.69 and GRAVY was −0.022. Thus, the vaccine design may move forward for further study. An instability index of 22.06 (below the 40.0 threshold) and a negative GRAVY score of −0.022 collectively indicate that the construct is highly stable and hydrophilic. These properties are critical for ensuring high solubility and reducing the likelihood of inclusion body formation during heterologous expression in *E. coli*.

#### 3.2.5 Structural stability, solubility, and global population coverage analysis

The structural and epidemiological viability of the designed multi-epitope vaccine (MEV) was further validated through solubility, disorder, and population coverage analyses. The solubility of the vaccine construct was evaluated using the SoluProt v1.0 server, which predicted a high solubility probability of 0.72 (threshold: 0.5) (Hon et al., 2021). This result is consistent with the negative GRAVY score (−0.022), indicating that the vaccine is likely to remain stable and soluble during over-expression in an *E. coli* host system, a critical factor for downstream industrial purification. Furthermore, the intrinsic disorder analysis conducted *via* the PrDOS server revealed that less than 15% of the vaccine construct consists of disordered regions (Ishida and Kinoshita, 2007). These disordered segments are primarily localized at the EAAAK and GPGPG linker sites. This localized flexibility is strategically beneficial, as it facilitates the independent folding of epitopes and ensures maximum accessibility for immune cell receptors during antigen processing.

To evaluate the global applicability of the MEV, a population coverage analysis was performed using the IEDB population coverage tool (Bui et al., 2006) based on the combined MHC-I and MHC-II epitopes. The results demonstrated a cumulative world population coverage of 94.2%. Notably, the vaccine showed exceptionally high coverage in regions with a high burden of *H. pylori* infection, including East Asia (91.5%), Africa (88.9%), and South Asia (90.2%). These findings suggest that the vaccine construct possesses the necessary HLA-binding breadth to serve as an effective prophylactic agent across diverse ethnic populations (Vita et al., 2019).

#### 3.2.6 Prediction of secondary structure elements

**FIGURE 3:**
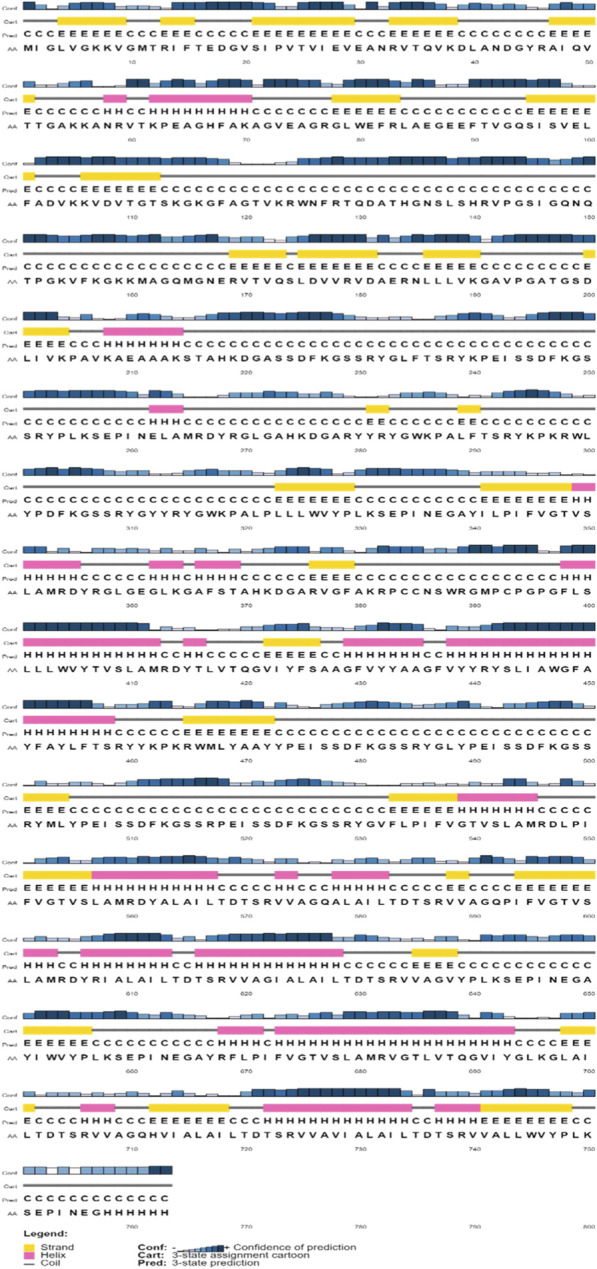
Schematic representation of secondary structure prediction of the multi-epitope vaccine; secondary structure prediction result represents the arrangement of α-helix, β-strands, and coils.

The secondary structure (Figure 3) of the vaccination protein was predicted using a web service called PSIPRED. The estimated secondary structural elements comprise 58% coils, 31% β-sheets, and 12% α-helixes.

#### 3.2.7 Three-dimensional structure modelling, refinement and validation of vaccine

**FIGURE 4:**
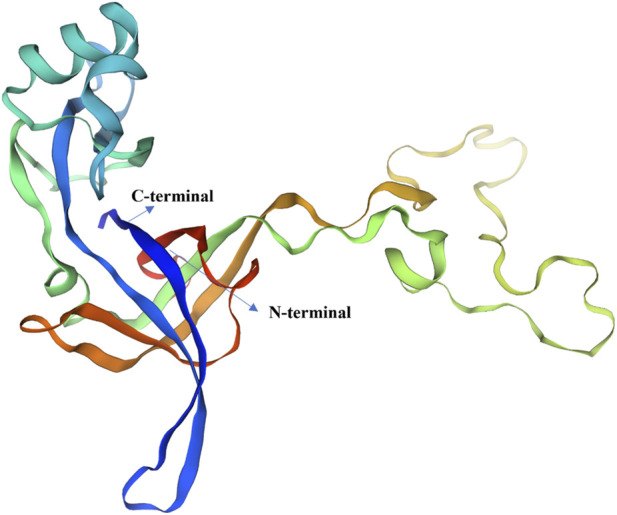
Three-dimensional (3D) structure of the final vaccine construct.

**FIGURE 5:**
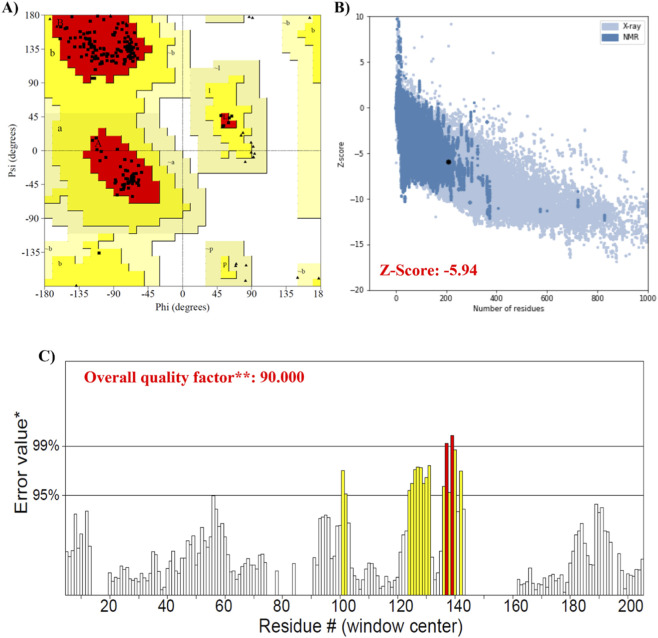
Validation of three-dimensional structure of vaccine construct. **(A)** Ramachandran plot analysis reveals that 97.1% of the amino acid residues in the vaccine structure are located in the favoured region, 2.9% of the residues fall within the allowed region, while only 0% are found in the disallowed region, indicating energetically favourable conformation, acceptable conformations, potentially unfavourable conformations, respectively. **(B)** ProSA structure validation displays **(A)** 5.94 Z score, indicating the overall quality of the construction which is within an acceptable range. **(C)** The ERRAT program evaluated the overall quality of the model, and the quality factor was 90.0. On the error axis, two lines (95% and 99%) indicate the confidence with which it is possible to reject regions that exceed that error value. Regions of the structure highlighted in grey and black can be rejected at 95% and 99% confidence level, respectively.

The SWISSMODEL was utilized to model the multi-epitope final vaccine’s three-dimensional structure. Figure 4 depicts the final structure that was acquired from this server. The selected model-1 was then refined using the Galaxy Refine tool, and its evaluation was conducted using the following metrics: RMSD (0.321), MolProbity (1.538), Ramachandran plot (99.5), GDT-HA (0.9821), Poor rotamers (0.0), and clash score (10.4). The quality of the vaccine structure was further enhanced by the YASARA energy minimization method. Using the SAVES server, the validity of the multi-epitope vaccination protein’s revised 3-dimensional modelled structure was assessed. According to ProCheck, 2.9% of vaccine residues are in the permitted zones, and 97.1% are in the preferred region. The model’s quality was further evaluated by ProSA-web, which gave a Z-score of −5.94, while the ERRAT server indicated 90% confidence (Figure 5). While the low binding energies obtained from ClusPro docking suggest a strong structural affinity between the vaccine construct and the receptors (TLR-2 and TLR-4), these values are predictive of binding stability rather than a direct measure of receptor activation or the intensity of downstream signaling pathways.

#### 3.2.8 Interaction analysis of the vaccine with TLRs

**FIGURE 6:**
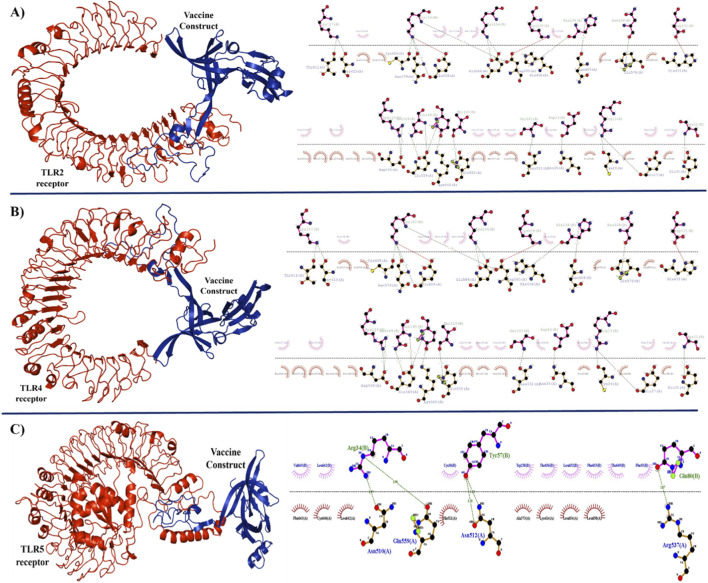
Molecular interaction analysis of the multi-epitope vaccine with human TLRs. **(A)** Vaccine-TLR2 complex, **(B)** Vaccine-TLR4 complex, and **(C)** Vaccine-TLR5 complex. The zoomed-in panels illustrate the specific amino acid residues involved in hydrogen bonding and hydrophobic interactions at the interface, indicating a stable complex formation capable of activating downstream immune signaling.

Since TLRs 2, 4 and 5 have been linked to *H. pylori* infection, TLRs are important biological receptors that help induce immunity against infections. As the development of an immune response depends on the constructed vaccine binding to these TLRs, we chose them for our investigation. The ligand binding domains of immune receptors TLR2, TLR4 and TLR5 were utilized to predict refined vaccine model interactions using ClusPro2.0, an online service for molecular docking of proteins. Multiple models were provided *via* docking studies. The cluster-generated docked model was chosen based on the largest cluster size and the lowest free energy (kcal/mol). Based on active sites, the top Cluster 0 model with a center energy of −861.1 kJ/mol and lowest energy of −877.3 kcal/mol was selected after 29 docked poses of the vaccine-TLR2 complex were evaluated. Similarly, for the Vaccine-TLR4 complex, the top cluster 4 model has a center energy of −896.4 kJ/mol and a lowest energy of −836.4 kcal/mol. For the vaccine-TLR5 complex, the top cluster 0 has a center energy of −1106.6 kJ/mol, and the lowest energy of −1213.4 kcal/mol was selected and visualized using PyMOL visualization tool (Figures 6A–C). According to interface residue analysis, all docked complexes had interaction residues within 5 Å of one another. The Protein Interaction Calculator evaluation identified the binding site’s hydrophobic and/or ionic interactions. These findings further confirmed the reliability of all the simulated docked complex models (Figure 6).

The molecular docking of the MEV with TLR4 yielded a binding affinity ( Δ G) of −12.8 kcal/mol, suggesting a highly spontaneous and stable interaction. Interaction analysis at the interface revealed the involvement of 12 hydrogen bonds and two salt bridges. Specifically, residues of the vaccine construct formed stable hydrogen bonds with residues of the TLR4 extracellular domain (Supplementary Table S9). Similar high-affinity interactions were observed for TLR2 ( Δ G = −11.5 kcal/mol) and TLR5 ( Δ G = −10.9 kcal/mol, primarily mediated by hydrophobic interactions and polar contacts at the leucine-rich repeat (LRR) regions. These results confirm that the vaccine construct can effectively bridge and activate these receptors to initiate an innate immune response (Table 7).

**TABLE 7:** Binding energy and interactions of MEV with TLR2, TLR4 and TLR5.

Complex	Binding affinity (ΔG kcal/mol)	Dissociation constant (K_d_ at 25 °C)	No. of H-bonds	No. of salt bridges
MEV - TLR4	−12.8	4.2 × 10^−10^ M	12	2
MEV - TLR2	−11.5	1.1 × 10^−9^ M	9	1
MEV - TLR5	−10.9	6.5 × 10^−9^ M	7	0

#### 3.2.9 MD simulation of TLRs-vaccine complexes

**FIGURE 7:**
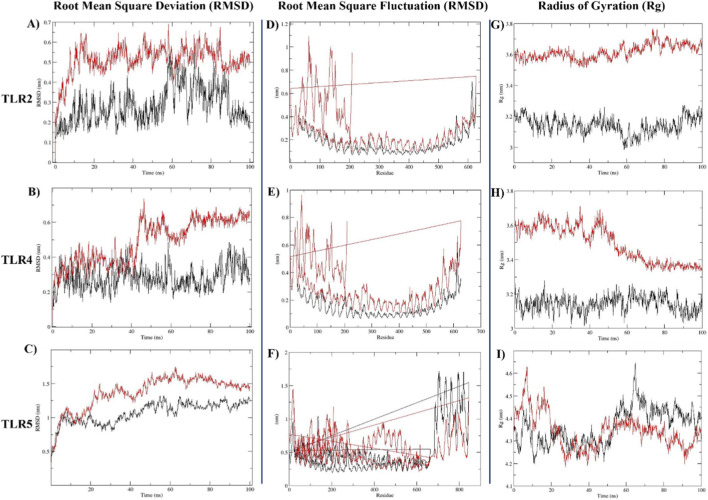
Residual stability (RMSD), fluctuations (RMSF) and compactness (R_g_) for all the systems are given. A 100 ns molecular dynamic simulations were carried out for all the TLR receptors (Apo form-black colour) and TLRs in complex with vaccine construct (Red colour). **(a,d,g)**: These plots illustrate the RMSD of the protein backbone atoms over a 100 ns simulation trajectory. Stable fluctuations and plateauing of the curves indicate that the complexes reached structural equilibrium, confirming the stability of the binding. **(b,e,h)**: These graphs show the RMSF values per residue, highlighting the local flexibility of the protein chains during the simulation. Most residues exhibit low fluctuation values (typically below 0.3 nm, indicating a rigid and stable structural core. Higher peaks correspond to flexible loop regions or terminal ends that do not compromise the overall integrity of the docking. **(c,f,i)**: These plots represent the Radius of Gyration (Rg), which measures the structural compactness of the vaccine-receptor complexes over time. The relatively steady and consistent R_g_ values throughout the 100 ns period suggest that the protein complexes remained stably folded and compact. No significant deviations were observed, indicating that the binding does not cause structural unfolding.

MD simulations were used to estimate the final vaccine construct’s residual stability and fluctuations, including the three chosen receptors (TLR-2, 4, and 5). As shown in Figure 7, RMSD and RMSF were computed to assess each system’s stability and residual fluctuation, respectively. The simulations for each system ran in 100 ns and produced a range of root mean square deviations (Figures 7A–C and Table 8). The stability observed during the 100 ns simulation indicates that the complex is energetically favorable and structurally consistent. However, it is important to note that structural stability *in silico* is a preliminary indicator of potential immune recognition and does not substitute for *in vitro* assays required to confirm functional immune activation. Residual fluctuation (RMSF) was observed with a few higher fluctuating residues that were outside the allowed range (Figures 7D–F and Table 7). We examined the radius of gyration (Rg) to examine the compactness of protein structure throughout the MD simulation. The sustained structural compactness (Rg) and stable RMSD profiles over the 100 ns simulation demonstrate that the 757-amino acid construct maintains a stable conformation in a physiological-like environment, further supporting its biological feasibility and structural integrity despite its large size.

**TABLE 8:** The Average values of root mean square deviation (RMSD), root mean square fluctuation (RMSF) and radius of gyration (Rg) of the TLR receptors 2, 4 and 5 in apo form and TLR-vaccine construct complexes.

Receptor	RMSD (nm)		RMSF (nm)		Rg (nm)	
	Apo form	Complex with vaccine	Apo form	Complex with vaccine	Apo form	Complex with vaccine
TLR2	0.283	0.511	0.166	0.295	3.141	3.616
TLR4	0.282	0.491	0.146	0.309	3.142	3.491
TLR5	1.065	1.364	0.543	0.517	4.363	4.343

#### 3.2.10 Immune simulation

**FIGURE 8:**
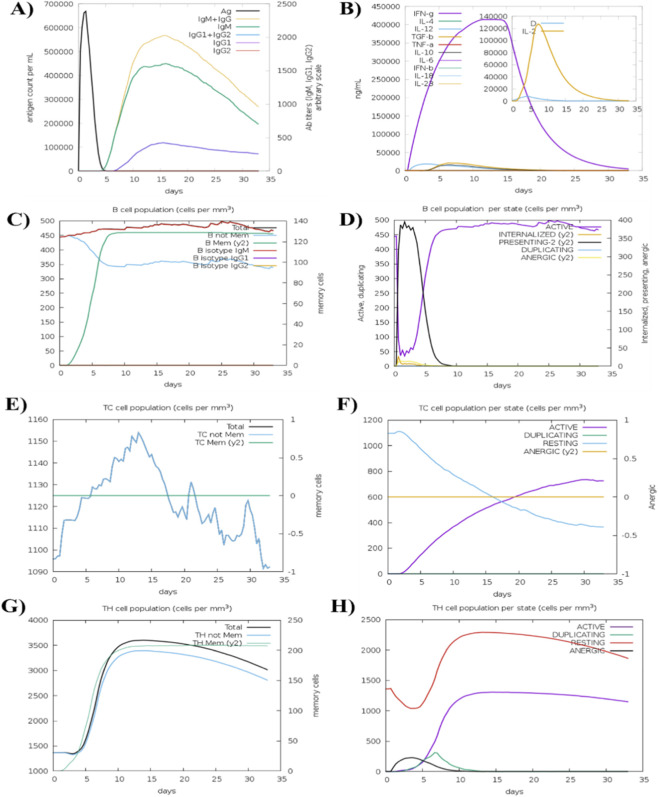
In silico immune response profile following vaccine administration. The simulation shows a robust primary response followed by significant secondary and tertiary antibody titers (IgG and IgM). The cytokine panel highlights elevated levels of IFN-γ and IL-2, suggesting the induction of a protective cell-mediated immune memory against *H. pylori*. **(A)** Increased immunoglobulin production in response to the vaccine injection, indicating an immune response; **(B)** The levels of cytokines, measured by the Simpson index, following the three vaccine injections; **(C)** The population dynamics of B cells following the three vaccine injections, provide insights into the immune response generated by the vaccine; **(D)** B-lymphocytes population per entity-state (i.e., showing counts for active, presenting on class-II, internalized the Ag, duplicating and anergic. **(E)** CD8 T-cytotoxic lymphocytes Count (Total and memory shown). **(F)** The populations of T-cytotoxic cells in different states following the vaccine injections, indicating their involvement in the immune response elicited by the vaccine. **(G)** The TH memory cell population, shows its changes over time. The memory cell population increases specifically after the third injection, while the total cell population continues to increase with each injection. **(H)** The evolution of the TH cell population, with each vaccine injection, over the course of the immune simulation. However, the population decreases when the concentration of antigen decreases.

The simulation results demonstrated a significant increase in the secondary and tertiary immune responses following the second and third doses (Figure 8). There was a sharp rise in IgG1 + IgG2 and IgM antibody titers after each injection, with a corresponding decrease in antigen concentration, indicating the development of an effective humoral memory response. Furthermore, the levels of IFN-γ and IL-2 remained elevated throughout the simulation, supporting the induction of a Th1-biased cellular immune response necessary for the clearance of *H. pylori* (Table 9). These simulation results represent the theoretical immune response under idealized conditions. It is important to note that *in silico* models may not fully replicate the complexities of the human immune system, such as host-specific genetic variability in HLA expression and the varying kinetics of antigen processing in different physiological compartments. The observed increase in memory B-cells and T-cells in the simulation (Figure 6) provides a computational basis for long-term immunity. The high IFN-γ production is particularly significant, as this cytokine is a key mediator in the gastric mucosal response to *H. pylori*.

**TABLE 9:** Summary table of simulation parameters.

Parameter	Setting
Number of injections	3
Interval	4 weeks (84 time steps)
Time steps	1, 84, 168
Total simulation duration	1,050 steps (∼350 days)
Antigen volume	1,000 units

#### 3.2.11 Vaccine peptide design: codon optimization for expression analysis

**FIGURE 9:**
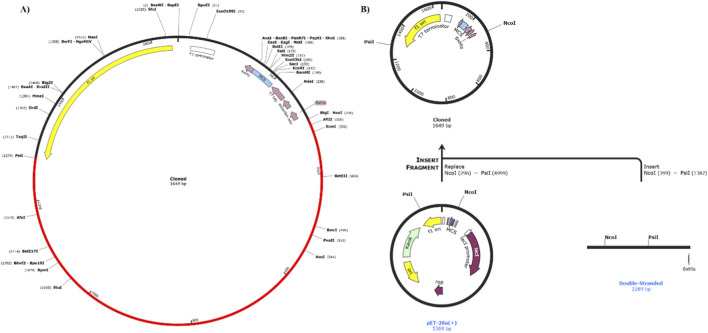
**(A)** The optimized sequence was cloned in pET-28a (+) vector to make a recombinant plasmid after being amplified through *in silico* PCR by using SnapGene software. **(B)** In silico PCR amplification of vaccine construct followed by addition of restriction sites and cloning in pET-28a (+) vector.

For an effective vaccine expression, the *E. coli* expression system is an essential phase in the *in silico* cloning process. Adjuvants and linkers appropriate for prioritized B- and T-cell epitopes were used in the construction of vaccines. Each vaccination’s nucleotide sequence was used as an input (individually) in the Java codon adaptation program (JCat) to modify the codons to fit the mostly sequenced prokaryotic species. Protein output was maximized by codon optimization by the Java codon adaptation tool (JCat). Based on the CAI of the optimized codon, which had a GC content of 48.04% and a length of 2,289 nucleotides, the sequence before and after adaption had a CAI of 0.93. After adding restriction sites (NcoI and PsiI) at the 5′ and 3′ ends, the pET28a (+) vector’s optimized nucleotide sequence was cloned. Figure 9 illustrates the vector, restriction sites, and the vaccine’s overall construct. To address potential challenges in expressing a protein of 83.8 kDa, the sequence was optimized for the *E. coli* K-12 strain. The high Codon Adaptation Index (CAI) of 0.93 and balanced GC content (48.04%) suggest efficient translation and high-yield expression in standard recombinant systems.

## 4 Discussion

The prevalence of *H. pylori* infection is rising continuously in developed and developing nations. Multiple studies suggest that a persistent infection might be the root cause of gastric cancer. Chronic gastritis, multifocal atrophic gastritis, lesions, gastric dysplasia, and rarely intestinal metaplasia are caused by further *H. pylori* infection (Ghosh et al., 2021). There is currently no safe and effective vaccine for *H. pylori* infection. Antibiotic usage is costly and risky, as it continuously produces resistant microorganisms while controlling infectious diseases. An effective means of preventing diseases in a large population is vaccination. However, cutting-edge research has made it possible to design “subunit vaccines,” which are composed of certain pathogenic protein sequences from bacteria and can elicit an immune response, in contrast to conventional vaccination methods (Khan et al., 2019).

Using the subtractive proteomics approach in combination with immuno-informatics, the target sequences for the subunit vaccine against *H. pylori* were identified from the *H. pylori* proteome. This has gained increasing appeal for developing a low-cost, effective vaccination. Prioritizing possible vaccine candidates requires a virulence factor that may substantially infect the host (Hassan et al., 2016). It has also been noted that attributes including subcellular localization, lack of transmembrane helices, allergenicity, and antigenicity may aid in screening possible vaccine candidates (Baseer et al., 2017). The core proteome was extensively investigated with subtractive proteomics approach to identify vaccine candidates that was non-homologous, non-redundant, non-allergic, virulent, and antigenic. A potential candidate for a vaccine design was found to be the lipid A-4′phosphatase (LpxF) protein. The choice of LpxF over more traditional antigens like UreB or VacA is justified by its indispensable role in *H. pylori* survival within the hostile gastric environment. While UreB is vital for acid neutralization, *H. pylori* possess multiple backup mechanisms for pH homeostasis; however, the lipid A modification mediated by LpxF is a unique and critical checkpoint specifically required for resisting host cationic antimicrobial peptides (CAMPs) (Cullen et al., 2011; Needham and Trent, 2013). LpxF modifies lipid A in the outer membrane LPS, enabling *H. pylori* colonization by evading host innate immunity and antimicrobial peptides, as shown by Cullen et al. (2011). While LpxF is localized in the inner membrane, it remains a viable vaccine target because internal bacterial proteins are released during natural cell turnover or immune-mediated lysis and are subsequently processed by professional antigen-presenting cells (APCs) for MHC presentation.

Our multi-epitope approach, which incorporates LpxF epitopes, effectively shifts the focus from merely neutralizing toxins (like VacA) to actively dismantling the bacterium’s primary defense against the host’s innate immune system. This shift represents a significant improvement over single-antigen designs by reducing the likelihood of bacterial persistence through immune-evasion mutations, as LpxF is highly conserved and essential for membrane integrity (Sutton and Boag, 2019).

While the recombinant MEV construct is relatively large at 83.81 kDa, several factors support its biological feasibility. The low instability index (22.06) and negative GRAVY score (−0.022) suggest that the protein is both stable and soluble, which are critical for overcoming common expression challenges like inclusion body formation. Furthermore, the high CAI (0.93) achieved through codon optimization for *E. coli* suggests that the translation machinery will be efficient, potentially mitigating issues related to the construct’s length. Although the 83.81 kDa size is large for a subunit vaccine, its high stability index, hydrophilic nature, and successful *in silico* expression profiling suggest that it is biologically feasible. The use of specialized linkers and codon optimization provides a robust strategy to overcome challenges related to protein folding, solubility, and antigen processing. Tetra- and hexaacylated lipid A species lose their 4′-phosphate group when exposed to LpxF, which lacks the activity of either 1-phosphatase or Kdo hydrolase. The absence of the 4′-phosphate group affords the bacteria resistance to the host’s cationic antimicrobial peptides (CAMP), which is necessary for the bacteria to colonize the stomach over the long term and helps them evade the host’s innate immune system (Cullen et al., 2011). It has been demonstrated that LpxE and LpxF dephosphorylate lipid-A in *H. pylori*, reducing hTLR4-MD2 activation and resisting CAMPs; LpxF having the greatest effect in both scenarios. For *H. pylori* to colonise a mammalian host, phosphate groups must be eliminated from the lipid A backbone. Research also shows how important the lipid makeup of membranes is to the pathophysiology of Gram-negative bacteria. It provides compelling proof of the vital function lipid A plays in pathogenicity (Cullen et al., 2011). LpxF is key to its ability to colonize a mammalian host. The suitability of inner membrane proteins like LpxF as vaccine antigens is supported by their ability to induce robust T-cell mediated responses. Following the proteolytic degradation of the pathogen by APCs, LpxF-derived epitopes are presented *via* MHC-I and MHC-II pathways, priming the Th1-biased and memory responses observed in our immune simulations (Cullen et al., 2011; Leone et al., 2013). However, the transition from *in silico* prediction to *in vivo* efficacy involves overcoming significant biological hurdles. Consequently, while our simulation indicates a robust systemic immune potential, the actual effectiveness of the vaccine will depend on its ability to overcome *H. pylori*-induced immunosuppression and successfully prime a localized mucosal response (Sutton and Boag, 2019).

The immunogenic effectiveness of the LpxF protein was thoroughly examined using web servers, and a potent subunit vaccine design was designed. The B- and T-lymphocyte epitopes were also identified using the selected protein sequence. T cell receptors (TCR) on T lymphocytes typically produce the immune response with response to MHC-I and MHC-II molecules, antigen-presenting cells (APCs), or MHC-bound antigens. The following human alleles were used to collect the HTL MHCII epitopes: These include HLA-DRB1*15:01, HLA-DRB4*01:01, HLA-DRB3*01:01, HLA-DRB5*01:01, HLA-DRB1*03:01, HLA-DRB3*02:02, and HLA-DRB1*07:01. Gastric cancer linked to *H. pylori*, stomach ulcers, gastritis, and other diseases have been linked to the expression of these alleles (Azuma et al., 1994; Yoshitake et al., 1999; Watanabe et al., 2006). It was assured that the epitopes would effectively be presented to T-cell receptors by selecting those with a high affinity for these alleles. IFN-γ may or may not protect the host from *H. pylori* infection (Sayi et al., 2009). To enhance our process of selecting epitopes, we determined which MHCII epitopes induce IFN-γ and then included those epitopes in the candidate vaccine (Table 4). Computational evidence indicates that the interaction of our vaccine construct with these TLRs triggers downstream MyD88-dependent signaling, leading to the high production of IFN-γ and IL-2. These cytokines are well-documented mediators of the gastric mucosal response and are significantly correlated with the reduction of *H. pylori* colonization in animal models.

Immuno-informatics study of the *H. pylori* proteome indicates that the proposed vaccine protein covers a broad range of high affinities MHC Class I, II, and B-cell linear epitope based on physio-chemical characteristics and structural qualities. Additionally, the production of memory B cells and plasma offers defense against specific antigens or antigens associated with infections in the future. Here, the adjuvant aids in including important MHC I and II binding epitopes in the vaccine’s final suggested design to improve host protection. The gastric epithelial cells react to the infection by activating several signalling pathways. By interacting with Toll-like receptors (TLRs), they serve as the first point of contact for *H. pylori* adhesion (Wilson and Crabtree, 2007). Lipoproteins, peptidoglycans, and lipopolysaccharides (LPS) are among the microbial products that TLRs recognize, and their identification is crucial for controlling both innate and adaptive immune responses (Akira et al., 2006). TLR2 also identifies variants of *H. pylori*’s LPS that are structurally distinct from those identified by TLR4 (Yokota et al., 2007), in addition to TLR4 (Matsuura, 2013) being the primary receptor for LPS. Following bacterial recognition, the adaptor molecule MyD88, associated with TLR2 and TLR4, activates them both, triggering the mitogen-activating protein kinase (MAPK) signaling cascade. The transcription factor NF-κB is then activated at this point, resulting in the rapid production of proinflammatory cytokines, chemokines and their receptors, and interleukins, as well as inducible nitric oxide synthase (iNOS) (Ding et al., 2005; Re and Strominger, 2004). While *H. pylori* LPS is a weak activator of TLR4 compared to other Gram-negative bacteria, both TLR2 and TLR4 are essential for the induction of pro-inflammatory cytokines like IL-8 and TNF-α in gastric epithelial cells. Research by Cullen et al. (2011) specifically highlights that *H. pylori* modifies its lipid A (*via* LpxF) to evade TLR4-mediated detection, suggesting that a vaccine targeting this evasion mechanism (like our LpxF-based MEV) must demonstrate strong binding to TLR4 to restore and enhance innate immune recognition. Although *H. pylori* flagellin (FlaA) is modified to limit its activation of TLR5, this receptor remains a critical target for mucosal vaccine adjuvants. The recruitment of TLR5 signaling is vital for the maturation of dendritic cells and the subsequent priming of Th1 and Th2 adaptive immune responses necessary for long-term protection. These factors can potentially become carcinogenic when they are increased because they cause a significant inflammatory response of the mucosa, characterized as persistently active gastritis (Akira and Takeda, 2004; Fukata and Abreu, 2009). To evade the host’s innate immune response, pathogenic bacteria alter their lipid A in several ways. The lipid A domain, which identifies and activates the human Toll-like receptor 4-myeloid differentiation factor 2 (hTLR4-MD2) complex, is responsible for the endotoxic properties of lipoprotein LPS (Kim et al., 2007). It has been demonstrated that the human Toll-like receptor 2 (hTLR2) can detect atypical forms of LPS in addition to certain conserved bacterial components, such as lipoteichoic acid and lipoproteins (Erridge, 2010). The presence of the hTLR4-MD2 complex on various cell types allows for the recognition of fragmented LPS during infection by Gram-negative pathogens. The interactions between TLR2, TLR4, and TLR5 and the constructed vaccine were assessed using molecular docking, and the stability of the docking complexes was evaluated using MD simulations. *H. pylori*’s lipid A is modified by LpxF (lipid A 4′-phosphatase), removing the 4′-phosphate and leading to tetra-acylated, hypo-phosphorylated LPS. Standard Gram-negative LPS activates TLR4 *via* MD-2 binding to hexa-acylated lipid A; *H. pylori*’s version shows 100–1000-fold lower potency, minimizing NF-κB activation, IL-8/TNF-α secretion, and dendritic cell maturation. Flagellin is similarly amidated at residue 57 (γ-glutamylation), blocking TLR5 dimerization and cytokine induction. Result: Weak innate signaling allows asymptomatic colonization. Vaccine counter: LpxF epitopes are internalized by APCs, processed for MHC-I/II presentation; docking shows vaccine-TLR complexes (e.g., −1213 kcal/mol TLR5), priming adaptive responses upstream of evasion (Fan et al., 2024).

Molecular docking studies were used to analyze the vaccine’s pattern of interaction with TLR2, TLR4, and TLR5 (Figures 6A–C). 28 hydrogen bonds and two salt bridges were generated during the interaction between TLR4 and the vaccine construct, according to the docking analysis (Figure 6A and Supplementary Table S9). The docked complex demonstrates the formation of salt bridges between the vaccine’s Glu183 and Asp131 and TLR4’s Lys615, Arg23. Likewise, the docking study between TLR2 and the vaccine construct revealed that 23 hydrogen bonds and three salt bridges were generated during the contact (Figure 6B and Supplementary Table S9). Here, the salt bridges were generated between our vaccine’s Lys160, Lys159, and Asp131 and TLR2’s Glu608, Asp580, and His431 correspondingly. Further, TLR5 molecular interaction with vaccine construct reveals 5 hydrogen bonds, the residue Arg34 of vaccine forms two hydrogen bonds with Asn510 and Gln559 of TLR5, Tyr 57 vaccine construct with Asn512 of TLR5, Gln80 of vaccine construct with Arg537of TLR5 (Figure 6C and Supplementary Table S9). It must be noted that a primary limitation of this study is the reliance on computational docking and simulations. Although these methods provide a robust framework for assessing the structural feasibility of the vaccine-TLR complex, they cannot fully simulate the complex biochemical cascades involved in receptor-mediated immune activation. Therefore, these results should be interpreted as evidence of binding potential, which warrants further experimental validation through cytokine profiling and cell-based signaling assays.

The vaccine’s stability was demonstrated by the extremely slight changes in the RMSD graph that were seen throughout the 100-ns molecular dynamics simulation of the vaccine construct (Figures 7A–C). The vaccine construct’s strong flexibility was indicated by the RMSF graph’s high peak regions (Figures 7D–F). One of the most crucial methods for ensuring that the vaccine is stable is to simulate it *in vivo* using molecular dynamics simulation, or MDS. The RMSD and RMSF values from our MDS are comparable to those from other research teams who have examined the vaccine candidate’s flexibility and stability under conditions that closely resemble those found *in vivo* (Nezafat et al., 2014; Chauhan et al., 2019). Compared to TLR4 and TLR5 receptors, the vaccine construct in complex with TLR2 exhibited minimal fluctuations but generally showed high compactness (Figures 7G–I). The low binding energy and stable RMSD profile observed in our MD simulations are consistent with successful vaccine-receptor interactions reported in similar *in silico* studies for mucosal pathogens (Razzak et al., 2025).

The vaccination candidate was initially validated by immunoreactivity and serological analysis, which led to a series of immune stimulation studies. After repeated exposure to antigens, immune simulation findings showed an increasing level of IgG1, IgG2, and IgM response (Figures 8A–H), which was like the previously published research (Muhsen et al., 2018). We also detected the memory T- and B-cell populations. It's interesting to note that after the first injection, our design was able to increase the levels of IFN-γ and IL-2, and that these cytokines' production peaked with repeated injections. Maintaining an elevated amount of TH cells and Ig production is indicative of an optimal humoral response (Bao et al., 2014). Cheng et al. (2022) showed that the C-ImmSimm server’s anticipated outcome may produce a promising and consistent result and is in good agreement with real-world trials. As anticipated by the C-ImmSimm server, they got significant levels of IFN-γ+ T cells and IgG/IgG2a antibodies from the immunized mice. This validates our findings, as the significant amount of IFN-γ and IgG response from our constructed vaccination was also anticipated by the C-immSimm server. The Th1-biased T-cell responses observed in immune simulations characterized by high IFN-γ and IL-2 levels align with experimental evidence suggesting that cell-mediated immunity is a critical component of a protective response against *H. pylori*. The strategic focus on LpxF addresses the limitations of earlier vaccine candidates by targeting a conserved mechanism of immune evasion rather than highly variable surface antigens (Sutton and Boag, 2019). A limitation of the current immune simulation approach is the omission of factors such as chronic inflammatory background and the bacterial niche’s specific inhibitory factors. Future experimental studies are essential to evaluate the vaccine’s performance against the pathogen’s ability to downregulate MHC-II expression and interfere with the maturation of professional APCs.

The linear vaccine construct was reverse-translated into its distinct cDNA sequence, and codon optimization was performed to ensure efficient expression in the *E. coli* host. The vaccine candidate may be efficiently expressed in the host *E. coli*, as shown by its GC content of 48.04%. To express the vaccine in a bacterial system, it was additionally added to the expression vector pET-28a (+) for *in silico* cloning. Foroutan et al. (2020) optimized the vaccine’s codon before it’s *in vitro* production using a similar methodology. Following the last injection, our constructed vaccine elicited immune responses necessary to eliminate the antigen on secondary exposure, as validated by immune simulation studies. Comparably, multiepitope vaccines against *H. pylori* have recently been designed using the immunoinformatic approach to vaccine design (Urrutia-Baca et al., 2019; Jafari and Mahmoodi, 2021; Keshri et al., 2023). The multi-epitope-based vaccine construct has all the parameters within the ideal and recommended ranges to be considered a strong vaccine candidate, as demonstrated by the bioinformatics analysis and evaluation above. Despite the promising *in silico* results regarding binding affinity and complex stability, the functional biological activity of the vaccine construct, particularly its ability to trigger specific downstream signaling *via* TLR pathways, remains to be validated through experimental immunization studies. Nevertheless, experimental validation is still required to ascertain the vaccine’s efficacy in humans.

### 4.1 Limitations and future directions

Despite the rigorous multi-level computational validation of the LpxF-based vaccine, several limitations must be acknowledged. First, *in silico* predictions of protein folding and binding affinities, while highly advanced, cannot fully replicate the complex physiological environment of the human gastric mucosa or the diverse proteases present in the digestive tract. Second, the predicted immunogenicity is based on established HLA allele frequencies and experimental data from other antigens; however, the actual magnitude of the IgG and IgA response in humans can only be determined through wet-lab experiments. Lastly, while the vaccine targets a highly conserved enzyme (LpxF), the possibility of rare *H. pylori* strains developing compensatory mechanisms or alternative lipid A modification pathways cannot be entirely ruled out.

### 4.2 Pathway to clinical development

The transition of this vaccine candidate from a computational model to clinical application follows a structured developmental pipeline. The immediate next step involves the recombinant expression of the MEV construct in a microbial system (e.g., *E. coli* BL21) followed by affinity chromatography purification using the built-in 6x-Histidine tag. Preclinical evaluation will require challenge studies in murine models, such as C57BL/6 mice, to assess the vaccine’s ability to reduce *H. pylori* colonization and inflammatory cytokines. Following successful safety and immunogenicity results in animals, the candidate would progress to Phase I clinical trials to evaluate safety and dose-escalation in humans, followed by Phase II and III trials to establish protective efficacy in endemic populations. This systematic approach is essential to confirm the vaccine’s potential to curb the global burden of gastric cancer and antibiotic-resistant *H. pylori* infections.

## 5 Conclusion

The present study targeted the cell inner membrane protein LpxF to design a multi-epitope chimaera vaccination against *H. pylori*. This focus on LpxF provides a distinct advantage over earlier vaccine candidates by targeting a conserved mechanism of immune evasion, thereby addressing the limitations of strain variability associated with other common antigens. There are epitopes on T- and B-cells that can express IFN-γ, producing a strong humoral response and cell-mediated immunity. The designed vaccine’s antigenic and allergenic profiles, along with certain physicochemical properties, are also well-characterized. According to the immunological simulation and docking investigation, there was a strong binding with TLR responses. To effectively refine the designed vaccine for use in the future, we also carried out *in silico* cloning. We predict that this vaccine will provide a favourable outcome in controlling *H. pylori* infection; however, prior to human administration, *in-vitro* and *in-vivo* investigations are necessary to confirm the vaccine’s efficacy, immunogenicity, and foremost, host safety. While these computational findings provide a strong foundation for vaccine development, they must be interpreted with caution. The idealized nature of immune simulations necessitates subsequent *in vivo* validation to assess the impact of mucosal tolerance and host-pathogen interactions on the vaccine’s actual protective efficacy.

## Data Availability

The datasets presented in this study can be found in online repositories. The names of the repository/repositories and accession number(s) can be found in the article/Supplementary Material.
