# Supplementary material for: Designing and immuno-informatics evaluation of a multi-epitope vaccine targeting lipoprotein A-4′-phosphatase (LpxF) for Helicobacter pylori infection control
Source: Front Bioinform. 2026 Mar 18;6:1779654. doi: 10.3389/fbinf.2026.1779654 (PMC13039028; doi:10.3389/fbinf.2026.1779654)
Supplement: Supplementary file 1 [file Table1.docx]

**Supplementary File**

**Designing and Immuno-informatics Evaluation of a Multi-Epitope Vaccine Targeting Lipoprotein A-4'-Phosphatase (LpxF) for Helicobacter pylori Infection Control**

Pavan Gollapalli ^*^, Tamizh Selvan Gnanasekaran

**Table S1: List of functional hypothetical proteins (542 proteins) of the known domain and/or families and their GO terms were selected for analysis using GO FEAT 1.0 server**

| **UniProt ID** | **Protein Name** |
| --- | --- |
| A0A2J9KJE1 | Uncharacterized protein |
| A0A2I8VE69 | DedA family protein |
| A0A2J9KJ83 | Pantothenate kinase |
| A0A2J9KJ58 | 3'-5' exonuclease |
| O24882 | Uncharacterized protein |
| A0A2J9KL74 | Carbon-nitrogen hydrolase family protein |
| A0A0M9W8H0 | Energy transducer TonB |
| A0A2J9KLU4 | PMT_2 domain-containing protein |
| A0A0L0PLP4 | Membrane protein |
| A0A2J9KJ20 | Alpha/beta hydrolase |
| O25454 | Conserved hypothetical integral membrane protein |
| A0A2J9KJN7 | M23 family peptidase |
| A0A024C6E6 | Integral membrane protein |
| K2L826 | Uncharacterized protein |
| A0A2J9KJ54 | ATP-binding protein |
| O25111 | Uncharacterized protein |
| A0A2J9KIK2 | Restriction endonuclease |
| O26105 | Uncharacterized protein |
| A0A0B2EVL1 | Membrane protein |
| A0A2J9KL87 | Histidine kinase |
| A0A2J9KL67 | Purine-nucleoside phosphorylase |
| A0A2J9KJ93 | Insulinase family protein |
| A0A2J9KL76 | Type II restriction endonuclease |
| A0A0B2ED21 | Uncharacterized protein |
| A0A2J9KJS9 | Lipopolysaccharide heptosyltransferase family protein |
| A0A2J9KM10 | MATE family efflux transporter |
| A0A2J9KKD9 | Cytochrome C oxidase subunit III |
| A0A1V3BFR1 | Arginine biosynthesis bifunctional protein ArgJ |
| A0A2J9KKD2 | Flagellar FliJ protein |
| A0A2J9KIE5 | Mechanosensitive ion channel family protein |
| A0A246LQ15 | Guanine permease |
| A0A2J9KJB4 | Aminodeoxyfutalosine synthase |
| A3R4F2 | Arginase |
| A0A2J9KIV3 | MBL fold metallo-hydrolase |
| A0A2J9KL34 | Flagellar basal body rod protein FlgB |
| A0A2J9KLW3 | ABC transporter substrate-binding protein |
| A0A2J9KLR0 | NADH-quinone oxidoreductase subunit G |
| O25631 | Exonuclease VII-like protein (XseA) |
| A0A0M0MU00 | Cytochrome b6-F complex iron-sulfur subunit |
| A0A2J9KK27 | UDP-glucose 4-epimerase |
| O25937 | DNA-directed DNA polymerase |
| A0A2J9KI47 | MFS transporter |
| A0A2J9KJD7 | Penicillin-insensitive transglycosylase |
| A0A2J9KM16 | Uncharacterized protein |
| A0A2J9KJC4 | HyaD/HybD family hydrogenase maturation endopeptidase |
| A0A2J9KL93 | TolC family protein |
| A0A2J9KKN0 | L-lactate permease |
| A0A2J9KID9 | Peptidylprolyl isomerase |
| A0A2J9KIE6 | Amino acid permease |
| A0A2J9KK12 | Flagellar M-ring protein |
| A0A2J9KLH2 | Restriction endonuclease subunit S |
| A0A2J9KKP0 | CcoQ/FixQ family Cbb3-type cytochrome c oxidase assembly chaperone |
| A0A2J9KKV1 | Carbamoyltransferase HypF |
| A0A2J9KM44 | tRNA threonylcarbamoyladenosine dehydratase |
| A0A2J9KI46 | Flagellar hook-associated protein 1 |
| K2LPI6 | Cytochrome-c oxidase |
| A0A2J9KIR9 | ADP-glyceromanno-heptose 6-epimerase |
| A0A2J9KL45 | Dihydrofolate synthase/folylpolyglutamate synthase |
| A0A2J9KKU4 | GDP-mannose 4,6-dehydratase |
| A0A2J9KIU4 | Coenzyme A biosynthesis bifunctional protein CoaBC |
| O25063 | Toxin-like outer membrane protein |
| A0A2J9KLZ0 | FAD-binding oxidoreductase |
| A0A3S6H550 | tRNA 2-thiocytidine biosynthesis protein TtcA |
| A0A2J9KK91 | Peptide ABC transporter ATP-binding protein |
| A0A2J9KJF6 | 2-oxoglutarate synthase subunit alpha |
| A0A2J9KKZ7 | YchF/TatD family DNA exonuclease |
| A0A2J9KIR8 | RNA-binding protein |
| A0A2J9KL10 | Carboxynorspermidine/carboxyspermidine decarboxylase |
| E8QKY3 | ABC transporter, permease |
| A0A2J9KJK6 | 23S rRNA (Guanosine(2251)-2'-O)-methyltransferase RlmB |
| A0A2J9KKT3 | Sodium/proline symporter |
| A0A2J9KKT5 | Agmatine deiminase |
| A0A0J8H4V9 | Arginine decarboxylase |
| A0A2J9KJ01 | ATP-binding protein |
| A0A2J9KJM7 | Cag pathogenicity island protein |
| A0A2J9KJ11 | ABC transporter permease |
| A0A2J9KK54 | Uncharacterized protein |
| A0A2J9KIB2 | O-antigen ligase family protein |
| A0A2J9KIA7 | Flagellar motor switch protein FliN |
| A0A2J9KK11 | HemK family protein methyltransferase |
| A0A2J9KM45 | ATP-dependent metallopeptidase FtsH/Yme1/Tma family protein |
| A0A2J9KIJ4 | MFS transporter |
| A0A2J9KK02 | LptF/LptG family permease |
| A0A2J9KK98 | ABC transporter ATP-binding protein |
| A0A2J9KM43 | Lipoprotein |
| A0A2J9KJ42 | t(6)A37 threonylcarbamoyladenosine biosynthesis protein TsaE |
| A0A2J9KJ77 | Aminotransferase |
| A0A2J9KK87 | Lipopolysaccharide heptosyltransferase I |
| O25653 | Site-specific recombinase |
| A0A2J9KKU7 | VirB8 family protein |
| A0A2J9KJR0 | Oligoendopeptidase F |
| A0A2J9KKB2 | Lipid A biosynthesis lauroyl acyltransferase |
| A0A2J9KLV7 | Phosphoribosyltransferase |
| A0A2J9KL47 | TonB-dependent receptor |
| A0A2J9KL73 | Uncharacterized protein |
| A0A2J9KJC6 | Uncharacterized protein |
| A0A2I8V4H8 | Lipopolysaccharide heptosyltransferase II |
| A0A2J9KL84 | Restriction endonuclease |
| A0A2J9KJA0 | Aminotran_1_2 domain-containing protein |
| A0A2J9KJF9 | ABC transporter ATP-binding protein |
| A0A083YCR3 | Membrane protein |
| A0A086RVM5 | ABC transporter ATP-binding protein |
| A0A2J9KL22 | Glycosyltransferase family 8 protein |
| A0A2J9KII9 | ATP/GTP-binding protein |
| A0A0B2EAB8 | Uncharacterized protein |
| A0A2J9KL17 | DNA helicase |
| A0A246LSC7 | Helix-turn-helix domain-containing protein |
| A0A2J9KK61 | DUF1669 domain-containing protein |
| A0A2J9KM38 | Uncharacterized protein |
| A0A2J9KJ64 | DNA polymerase III subunit gamma/tau |
| A0A2J9KIJ3 | Sodium:proton antiporter |
| A0A2J9KHT9 | Restriction endonuclease |
| A0A2J9KK22 | Uncharacterized protein |
| A0A1A9GWB4 | Uncharacterized protein |
| A0A2J9KKD0 | YbhB/YbcL family Raf kinase inhibitor-like protein |
| A0A2J9KK15 | Cytochrome C biogenesis protein |
| A0A2J9KJ21 | HIT family hydrolase |
| A0A2J9KLT5 | Uncharacterized protein |
| A0A2J9KL71 | M23 family peptidase |
| A0A246LS00 | Alpha-1,2-fucosyltransferase |
| A0A2J9KJ55 | Lysine transporter |
| A0A2J9KJZ3 | Uncharacterized protein |
| A0A2J9KK94 | Mechanosensitive ion channel family protein |
| A0A024C648 | VirB2 type IV secretion protein |
| O25591 | DUF1524 domain-containing protein |
| A0A2J9KIR5 | Uncharacterized protein |
| O25663 | Periplasmic serine endoprotease DegP-like |
| A0A2J9KLY0 | RDD family protein |
| A0A2J9KLT3 | Lipopolysaccharide heptosyltransferase family protein |
| A0A024C7W2 | DedA family protein |
| A0A2J9KHX9 | Amino acid ABC transporter permease |
| A0A2J9KLT9 | NADH-quinone oxidoreductase subunit L |
| O25923 | Type III restriction enzyme R protein |
| A0A2J9KLG9 | Uncharacterized protein |
| A0A2J9KIG5 | ATPase |
| A0A2J9KJI1 | Uncharacterized protein |
| A0A2J9KJ06 | Labile enterotoxin output A |
| A0A2J9KL00 | Glycosyl transferase |
| A0A2J9KJE3 | Uncharacterized protein |
| A0A2J9KJ79 | Uncharacterized protein |
| O25815 | LPS-assembly protein LptD |
| A0A293TFY6 | Uncharacterized protein |
| A0A2J9KJ19 | Peptidase_M23 domain-containing protein |
| O25110 | Uncharacterized protein |
| A0A2J9KKF2 | Uncharacterized protein |
| O25331 | Toxin-like outer membrane protein |
| O25659 | Uncharacterized protein |
| A0A2J9KIE4 | Uncharacterized protein |
| A0A2A6W348 | Pyruvate ferredoxin oxidoreductase |
| A0A2J9KI78 | DNA-binding response regulator |
| O25658 | 7-alpha-hydroxysteroid dehydrogenase (HdhA) |
| A0A2J9KJ60 | (Fe-S)-binding protein |
| O25394 | Flagellar biosynthetic protein FliP |
| A0A2J9KHR1 | TerC family protein |
| A0A2J9KJ22 | Uncharacterized protein |
| A0A2J9KJL9 | Peptidase |
| A0A2J9KJY3 | Protein kinase |
| A0A2J9KJ58 | 3'-5' exonuclease |
| A0A2J9KLX0 | HAD family hydrolase |
| A0A2J9KJQ0 | Glycolate oxidase subunit GlcD |
| A0A2J9KKG0 | Uncharacterized protein |
| O25507 | Uncharacterized protein |
| A0A3Q8AM52 | Two component response regulator |
| A0A2J9KJH1 | Energy transducer TonB |
| A0A2J9KJI0 | Uncharacterized protein |
| A0A2J9KII2 | Uncharacterized protein |
| A0A2J9KKV2 | Restriction endonuclease |
| A0A1V3ARZ8 | Integral membrane protein |
| A0A2J9KLZ6 | Uncharacterized protein |
| A0A2J9KKS9 | Glycosyltransferase |
| O34754 | IS605 transposase (TnpA) |
| O34550 | IS200 insertion sequence from SARA17 |
| O34540 | Uncharacterized protein |
| O34945 | IS605 transposase (TnpB) |
| O34491 | IS605 transposase (TnpB) |
| A0A2J9KLU5 | NADH-quinone oxidoreductase subunit N |
| A0A2J9KIK3 | 7-carboxy-7-deazaguanine synthase |
| A0A2J9KKW6 | Transporter |
| O25262 | Cag pathogenicity island protein (Cag7) |
| A0A2J9KHY0 | KH domain-containing protein |
| A0A2J9KJA3 | UTP--glucose-1-phosphate uridylyltransferase |
| A0A2J9KJ24 | YkgJ family cysteine cluster protein |
| A0A2J9KJR3 | Molybdenum ABC transporter ATP-binding protein |
| A0A2J9KKL5 | Adenine-specific DNA glycosylase |
| A0A2J9KKK0 | ABC transporter ATP-binding protein |
| A0A2J9KJZ7 | Thiol:disulfide interchange protein DsbC |
| A0A2J9KI79 | TlyA family rRNA (Cytidine-2'-O)-methyltransferase |
| A0A2J9KJV2 | Neuraminyllactose-binding hemagglutinin |
| A0A2J9KKK7 | HAAAP family serine/threonine permease |
| A0A2J9KKF1 | Probable membrane transporter protein |
| A0A2J9KIZ2 | N-acetylmuramoyl-L-alanine amidase |
| A0A0B2E588 | CopD family copper resistance protein |
| A0A2J9KIJ7 | Polyprenyl synthetase family protein |
| A0A2J9KJ30 | UDP-N-acetylmuramoyl-tripeptide--D-alanyl-D-alanine ligase |
| A0A2J9KJ98 | O-phosphoserine phosphohydrolase |
| A0A2J9KJJ1 | Sodium:calcium antiporter |
| A0A2J9KKX9 | ATP-dependent Clp protease ATP-binding subunit |
| A0A086RWJ2 | Flagellar basal-body rod protein FlgG |
| A0A2J9KK21 | Biotin carboxylase |
| A0A2J9KIZ8 | Restriction endonuclease subunit S |
| A0A2J9KKS0 | Bifunctional metallophosphatase/5'-nucleotidase |
| A0A0L0PBC8 | N-carbamoylputrescine amidase |
| A0A2J9KJ70 | Probable membrane transporter protein |
| A0A2J9KIH1 | CusA/CzcA family heavy metal efflux RND transporter |
| A0A0B2E3F3 | 2-nitropropane dioxygenase |
| A0A2A6T0L7 | Cytochrome b |
| A0A2J9KK84 | Flagellar biosynthesis protein FlgL |
| A0A2J9KLX5 | DNA polymerase III subunit delta |
| A0A2J9KJR7 | Catalase-related peroxidase |
| A0A2J9KKT8 | Methyltransferase |
| A0A2J9KKK2 | Molybdopterin molybdenumtransferase |
| A0A2J9KJC0 | Ni/Fe hydrogenase |
| A0A2J9KLZ8 | Site-specific DNA-methyltransferase (adenine-specific) |
| A0A2J9KK79 | Indole-3-glycerol-phosphate synthase |
| A0A2J9KIP7 | Basal-body rod modification protein FlgD |
| A0A2J9KJW2 | VirB8 family protein |
| A0A2J9KIU8 | Phosphatase PAP2 family protein |
| A0A2J9KIT8 | DsbD_2 domain-containing protein |
| A0A2J9KI37 | Riboflavin biosynthesis protein |
| A0A2J9KJN0 | Cag pathogenicity island protein |
| A0A2J9KKY5 | ATP-binding protein |
| O25366 | Alpha-(1,3)-fucosyltransferase |
| A0A2J9KM17 | Site-specific DNA-methyltransferase (adenine-specific) |
| A0A2J9KL28 | M23 family metallopeptidase |
| A0A2J9KJU1 | Protein phosphatase |
| A0A0B2E6R3 | MFS transporter |
| A0A3S6H3J4 | Lipopolysaccharide export system ATP-binding protein LptB |
| A0A2J9KJG0 | LTA synthase family protein |
| A0A2J9KJS2 | Transporter |
| O25954 | Type I restriction enzyme S protein (HsdS) |
| A0A2J9KK39 | DNA-protecting protein DprA |
| A0A2J9KL24 | 3-deoxy-D-manno-octulosonate 8-phosphate phosphatase KdsC |
| A0A2J9KL92 | Heavy metal translocating P-type ATPase |
| A0A2J9KL58 | Penicillin-binding protein 2 |
| A0A246LSE3 | 2-ketoisovalerate ferredoxin oxidoreductase |
| A0A2J9KHZ7 | Amino acid ABC transporter ATP-binding protein |
| Q7BK04 | Type IV secretion system protein |
| A0A2J9KLC0 | VRR-NUC domain-containing protein |
| A0A2J9KI28 | ATP F0F1 synthase subunit B |
| A0A2J9KLI7 | Alanine dehydrogenase |
| A0A2J9KJN9 | Molybdenum transport system permease |
| A0A2J9KJZ6 | M48 family peptidase |
| A0A2J9KK13 | D-3-phosphoglycerate dehydrogenase |
| A0A2J9KKX5 | DNA (Cytosine-5-)-methyltransferase |
| A0A2J9KII4 | Amino acid carrier protein |
| A0A2J9KJH6 | Endonuclease III |
| A0A2J9KLV0 | NADH-quinone oxidoreductase subunit J |
| A0A2J9KI53 | Beta-lactamase |
| A0A2J9KI40 | NAD(P)-dependent alcohol dehydrogenase |
| A0A2J9KJ80 | Oxidoreductase |
| A0A2J9KJN6 | Sodium:calcium antiporter |
| A0A2J9KJS5 | Cation:proton antiporter |
| A0A2J9KLB1 | YjgP/YjgQ family permease |
| A0A2I8V1X9 | SLC13 family permease |
| A0A2J9KK92 | Cytochrome C biogenesis protein |
| A0A2J9KKL4 | 1,4-dihydroxy-6-naphtoate synthase |
| A0A2J9KK32 | NAD(P)-dependent oxidoreductase |
| A0A2J9KKA7 | Polyprenyl synthetase family protein |
| A0A2J9KLC8 | Formyltetrahydrofolate deformylase |
| A0A0B2DT78 | ABC transporter permease |
| A0A2J9KIP4 | ABC transporter ATP-binding protein |
| A0A2J9KJB9 | 3-methyladenine DNA glycosylase |
| A0A3S6H3S8 | Pyruvate synthase subunit PorD |
| A0A246LR03 | Efflux RND transporter periplasmic adaptor subunit |
| A0A2J9KL89 | Flagella basal body P-ring formation protein FlgA |
| A0A2I8V2H1 | Alpha-(1,3)-fucosyltransferase |
| A0A2J9KK66 | ABC transporter permease |
| A0A2J9KI66 | Restriction endonuclease |
| A0A2J9KM15 | ABC transporter ATP-binding protein |
| A0A2J9KJ99 | CCA tRNA nucleotidyltransferase |
| A0A2J9KLH9 | DNA/RNA non-specific endonuclease |
| A0A0L0PH87 | DUF417 domain-containing protein |
| A0A2J9KJE8 | Endolytic murein transglycosylase |
| A0A2J9KJ46 | Phosphoribosyltransferase |
| A0A2J9KJI5 | Sodium:calcium antiporter |
| A0A2J9KJN5 | Beta-lactamase |
| O25487 | Iron(III) dicitrate transport protein (FecA) |
| A0A1A9GWA4 | Cytochrome-c oxidase, cbb3-type subunit II |
| A0A2J9KLS4 | Thiamine diphosphokinase |
| A0A2J9KID2 | RNA polymerase sigma factor FliA |
| A0A2J9KJG3 | 2-oxoglutarate:acceptor oxidoreductase |
| A0A2J9KM22 | Amino acid ABC transporter permease |
| A0A2J9KJL1 | Cag pathogenicity island protein |
| A0A2J9KJ69 | HD family hydrolase |
| A0A2J9KJF8 | 2-oxoglutarate:acceptor oxidoreductase |
| A0A2J9KIV1 | ABC transporter ATP-binding protein |
| A0A3S6H489 | 2-oxoglutarate oxidoreductase subunit KorB |
| A0A2J9KID6 | 5'-3' exonuclease |
| A0A2J9KI75 | Prokaryotic metallothionein family protein |
| A0A2J9KIZ6 | ABC transporter permease |
| A0A2J9KHV3 | Methyltransferase |
| A0A2J9KLE7 | Ribonuclease N |
| A0A0S1XQD1 | Exodeoxyribonuclease III |
| A0A3Q8AWH2 | Ferredoxin |
| A0A2J9KI61 | Transketolase |
| A0A2J9KJ51 | Phosphatidylglycerophosphatase A |
| A0A2J9KJ38 | TIGR00366 family protein |
| A0A2J9KKP4 | Type-2 restriction enzyme |
| A0A2J9KIX9 | 16S rRNA (Guanine(966)-N(2))-methyltransferase RsmD |
| A0A2J9KKN2 | Iron-sulfur cluster-binding protein |
| A0A2J9KLT7 | NADH-quinone oxidoreductase |
| A0A2J9KKS1 | Hydroxyacid dehydrogenase |
| A0A2J9KK86 | Carbon-nitrogen hydrolase family protein |
| A0A2J9KJX7 | UDP-2,3-diacylglucosamine diphosphatase |
| A0A2J9KJS4 | Molybdate ABC transporter substrate-binding protein |
| A0A2J9KJP3 | Neuraminyllactose-binding hemagglutinin |
| A0A2J9KK64 | ABC transporter substrate-binding protein |
| O25798 | Carbonic anhydrase |
| A0A2J9KKX0 | Hydrogenase expression/formation protein HypE |
| A0A2J9KJH2 | Uncharacterized protein |
| A0A2J9KJB3 | YggT family protein |
| A0A2J9KJU0 | DNA topoisomerase |
| A0A2J9KKC1 | Guanosine-5'-triphosphate, 3'-diphosphate pyrophosphate |
| A0A2J9KJG1 | Flagellar motor switch protein FliN |
| A0A2J9KL98 | ABC transporter permease |
| A0A2J9KM92 | Competence protein |
| A0A2J9KJG4 | AcrB/AcrD/AcrF family protein |
| A0A086RQ79 | NADH-quinone oxidoreductase subunit |
| A0A2J9KKU9 | Chemotaxis protein |
| O24997 | ATPase_AAA_core domain-containing protein |
| A0A2J9KIQ2 | Outer membrane protein |
| A0A2I8VDK0 | Cell shape-determining protein MreB |
| A0A2J9KIG8 | Integrase |
| A0A2J9KI11 | ATP synthase F(1) sector subunit delta |
| A0A2J9KLB0 | ComF family protein |
| A0A2J9KJ67 | Ligand-gated channel |
| A0A2J9KJ27 | Sodium:proton antiporter |
| O25211 | Type I site-specific deoxyribonuclease |
| A0A246LR08 | 3-oxoacyl-[acyl-carrier-protein] reductase |
| O25240 | Sodium-and chloride-dependent transporter |
| A0A2J9KK03 | Molybdopterin guanine dinucleotide-containing S/N-oxide reductase |
| A0A2J9KL27 | Flagellar basal-body rod protein FlgC |
| O25980 | Uncharacterized protein |
| A0A2J9KK18 | Uncharacterized protein |
| A0A2J9KLA1 | Ubiquinone/menaquinone biosynthesis C-methyltransferase UbiE |
| A0A2J9KI62 | Flagellar basal body protein |
| A0A2J9KID0 | DUF1887 domain-containing protein |
| A0A2J9KJQ1 | Potassium channel protein |
| A0A2J9KI09 | MFS transporter |
| O25517 | Type I restriction enzyme R Protein |
| A0A2J9KLW0 | Uroporphyrinogen-III synthase |
| A0A2J9KIC4 | CDP-diacylglycerol--glycerol-3-phosphate 3-phosphatidyltransferase |
| A0A2J9KJI2 | Ribosome-binding ATPase YchF |
| A0A2J9KL15 | PAP2 family protein |
| A0A2J9KJG6 | Sialidase domain-containing protein |
| A0A2J9KM13 | Cytosine-specific methyltransferase |
| A0A2J9KLF6 | Glycosyltransferase family 8 protein |
| O24983 | Uncharacterized protein |
| A0A2J9KM63 | Methylated-DNA--protein-cysteine methyltransferase |
| A0A2J9KM36 | 3-oxoacyl-[acyl-carrier-protein] synthase 2 |
| A0A2J9KIP9 | Hydrogenase maturation factor |
| O25922 | Methyltransferase |
| A0A2J9KLL6 | Rod shape-determining protein MreC |
| A0A3S6H2D0 | Flagellar protein FliL |
| A0A2J9KKB3 | Methyltransferase |
| A0A2J9KIS1 | Site-specific DNA-methyltransferase (adenine-specific) |
| A0A2J9KKA0 | Peptide ABC transporter permease |
| A0A2J9KJP7 | NUDIX domain-containing protein |
| A0A2J9KJD1 | Ni/Fe-hydrogenase, b-type cytochrome subunit |
| A0A2J9KJR5 | Restriction endonuclease subunit S |
| A0A2J9KLH6 | Site-specific DNA-methyltransferase (adenine-specific) |
| O26037 | Conserved hypothetical ATP-binding protein |
| A0A2J9KLP4 | ATP-binding protein |
| A0A2J9KJ57 | Acetyl-CoA C-acetyltransferase |
| O26006 | Type IIS restriction enzyme R protein (BCGIB) |
| A0A246LRA9 | Uncharacterized protein |
| A0A0B2DZJ4 | Microcin ABC transporter permease |
| A0A2J9KIH4 | Acyl carrier protein |
| A0A2J9KLT2 | NADH-quinone oxidoreductase subunit M |
| A0A2J9KLC2 | Cytochrome C biogenesis protein CcsA |
| A0A2J9KLU0 | Aminopyrimidine aminohydrolase |
| A0A2J9KJI8 | Sialidase |
| A0A2J9KJW0 | Glycosyltransferase family 4 protein |
| A0A2J9KL32 | Lipopolysaccharide transport periplasmic protein LptA |
| A0A2J9KL32 | Lipopolysaccharide transport periplasmic protein LptA |
| A0A2J9KKM1 | L-lactate permease |
| A0A2J9KKE4 | SulP family inorganic anion transporter |
| A0A2J9KIG1 | Conjugal transfer protein TraG |
| O26021 | Conserved hypothetical integral membrane protein |
| A0A2J9KL50 | DEAD/DEAH box helicase |
| A0A2J9KL60 | Cytochrome c oxidase accessory protein CcoG |
| A0A2J9KL66 | Lipoprotein |
| A0A2J9KJM5 | Transporter |
| A0A2J9KL77 | tRNA1(Val) (Adenine(37)-N6)-methyltransferase |
| A0A2J9KKQ4 | Chemotaxis protein |
| O25185 | AAA_11 domain-containing protein |
| A0A2J9KKT9 | Methyltransferase |
| A0A2J9KK63 | DUF2156 domain-containing protein |
| A0A2J9KL48 | Uncharacterized protein |
| A0A2J9KIQ3 | Tat pathway signal protein |
| A0A1Q2RAQ3 | ABC transporter ATP-binding protein |
| A0A0M8NKA4 | Glycoside hydrolase family 43 |
| A0A2J9KKZ0 | Mannose-1-phosphate guanylyltransferase |
| A0A2J9KKG9 | Nitrogen fixation protein NifU |
| A0A2J9KIL7 | GNAT family N-acetyltransferase |
| A0A2J9KLS9 | Biotin synthase |
| A0A2J9KI27 | Biotin--[acetyl-CoA-carboxylase] ligase |
| A0A2J9KLM2 | CusA/CzcA family heavy metal efflux RND transporter |
| A0A2J9KKA2 | Potassium transporter TrkA |
| A0A2J9KJP8 | Restriction endonuclease |
| A0A024C162 | Ribosomal silencing factor RsfS |
| A0A2J9KKU5 | Conjugal transfer protein TrbL |
| A0A2J9KL18 | Uncharacterized protein |
| A0A3S6H258 | Heat shock protein HspR |
| A0A2J9KJR2 | Site-specific DNA-methyltransferase (adenine-specific) |
| A0A2J9KHQ0 | S41 family peptidase |
| A0A2J9KJC9 | DEAD/DEAH box helicase |
| A0A2J9KKB7 | Protein disulfide-isomerase |
| A0A3Q8B6G8 | Sporulation initiation inhibitor protein Soj |
| A0A2J9KJ76 | Hydantoinase/oxoprolinase family protein |
| G5EAR8 | GDP-L-fucose synthase |
| A0A2J9KJB7 | Beta-lactamase |
| A0A2J9KIM3 | DNA helicase |
| O25226 | Adenine specific DNA methyltransferase (MFOKI) |
| O25519 | Type I restriction enzyme S protein (HsdS) |
| A0A3S6H546 | Fe/S biogenesis protein NfuA |
| A0A2J9KI67 | Glycosyltransferase family 8 protein |
| A0A2J9KII3 | 3-deoxy-D-manno-octulosonic acid transferase |
| A0A0B2E5U0 | Flagellar motor switch protein FliM |
| A0A2J9KI51 | OmpA family protein |
| A0A2J9KLC5 | Signal peptide peptidase SppA |
| A0A2J9KJT1 | Site-specific DNA-methyltransferase (adenine-specific) |
| A0A2J9KLG6 | Type I restriction enzyme R Protein |
| A0A2J9KLB5 | HlyC/CorC family transporter |
| A0A2J9KJH4 | Ribose 5-phosphate isomerase B |
| A0A2J9KKX7 | Cytosine-specific methyltransferase |
| A0A2J9KJE4 | ABC transporter ATP-binding protein |
| A0A2J9KKW2 | Bifunctional protein PutA |
| A0A2J9KI25 | Gamma-glutamyltransferase |
| A0A2I8V0S5 | 4-hydroxybenzoate octaprenyltransferase |
| A0A2J9KIB8 | DnaJ family protein |
| A0A2J9KJ14 | RelA/SpoT family protein |
| A0A2J9KK74 | DUF4149 domain-containing protein |
| A0A1A9H0F7 | Biotin carboxyl carrier protein of acetyl-CoA carboxylase |
| A0A083YDP5 | Flagellar biosynthetic protein FliR |
| A0A2J9KLN9 | Nicotinamide riboside transporter PnuC |
| A0A2J9KL70 | Sodium/glutamate symporter |
| A0A0B2DRT0 | Pyruvate ferredoxin oxidoreductase |
| A0A083YG54 | Cag pathogenicity island protein |
| A0A2J9KL75 | Dihydroneopterin aldolase |
| A0A2J9KK68 | Alginate_lyase domain-containing protein |
| A0A2J9KLK4 | Uncharacterized protein |
| A0A024C1S3 | ABC transporter ATP-binding protein |
| A0A2J9KI68 | 2-dehydro-3-deoxy-phosphogluconate aldolase |
| A0A2J9KIB4 | Replication-associated recombination protein A |
| A0A3S6H3S6 | Flagellar secretion chaperone FliS |
| A0A2J9KL80 | ABC transporter permease |
| A0A2J9KJV8 | VirB4 family type IV secretion/conjugal transfer ATPase |
| A0A2J9KM81 | Prephenate dehydrogenase |
| A0A2J9KIN5 | Flagellar basal body protein |
| A0A2A6W9Q8 | Uncharacterized protein |
| A0A1Q2PLL9 | ABC transporter |
| A0A246LSJ0 | Type I glyceraldehyde-3-phosphate dehydrogenase |
| A0A024C0W8 | Cation:proton antiporter |
| A0A2J9KL25 | Penicillin-binding protein 2 |
| A0A2J9KM03 | Modification methylase |
| A0A2J9KKP7 | Malonyl CoA-acyl carrier protein transacylase |
| A0A2J9KJM8 | VirB8 family protein |
| A0A060CZ58 | VirB3 type IV secretion protein |
| A0A2J9KLZ7 | Heme chaperone HemW |
| J9RVI3 | Phospholipase A1 |
| A0A2I8V5P3 | ABC transporter permease |
| A0A2J9KJM6 | Cag pathogenicity island protein Cag1 |
| A0A0B2DYW2 | ABC transporter ATP-binding protein |
| A0A2J9KLJ4 | Methyltransferase |
| A0A2J9KL61 | Site-specific DNA-methyltransferase (adenine-specific) |
| A0A2J9KKP5 | Phospho-2-dehydro-3-deoxyheptonate aldolase |
| A0A2J9KI07 | Amino acid ABC transporter permease |
| A0A2J9KI59 | ABC transporter ATP-binding protein |
| A0A2J9KJB1 | Nickel-dependent hydrogenase large subunit |
| A0A2J9KIV6 | Glycosyl transferase |
| A0A2J9KK10 | Uncharacterized protein |
| A0A2J9KKI8 | Glycosyltransferase family 8 protein |
| A0A2J9KJ72 | Cyclic dehypoxanthine futalosine synthase |
| A0A2J9KLC9 | DNA helicase |
| A0A2J9KIZ3 | Chorismate dehydratase |
| A0A2I8UZ11 | Acetone carboxylase subunit alpha |
| A0A2J9KIS6 | Osmoprotection protein |
| A0A2J9KK25 | Single-stranded-DNA-specific exonuclease RecJ |
| A0A2J9KL83 | Bifunctional diaminohydroxyphosphoribosylaminopyrimidine deaminase/5-amino-6-(5-phosphoribosylamino)uracil reductase |
| A0A2J9KLQ6 | Phosphomannomutase/phosphoglucomutase |
| A0A1Q2PTS7 | Acetyl-coenzyme A carboxylase carboxyl transferase subunit beta |
| A0A2J9KKQ1 | Methyl-accepting chemotaxis protein |
| A0A2J9KKA1 | Chorismate mutase |
| A0A2J9KIH7 | RIP metalloprotease RseP |
| A0A2J9KJX8 | Class I SAM-dependent methyltransferase |
| A0A2J9KK9 | Bifunctional aminodeoxychorismate synthase component I/aminotransferase |
| A0A2J9KJW4 | Aminotransferase class V-fold PLP-dependent enzyme |
| A0A2J9KJE7 | Site-specific DNA-methyltransferase (adenine-specific) |
| A0A2J9KIE8 | Insulinase family protein |
| A0A2J9KKJ9 | Uncharacterized protein |
| A0A2J9KJ53 | Uncharacterized protein |
| A0A2J9KLV9 | RDD family protein |
| A0A2J9KK28 | DUF1294 domain-containing protein |
| Q7BGI8 | NAD(P)H nitroreductase |
| O25168 | Uncharacterized protein |
| A0A2J9KM64 | Uncharacterized protein |
| A0A2J9KKJ6 | Peptidase |
| A0A2J9KKM2 | Uncharacterized protein |
| A0A2J9KI82 | Exonuclease |
| O25810 | Ulcer-associated gene restriction endonuclease (IceA) |
| A0A2J9KLH8 | Uncharacterized protein |
| A0A2J9KII8 | Toxin |
| O25380 | Site-specific DNA-methyltransferase (adenine-specific) |
| O25908 | Putative adenine specific DNA methyltransferase |
| A0A2J9KIN6 | Uncharacterized protein |
| A0A2J9KKD6 | CopG family transcriptional regulator |
| O25433 | G domain-containing protein |
| A0A3S6H3C8 | Outer membrane efflux protein |
| A0A2J9KJZ0 | SPOR domain-containing protein |
| A0A2J9KLQ3 | 5'-nucleotidase, lipoprotein e(P4) family |
| A0A2J9KIL9 | Uncharacterized protein |
| A0A2J9KJB2 | TPR_REGION domain-containing protein |
| A0A024C3S1 | Membrane protein |
| A0A2J9KJ25 | 5-formyltetrahydrofolate cyclo-ligase |
| A0A2J9KIX7 | Class I SAM-dependent methyltransferase |
| A0A2J9KLQ8 | Lipopolysaccharide heptosyltransferase family protein |
| A0A2J9KJI6 | Site-2 protease family protein |
| A0A2J9KJF0 | Disulfide bond formation protein B |
| A0A2J9KJ95 | Uncharacterized protein |
| A0A2J9KLI3 | Methyltransferase |
| A0A2I8V959 | SH3 domain-containing protein |
| A0A2J9KJ40 | Outer membrane beta-barrel protein |
| O25969 | Uncharacterized protein |
| A0A2J9KLR2 | Pantothenate kinase |
| A0A2J9KJD6 | ABC transporter substrate-binding protein |
| A0A2J9KKV3 | DNA translocase FtsK |
| A0A2J9KKL1 | Uncharacterized protein |
| A0A2J9KK96 | Uncharacterized protein |
| A0A2J9KIW3 | DUF3943 domain-containing protein |
| A0A2J9KII7 | DUF1049 domain-containing protein |
| A0A2J9KKV6 | DUF1523 domain-containing protein |
| A0A2J9KK46 | Uncharacterized protein |
| A0A246LRK4 | DUF2393 domain-containing protein |
| A0A2J9KJE6 | Methyl-accepting chemotaxis protein |
| A0A2J9KIV9 | MoeB/ThiF family adenylyltransferase |
| A0A2J9KIF2 | GTPase |
| A0A2J9KLA3 | Anaphase-promoting protein |
| A0A2J9KI02 | MFS transporter |
| A0A060CZR8 | VirB3 type IV secretion protein |
| J0DPJ6 | LPS-assembly protein LptD |
| O25379 | Uncharacterized protein |

**Table S2: List of non-homologous hypothetical proteins (412 proteins) analysed for functional domain/family using BLASTp tool.**

| **UniProt ID** | **Protein Name** | Gene |
| --- | --- | --- |
| A0A2J9KJE1 | Uncharacterized protein | C2840_03030 |
| A0A2I8VE69 | DedA family protein | C2840_06010 |
| A0A2J9KJ83 | Pantothenate kinase | C2840_03510 |
| A0A2J9KJ58 | 3'-5' exonuclease | C2840_03540 |
| O24882 | Uncharacterized protein | HP_0041 |
| A0A2J9KL74 | Carbon-nitrogen hydrolase family protein | C2840_07710 |
| A0A0M9W8H0 | Energy transducer TonB | AM498_03975 |
| A0A2J9KLU4 | PMT_2 domain-containing protein | C2840_06415 |
| A0A0L0PLP4 | Membrane protein | ACM23_00790 |
| A0A2J9KJ20 | Alpha/beta hydrolase | C2840_03770 |
| A0A2J9KJN7 | M23 family peptidase | C2840_02635 |
| A0A024C6E6 | Integral membrane protein | AC785_06050 |
| K2L826 | Uncharacterized protein | OUO_0536 |
| A0A2J9KJ54 | ATP-binding protein | C2840_03725 |
| O25111 | Uncharacterized protein | HP_0344 |
| A0A2J9KIK2 | Restriction endonuclease | C2840_04655 |
| O26105 | Uncharacterized protein | HP_1586 |
| A0A0B2EVL1 | Membrane protein | AC785_07295 |
| A0A2J9KL87 | Histidine kinase | C2840_07770 |
| A0A2J9KL67 | Purine-nucleoside phosphorylase | C2840_07940 |
| A0A2J9KL76 | Type II restriction endonuclease | C2840_07980 |
| A0A0B2ED21 | Uncharacterized protein | ACM24_04610 |
| A0A2J9KJS9 | Lipopolysaccharide heptosyltransferase family protein | C2840_02500 |
| A0A2J9KM10 | MATE family efflux transporter | C2840_06125 |
| A0A2J9KKD9 | Cytochrome C oxidase subunit III | C2840_01230 |
| A0A1V3BFR1 | Arginine biosynthesis bifunctional protein ArgJ | B0X29_06755 |
| A0A2J9KKD2 | Flagellar FliJ protein | C2840_01330 |
| A0A2J9KIE5 | Mechanosensitive ion channel family protein | C2840_05065 |
| A0A2J9KJB4 | Aminodeoxyfutalosine synthase | mqnE |
| A3R4F2 | Arginase | rocF |
| A0A2J9KIV3 | MBL fold metallo-hydrolase | C2840_04150 |
| A0A2J9KL34 | Flagellar basal body rod protein FlgB | flgB |
| A0A2J9KLW3 | ABC transporter substrate-binding protein | C2840_06505 |
| A0A2J9KLR0 | NADH-quinone oxidoreductase subunit G | C2840_06575 |
| A0A0M0MU00 | Cytochrome b6-F complex iron-sulfur subunit | fbcF |
| O25937 | DNA-directed DNA polymerase | HP_1387 |
| A0A2J9KI47 | MFS transporter | C2840_05630 |
| A0A2J9KJD7 | Penicillin-insensitive transglycosylase | C2840_03110 |
| A0A2J9KJC4 | HyaD/HybD family hydrogenase maturation endopeptidase | hybD |
| A0A2J9KL93 | TolC family protein | C2840_07750 |
| A0A2J9KKN0 | L-lactate permease | C2840_00755 |
| A0A2J9KID9 | Peptidylprolyl isomerase | C2840_05020 |
| A0A2J9KIE6 | Amino acid permease | C2840_05250 |
| A0A2J9KK12 | Flagellar M-ring protein | C2840_01815 |
| A0A2J9KLH2 | Restriction endonuclease subunit S | C2840_07190 |
| A0A2J9KKP0 | CcoQ/FixQ family Cbb3-type cytochrome c oxidase assembly chaperone | C2840_00780 |
| A0A2J9KKV1 | Carbamoyltransferase HypF | hypF |
| A0A2J9KI46 | Flagellar hook-associated protein 1 | C2840_05785 |
| O25063 | Toxin-like outer membrane protein | HP_0289 |
| A0A3S6H550 | tRNA 2-thiocytidine biosynthesis protein TtcA | ttcA |
| A0A2J9KJF6 | 2-oxoglutarate synthase subunit alpha | C2840_03065 |
| A0A2J9KL10 | Carboxynorspermidine/carboxyspermidine decarboxylase | nspC |
| E8QKY3 | ABC transporter, permease | HPGAM_01755 |
| A0A2J9KKT5 | Agmatine deiminase | C2840_00265 |
| A0A0J8H4V9 | Arginine decarboxylase | speA |
| A0A2J9KJ01 | ATP-binding protein | C2840_04045 |
| A0A2J9KJM7 | Cag pathogenicity island protein | B0X45_05370 |
| A0A2J9KJ11 | ABC transporter permease | C2840_03830 |
| A0A2J9KK54 | Uncharacterized protein | C2840_01590 |
| A0A2J9KIB2 | O-antigen ligase family protein | C2840_05360 |
| A0A2J9KIA7 | Flagellar motor switch protein FliN | fliN |
| A0A2J9KIJ4 | MFS transporter | C2840_04790 |
| A0A2J9KK02 | LptF/LptG family permease | C2840_01875 |
| A0A2J9KM43 | Lipoprotein | C2840_08125 |
| A0A2J9KJ42 | t(6)A37 threonylcarbamoyladenosine biosynthesis protein TsaE | C2840_03665 |
| A0A2J9KK87 | Lipopolysaccharide heptosyltransferase I | waaC |
| O25653 | Site-specific recombinase | HP_1009 |
| A0A2J9KKU7 | VirB8 family protein | C2840_00220 |
| A0A2J9KJR0 | Oligoendopeptidase F | C2840_02445 |
| A0A2J9KKB2 | Lipid A biosynthesis lauroyl acyltransferase | C2840_01450 |
| A0A2J9KLV7 | Phosphoribosyltransferase | C2840_06315 |
| A0A2J9KL47 | TonB-dependent receptor | C2840_07860 |
| A0A2J9KL73 | Uncharacterized protein | C2840_07875 |
| A0A2J9KJC6 | Uncharacterized protein | C2840_03305 |
| A0A2I8V4H8 | Lipopolysaccharide heptosyltransferase II | waaF |
| A0A2J9KL84 | Restriction endonuclease | C2840_07800 |
| A0A083YCR3 | Membrane protein | B0X28_02655 |
| A0A2J9KL22 | Glycosyltransferase family 8 protein | C2840_08195 |
| A0A2J9KII9 | ATP/GTP-binding protein | C2840_04960 |
| A0A0B2EAB8 | Uncharacterized protein | AA976_03165 |
| A0A2J9KL17 | DNA helicase | C2840_08065 |
| A0A246LSC7 | Helix-turn-helix domain-containing protein | C2840_07050 |
| A0A2J9KM38 | Uncharacterized protein | C2840_07180 |
| A0A2J9KIJ3 | Sodium:proton antiporter | C2840_04860 |
| A0A2J9KHT9 | Restriction endonuclease | C2840_07020 |
| A0A2J9KK22 | Uncharacterized protein | C2840_01755 |
| A0A1A9GWB4 | Uncharacterized protein | AA971_00705 |
| A0A2J9KKD0 | YbhB/YbcL family Raf kinase inhibitor-like protein | C2840_01140 |
| A0A2J9KK15 | Cytochrome C biogenesis protein | C2840_01955 |
| A0A2J9KJ21 | HIT family hydrolase | C2840_03780 |
| A0A2J9KLT5 | Uncharacterized protein | C2840_06695 |
| A0A2J9KL71 | M23 family peptidase | C2840_08020 |
| A0A2J9KJ55 | Lysine transporter | C2840_03675 |
| A0A2J9KJZ3 | Uncharacterized protein | C2840_01940 |
| A0A2J9KK94 | Mechanosensitive ion channel family protein | C2840_01470 |
| A0A024C648 | VirB2 type IV secretion protein | virB2 |
| O25591 | DUF1524 domain-containing protein | HP_0937 |
| A0A2J9KIR5 | Uncharacterized protein | C2840_04370 |
| A0A2J9KLY0 | RDD family protein | C2840_06325 |
| A0A2J9KLT3 | Lipopolysaccharide heptosyltransferase family protein | C2840_06665 |
| A0A024C7W2 | DedA family protein | yohD_2 |
| A0A2J9KHX9 | Amino acid ABC transporter permease | C2840_06045 |
| O25923 | Type III restriction enzyme R protein | HP_1371 |
| A0A2J9KLG9 | Uncharacterized protein | C2840_07470 |
| A0A2J9KIG5 | ATPase | C2840_04950 |
| A0A2J9KJI1 | Uncharacterized protein | C2840_02895 |
| A0A2J9KJ06 | Labile enterotoxin output A | C2840_03735 |
| A0A2J9KL00 | Glycosyl transferase | C2840_08210 |
| A0A2J9KJE3 | Uncharacterized protein | C2840_03235 |
| A0A2J9KJ79 | Uncharacterized protein | C2840_03590 |
| O25815 | LPS-assembly protein LptD | lptD |
| A0A293TFY6 | Uncharacterized protein | BB469_02105 |
| O25110 | Uncharacterized protein | HP_0343 |
| A0A2J9KKF2 | Uncharacterized protein | C2840_01220 |
| O25331 | Toxin-like outer membrane protein | HP_0610 |
| O25659 | Uncharacterized protein | HP_1015 |
| A0A2J9KIE4 | Uncharacterized protein | C2840_05050 |
| A0A2A6W348 | Pyruvate ferredoxin oxidoreductase | BB397_07925 |
| A0A2J9KI78 | DNA-binding response regulator | C2840_05380 |
| A0A2J9KJ60 | (Fe-S)-binding protein | C2840_03450 |
| O25394 | Flagellar biosynthetic protein FliP | HP_0685 |
| A0A2J9KHR1 | TerC family protein | C2840_06980 |
| A0A2J9KJ22 | Uncharacterized protein | C2840_03940 |
| A0A2J9KJL9 | Peptidase | C2840_02695 |
| A0A2J9KJ58 | 3'-5' exonuclease | C2840_03540 |
| A0A2J9KLX0 | HAD family hydrolase | C2840_06270 |
| A0A2J9KKG0 | Uncharacterized protein | C2840_00970 |
| O25507 | Uncharacterized protein | HP_0836 |
| A0A3Q8AM52 | Two component response regulator | HPYLPMSS1_00411 |
| A0A2J9KJH1 | Energy transducer TonB | C2840_03025 |
| A0A2J9KJI0 | Uncharacterized protein | C2840_03050 |
| A0A2J9KII2 | Uncharacterized protein | C2840_05070 |
| A0A2J9KKV2 | Restriction endonuclease | C2840_00285 |
| A0A1V3ARZ8 | Integral membrane protein | B0X29_06125 |
| A0A2J9KLZ6 | Uncharacterized protein | C2840_04990 |
| A0A2J9KKS9 | Glycosyltransferase | C2840_00545 |
| O34754 | IS605 transposase (TnpA) | HP_0437 |
| O34550 | IS200 insertion sequence from SARA17 | HP_0414 |
| O34540 | Uncharacterized protein | HP_0427 |
| O34945 | IS605 transposase (TnpB) | HP_0989 |
| O34491 | IS605 transposase (TnpB) | HP_0438 |
| A0A2J9KIK3 | 7-carboxy-7-deazaguanine synthase | queE |
| A0A2J9KKW6 | Transporter | C2840_00105 |
| O25262 | Cag pathogenicity island protein (Cag7) | HP_0527 |
| A0A2J9KHY0 | KH domain-containing protein | C2840_05950 |
| A0A2J9KJA3 | UTP--glucose-1-phosphate uridylyltransferase | galU |
| A0A2J9KJ24 | YkgJ family cysteine cluster protein | C2840_03905 |
| A0A2J9KKK0 | ABC transporter ATP-binding protein | C2840_00940 |
| A0A2J9KJZ7 | Thiol:disulfide interchange protein DsbC | C2840_01950 |
| A0A2J9KI79 | TlyA family rRNA (Cytidine-2'-O)-methyltransferase | C2840_05600 |
| A0A2J9KJV2 | Neuraminyllactose-binding hemagglutinin | C2840_02115 |
| A0A2J9KKK7 | HAAAP family serine/threonine permease | C2840_00715 |
| A0A2J9KKF1 | Probable membrane transporter protein | BXP09_06400 |
| A0A2J9KIZ2 | N-acetylmuramoyl-L-alanine amidase | C2840_03945 |
| A0A0B2E588 | CopD family copper resistance protein | AA973_06810 |
| A0A2J9KJ30 | UDP-N-acetylmuramoyl-tripeptide--D-alanyl-D-alanine ligase | C2840_03775 |
| A0A2J9KJJ1 | Sodium:calcium antiporter | C2840_02840 |
| A0A086RWJ2 | Flagellar basal-body rod protein FlgG | flgG |
| A0A2J9KIZ8 | Restriction endonuclease subunit S | C2840_04035 |
| A0A2J9KKS0 | Bifunctional metallophosphatase/5'-nucleotidase | C2840_00560 |
| A0A2J9KJ70 | Probable membrane transporter protein | C2840_03490 |
| A0A2J9KIH1 | CusA/CzcA family heavy metal efflux RND transporter | C2840_04980 |
| A0A0B2E3F3 | 2-nitropropane dioxygenase | AM497_02530 |
| A0A2J9KK84 | Flagellar biosynthesis protein FlgL | flgL |
| A0A2J9KLX5 | DNA polymerase III subunit delta | C2840_06395 |
| A0A2J9KKT8 | Methyltransferase | C2840_00270 |
| A0A2J9KJC0 | Ni/Fe hydrogenase | C2840_03275 |
| A0A2J9KLZ8 | Site-specific DNA-methyltransferase (adenine-specific) | C2840_04660 |
| A0A2J9KK79 | Indole-3-glycerol-phosphate synthase | C2840_01430 |
| A0A2J9KIP7 | Basal-body rod modification protein FlgD | flgD |
| A0A2J9KJW2 | VirB8 family protein | C2840_02270 |
| A0A2J9KIT8 | DsbD_2 domain-containing protein | C2840_04395 |
| A0A2J9KI37 | Riboflavin biosynthesis protein | C2840_05605 |
| A0A2J9KJN0 | Cag pathogenicity island protein | C2840_02785 |
| A0A2J9KKY5 | ATP-binding protein | C2840_00085 |
| A0A2J9KM17 | Site-specific DNA-methyltransferase (adenine-specific) | C2840_01350 |
| A0A2J9KL28 | M23 family metallopeptidase | C2840_08015 |
| A0A2J9KJU1 | Protein phosphatase | C2840_02240 |
| A0A0B2E6R3 | MFS transporter | B0X51_06465 |
| A0A2J9KJG0 | LTA synthase family protein | C2840_03005 |
| A0A2J9KJS2 | Transporter | C2840_02385 |
| O25954 | Type I restriction enzyme S protein (HsdS) | HP_1404 |
| A0A2J9KK39 | DNA-protecting protein DprA | C2840_08080 |
| A0A2J9KL58 | Penicillin-binding protein 2 |  |
| A0A246LSE3 | 2-ketoisovalerate ferredoxin oxidoreductase | BB394_05455 |
| Q7BK04 | Type IV secretion system protein | cag-alfa |
| A0A2J9KLC0 | VRR-NUC domain-containing protein | C2840_07665 |
| A0A2J9KI28 | ATP F0F1 synthase subunit B | B0X61_02885 |
| A0A2J9KJN9 | Molybdenum transport system permease | modB |
| A0A2J9KKX5 | DNA (Cytosine-5-)-methyltransferase | C2840_00290 |
| A0A2J9KII4 | Amino acid carrier protein | C2840_04820 |
| A0A2J9KLV0 | NADH-quinone oxidoreductase subunit J | C2840_06590 |
| A0A2J9KJ80 | Oxidoreductase | C2840_03495 |
| A0A2J9KJN6 | Sodium:calcium antiporter | C2840_02725 |
| A0A2J9KLB1 | YjgP/YjgQ family permease | C2840_07795 |
| A0A2J9KK92 | Cytochrome C biogenesis protein | C2840_01375 |
| A0A2J9KKL4 | 1,4-dihydroxy-6-naphtoate synthase | mqnD |
| A0A0B2DT78 | ABC transporter permease | AB891_00890 |
| A0A2J9KJB9 | 3-methyladenine DNA glycosylase | C2840_03135 |
| A0A3S6H3S8 | Pyruvate synthase subunit PorD | porD |
| A0A246LR03 | Efflux RND transporter periplasmic adaptor subunit | B0X31_04735 |
| A0A2J9KL89 | Flagella basal body P-ring formation protein FlgA | C2840_07690 |
| A0A2J9KK66 | ABC transporter permease | C2840_01550 |
| A0A2J9KI66 | Restriction endonuclease | C2840_05570 |
| A0A2J9KLH9 | DNA/RNA non-specific endonuclease | C2840_07185 |
| A0A0L0PH87 | DUF417 domain-containing protein | AA971_03745 |
| A0A2J9KJE8 | Endolytic murein transglycosylase | mltG |
| A0A2J9KJ46 | Phosphoribosyltransferase | C2840_03750 |
| A0A2J9KJI5 | Sodium:calcium antiporter | C2840_02815 |
| A0A2J9KJN5 | Beta-lactamase | C2840_02700 |
| O25487 | Iron(III) dicitrate transport protein (FecA) | HP_0807 |
| A0A1A9GWA4 | Cytochrome-c oxidase, cbb3-type subunit II | ccoO |
| A0A2J9KLS4 | Thiamine diphosphokinase | C2840_06705 |
| A0A2J9KID2 | RNA polymerase sigma factor FliA | C2840_05325 |
| A0A2J9KJG3 | 2-oxoglutarate:acceptor oxidoreductase | oorC |
| A0A2J9KM22 | Amino acid ABC transporter permease | C2840_04805 |
| A0A2J9KJL1 | Cag pathogenicity island protein | C2840_02750 |
| A0A2J9KJ69 | HD family hydrolase | C2840_03645 |
| A0A2J9KJF8 | 2-oxoglutarate:acceptor oxidoreductase | C2840_03060 |
| A0A3S6H489 | 2-oxoglutarate oxidoreductase subunit KorB | korB |
| A0A2J9KID6 | 5'-3' exonuclease | C2840_05275 |
| A0A2J9KI75 | Prokaryotic metallothionein family protein | C2840_05485 |
| A0A2J9KIZ6 | ABC transporter permease | C2840_04020 |
| A0A2J9KHV3 | Methyltransferase | C2840_07025 |
| A0A2J9KLE7 | Ribonuclease N | C2840_07310 |
| A0A2J9KJ51 | Phosphatidylglycerophosphatase A | C2840_03760 |
| A0A2J9KJ38 | TIGR00366 family protein | C2840_03565 |
| A0A2J9KKP4 | Type-2 restriction enzyme | C2840_00490 |
| A0A2J9KKN2 | Iron-sulfur cluster-binding protein | C2840_00740 |
| A0A2J9KJX7 | UDP-2,3-diacylglucosamine diphosphatase | C2840_02035 |
| A0A2J9KJS4 | Molybdate ABC transporter substrate-binding protein | modA |
| A0A2J9KJP3 | Neuraminyllactose-binding hemagglutinin | C2840_02570 |
| A0A2J9KK64 | ABC transporter substrate-binding protein | C2840_01540 |
| O25798 | Carbonic anhydrase | HP_1186 |
| A0A2J9KKX0 | Hydrogenase expression/formation protein HypE | hypE |
| A0A2J9KJH2 | Uncharacterized protein | C2840_03010 |
| A0A2J9KJB3 | YggT family protein | C2840_03340 |
| A0A2J9KKC1 | Guanosine-5'-triphosphate, 3'-diphosphate pyrophosphate | gppA |
| A0A2J9KJG1 | Flagellar motor switch protein FliN | fliN |
| A0A2J9KL98 | ABC transporter permease | C2840_07635 |
| A0A2J9KM92 | Competence protein | C2840_07065 |
| A0A2J9KJG4 | AcrB/AcrD/AcrF family protein | C2840_03160 |
| A0A2J9KKU9 | Chemotaxis protein | C2840_00440 |
| O24997 | ATPase_AAA_core domain-containing protein | HP_0205 |
| A0A2J9KIQ2 | Outer membrane protein | C2840_04470 |
| A0A2J9KIG8 | Integrase | C2840_05105 |
| A0A2J9KI11 | ATP synthase F(1) sector subunit delta | C2840_05865 |
| A0A2J9KLB0 | ComF family protein | C2840_07670 |
| A0A2J9KJ67 | Ligand-gated channel | C2840_03530 |
| A0A2J9KJ27 | Sodium:proton antiporter | C2840_03875 |
| A0A2J9KK03 | Molybdopterin guanine dinucleotide-containing S/N-oxide reductase | C2840_02100 |
| A0A2J9KL27 | Flagellar basal-body rod protein FlgC | flgC |
| O25980 | Uncharacterized protein | HP_1439 |
| A0A2J9KK18 | Uncharacterized protein | C2840_01810 |
| A0A2J9KI62 | Flagellar basal body protein | C2840_05635 |
| A0A2J9KID0 | DUF1887 domain-containing protein | C2840_05080 |
| A0A2J9KJQ1 | Potassium channel protein | C2840_02560 |
| A0A2J9KI09 | MFS transporter | C2840_06025 |
| O25517 | Type I restriction enzyme R Protein | HP_0846 |
| A0A2J9KLW0 | Uroporphyrinogen-III synthase | C2840_06360 |
| A0A2J9KL15 | PAP2 family protein | C2840_08205 |
| A0A2J9KJG6 | Sialidase domain-containing protein | C2840_03015 |
| A0A2J9KLF6 | Glycosyltransferase family 8 protein | C2840_07370 |
| O24983 | Uncharacterized protein | HP_0181 |
| A0A2J9KIP9 | Hydrogenase maturation factor | hypD |
| O25922 | Methyltransferase | HP_1370 |
| A0A2J9KLL6 | Rod shape-determining protein MreC | C2840_07130 |
| A0A3S6H2D0 | Flagellar protein FliL | fliL |
| A0A2J9KKB3 | Methyltransferase | C2840_01365 |
| A0A2J9KIS1 | Site-specific DNA-methyltransferase (adenine-specific) | C2840_04335 |
| A0A2J9KKA0 | Peptide ABC transporter permease | C2840_01545 |
| A0A2J9KJD1 | Ni/Fe-hydrogenase, b-type cytochrome subunit | cybH |
| A0A2J9KJR5 | Restriction endonuclease subunit S | C2840_02400 |
| A0A2J9KLH6 | Site-specific DNA-methyltransferase (adenine-specific) | C2840_07290 |
| O26037 | Conserved hypothetical ATP-binding protein | HP_1507 |
| A0A2J9KLP4 | ATP-binding protein | C2840_06860 |
| O26006 | Type IIS restriction enzyme R protein (BCGIB) | HP_1471 |
| A0A246LRA9 | Uncharacterized protein | B0X35_02630 |
| A0A0B2DZJ4 | Microcin ABC transporter permease | yejB |
| A0A2J9KLC2 | Cytochrome C biogenesis protein CcsA | C2840_07610 |
| A0A2J9KLU0 | Aminopyrimidine aminohydrolase | tenA |
| A0A2J9KJI8 | Sialidase | C2840_02885 |
| A0A2J9KJW0 | Glycosyltransferase family 4 protein | C2840_02185 |
| A0A2J9KL32 | Lipopolysaccharide transport periplasmic protein LptA | lptA |
| A0A2J9KL32 | Lipopolysaccharide transport periplasmic protein LptA |  |
| A0A2J9KKM1 | L-lactate permease | C2840_00750 |
| A0A2J9KIG1 | Conjugal transfer protein TraG | C2840_05180 |
| O26021 | Conserved hypothetical integral membrane protein | HP_1487 |
| A0A2J9KL50 | DEAD/DEAH box helicase | C2840_07895 |
| A0A2J9KL60 | Cytochrome c oxidase accessory protein CcoG | ccoG |
| A0A2J9KL66 | Lipoprotein | C2840_08030 |
| A0A2J9KL77 | tRNA1(Val) (Adenine(37)-N6)-methyltransferase | C2840_07820 |
| A0A2J9KKT9 | Methyltransferase | C2840_00495 |
| A0A2J9KK63 | DUF2156 domain-containing protein | C2840_01510 |
| A0A2J9KL48 | Uncharacterized protein | C2840_08135 |
| A0A2J9KIQ3 | Tat pathway signal protein | C2840_04460 |
| A0A0M8NKA4 | Glycoside hydrolase family 43 | AM496_02115 |
| A0A2J9KKZ0 | Mannose-1-phosphate guanylyltransferase | C2840_00235 |
| A0A2J9KLS9 | Biotin synthase | C2840_06515 |
| A0A2J9KJP8 | Restriction endonuclease | C2840_02525 |
| A0A024C162 | Ribosomal silencing factor RsfS | rsfS |
| A0A2J9KKU5 | Conjugal transfer protein TrbL | C2840_00210 |
| A0A2J9KL18 | Uncharacterized protein | C2840_08150 |
| A0A3S6H258 | Heat shock protein HspR | hspR |
| A0A2J9KJR2 | Site-specific DNA-methyltransferase (adenine-specific) | C2840_02495 |
| A0A2J9KHQ0 | S41 family peptidase | C2840_07015 |
| A0A2J9KJC9 | DEAD/DEAH box helicase | C2840_03080 |
| A0A2J9KKB7 | Protein disulfide-isomerase | C2840_01205 |
| O25226 | Adenine specific DNA methyltransferase (MFOKI) | HP_0481 |
| O25519 | Type I restriction enzyme S protein (HsdS) | HP_0848 |
| A0A2J9KI67 | Glycosyltransferase family 8 protein | C2840_05700 |
| A0A2J9KII3 | 3-deoxy-D-manno-octulosonic acid transferase | C2840_04920 |
| A0A0B2E5U0 | Flagellar motor switch protein FliM | fliM |
| A0A2J9KI51 | OmpA family protein | C2840_05820 |
| A0A2J9KLC5 | Signal peptide peptidase SppA | sppA |
| A0A2J9KJT1 | Site-specific DNA-methyltransferase (adenine-specific) | C2840_02410 |
| A0A2J9KLG6 | Type I restriction enzyme R Protein | C2840_07285 |
| A0A2J9KJH4 | Ribose 5-phosphate isomerase B | rpiB |
| A0A2J9KK74 | DUF4149 domain-containing protein | C2840_01490 |
| A0A1A9H0F7 | Biotin carboxyl carrier protein of acetyl-CoA carboxylase | accB |
| A0A083YDP5 | Flagellar biosynthetic protein FliR | fliR |
| A0A2J9KLN9 | Nicotinamide riboside transporter PnuC | C2840_06700 |
| A0A2J9KL70 | Sodium/glutamate symporter | gltS |
| A0A0B2DRT0 | Pyruvate ferredoxin oxidoreductase | porC |
| A0A083YG54 | Cag pathogenicity island protein | cagU |
| A0A2J9KL75 | Dihydroneopterin aldolase | C2840_07850 |
| A0A2J9KK68 | Alginate_lyase domain-containing protein | C2840_01570 |
| A0A2J9KLK4 | Uncharacterized protein | C2840_06895 |
| A0A2J9KI68 | 2-dehydro-3-deoxy-phosphogluconate aldolase | C2840_05670 |
| A0A3S6H3S6 | Flagellar secretion chaperone FliS | fliS |
| A0A2J9KL80 | ABC transporter permease | C2840_07735 |
| A0A2J9KJV8 | VirB4 family type IV secretion/conjugal transfer ATPase | C2840_02280 |
| A0A2J9KM81 | Prephenate dehydrogenase | C2840_07175 |
| A0A2J9KIN5 | Flagellar basal body protein | flgE |
| A0A2A6W9Q8 | Uncharacterized protein | B0X51_04805 |
| A0A1Q2PLL9 | ABC transporter | B0X31_01790 |
| A0A024C0W8 | Cation:proton antiporter | AC782_02380 |
| A0A2J9KL25 | Penicillin-binding protein 2 | mrdA |
| A0A2J9KM03 | Modification methylase | C2840_06275 |
| A0A2J9KJM8 | VirB8 family protein | C2840_02755 |
| A0A060CZ58 | VirB3 type IV secretion protein | virB3 |
| J9RVI3 | Phospholipase A1 | pldA |
| A0A2I8V5P3 | ABC transporter permease | C2840_01305 |
| A0A2J9KJM6 | Cag pathogenicity island protein Cag1 | C2840_02705 |
| A0A2J9KLJ4 | Methyltransferase | C2840_07105 |
| A0A2J9KL61 | Site-specific DNA-methyltransferase (adenine-specific) | C2840_07880 |
| A0A2J9KKP5 | Phospho-2-dehydro-3-deoxyheptonate aldolase | C2840_00720 |
| A0A2J9KI07 | Amino acid ABC transporter permease | C2840_06050 |
| A0A2J9KJB1 | Nickel-dependent hydrogenase large subunit | C2840_03280 |
| A0A2J9KK10 | Uncharacterized protein | C2840_01980 |
| A0A2J9KKI8 | Glycosyltransferase family 8 protein | C2840_00845 |
| A0A2J9KJ72 | Cyclic dehypoxanthine futalosine synthase | mqnC |
| A0A2J9KIZ3 | Chorismate dehydratase | mqnA |
| A0A2I8UZ11 | Acetone carboxylase subunit alpha | C2840_03580 |
| A0A2J9KIS6 | Osmoprotection protein | C2840_04175 |
| A0A2J9KK25 | Single-stranded-DNA-specific exonuclease RecJ | recJ |
| A0A2J9KLQ6 | Phosphomannomutase/phosphoglucomutase | C2840_06620 |
| A0A2J9KKQ1 | Methyl-accepting chemotaxis protein | C2840_00550 |
| A0A2J9KKA1 | Chorismate mutase | C2840_01505 |
| A0A2J9KIH7 | RIP metalloprotease RseP | C2840_05040 |
| A0A2J9KJX8 | Class I SAM-dependent methyltransferase | C2840_02160 |
| A0A2J9KK9 | Bifunctional aminodeoxychorismate synthase component I/aminotransferase |  |
| A0A2J9KJW4 | Aminotransferase class V-fold PLP-dependent enzyme | C2840_02090 |
| A0A2J9KJE7 | Site-specific DNA-methyltransferase (adenine-specific) | C2840_03085 |
| A0A2J9KKJ9 | Uncharacterized protein | C2840_00795 |
| A0A2J9KJ53 | Uncharacterized protein | C2840_03630 |
| A0A2J9KLV9 | RDD family protein | C2840_06535 |
| A0A2J9KK28 | DUF1294 domain-containing protein | C2840_01775 |
| Q7BGI8 | NAD(P)H nitroreductase | frxA |
| O25168 | Uncharacterized protein | HP_0412 |
| A0A2J9KM64 | Uncharacterized protein | C2840_07455 |
| A0A2J9KKJ6 | Peptidase | C2840_00910 |
| A0A2J9KKM2 | Uncharacterized protein | C2840_00800 |
| A0A2J9KI82 | Exonuclease | C2840_05615 |
| O25810 | Ulcer-associated gene restriction endonuclease (IceA) | HP_1209 |
| A0A2J9KLH8 | Uncharacterized protein | C2840_07225 |
| A0A2J9KII8 | Toxin | C2840_04720 |
| O25380 | Site-specific DNA-methyltransferase (adenine-specific) | HP_0669 |
| O25908 | Putative adenine specific DNA methyltransferase | HP_1354 |
| A0A2J9KIN6 | Uncharacterized protein | C2840_04485 |
| A0A2J9KKD6 | CopG family transcriptional regulator | C2840_01160 |
| O25433 | G domain-containing protein | HP_0733 |
| A0A3S6H3C8 | Outer membrane efflux protein | HPYLPMSS1_00812 |
| A0A2J9KJZ0 | SPOR domain-containing protein | C2840_01985 |
| A0A2J9KLQ3 | 5'-nucleotidase, lipoprotein e(P4) family | C2840_06675 |
| A0A2J9KIL9 | Uncharacterized protein | C2840_04615 |
| A0A2J9KJB2 | TPR_REGION domain-containing protein | C2840_03420 |
| A0A2J9KJ25 | 5-formyltetrahydrofolate cyclo-ligase | C2840_03855 |
| A0A2J9KLQ8 | Lipopolysaccharide heptosyltransferase family protein | C2840_06670 |
| A0A2J9KJI6 | Site-2 protease family protein | C2840_02990 |
| A0A2J9KJF0 | Disulfide bond formation protein B | C2840_03095 |
| A0A2J9KJ95 | Uncharacterized protein | C2840_03505 |
| A0A2J9KLI3 | Methyltransferase | C2840_07110 |
| A0A2I8V959 | SH3 domain-containing protein | C2840_07010 |
| A0A2J9KJ40 | Outer membrane beta-barrel protein | C2840_03710 |
| O25969 | Uncharacterized protein | HP_1426 |
| A0A2J9KLR2 | Pantothenate kinase | C2840_06690 |
| A0A2J9KJD6 | ABC transporter substrate-binding protein | C2840_03175 |
| A0A2J9KKV3 | DNA translocase FtsK | C2840_00350 |
| A0A2J9KKL1 | Uncharacterized protein | C2840_00805 |
| A0A2J9KK96 | Uncharacterized protein | C2840_01415 |
| A0A2J9KIW3 | DUF3943 domain-containing protein | C2840_04100 |
| A0A2J9KII7 | DUF1049 domain-containing protein | C2840_04875 |
| A0A2J9KKV6 | DUF1523 domain-containing protein | C2840_00165 |
| A0A2J9KK46 | Uncharacterized protein | C2840_01785 |
| A0A246LRK4 | DUF2393 domain-containing protein | BGL70_06430 |
| A0A2J9KJE6 | Methyl-accepting chemotaxis protein | C2840_03120 |
| A0A2J9KIF2 | GTPase | C2840_04965 |
| A0A2J9KLA3 | Anaphase-promoting protein | C2840_07700 |
| A0A060CZR8 | VirB3 type IV secretion protein | virB3 |
| J0DPJ6 | LPS-assembly protein LptD | lptD |
| O25379 | Uncharacterized protein | HP_0668 |

**Table S3: List of essential non-homologous hypothetical proteins (78 proteins) screened using BLASTp tool.**

| **UniProt ID** | **Protein Name** | **Gene** |
| --- | --- | --- |
| A0A2J9KJE1 | Uncharacterized protein | C2840_03030 |
| A0A2J9KL74 | Carbon-nitrogen hydrolase family protein | C2840_07710 |
| A0A0M9W8H0 | Energy transducer TonB | AM498_03975 |
| A0A024C6E6 | Integral membrane protein | AC785_06050 |
| A0A2J9KJ54 | ATP-binding protein | C2840_03725 |
| A0A0B2EVL1 | Membrane protein | AC785_07295 |
| A0A2J9KL87 | Histidine kinase | C2840_07770 |
| A0A0B2ED21 | Uncharacterized protein | ACM24_04610 |
| A0A2J9KLR0 | NADH-quinone oxidoreductase subunit G | C2840_06575 |
| A0A2J9KJD7 | Penicillin-insensitive transglycosylase | C2840_03110 |
| A0A2J9KIE6 | Amino acid permease | C2840_05250 |
| A0A2J9KKP0 | CcoQ/FixQ family Cbb3-type cytochrome c oxidase assembly chaperone | C2840_00780 |
| O25063 | Toxin-like outer membrane protein | HP_0289 |
| A0A2J9KJF6 | 2-oxoglutarate synthase subunit alpha | C2840_03065 |
| A0A2J9KJ11 | ABC transporter permease | C2840_03830 |
| A0A2J9KJ42 | t(6)A37 threonylcarbamoyladenosine biosynthesis protein TsaE | C2840_03665 |
| A0A2J9KK87 | Lipopolysaccharide heptosyltransferase I | waaC |
| A0A2J9KJR0 | Oligoendopeptidase F | C2840_02445 |
| A0A083YCR3 | Membrane protein | B0X28_02655 |
| A0A2J9KL17 | DNA helicase | C2840_08065 |
| A0A2J9KKD0 | YbhB/YbcL family Raf kinase inhibitor-like protein | C2840_01140 |
| A0A2J9KK15 | Cytochrome C biogenesis protein | C2840_01955 |
| A0A2J9KLT5 | Uncharacterized protein | C2840_06695 |
| A0A2J9KLY0 | RDD family protein | C2840_06325 |
| A0A2J9KLG9 | Uncharacterized protein | C2840_07470 |
| A0A2J9KIG5 | ATPase | C2840_04950 |
| O25815 | LPS-assembly protein LptD | lptD |
| A0A293TFY6 | Uncharacterized protein | BB469_02105 |
| A0A2J9KJI0 | Uncharacterized protein | C2840_03050 |
| A0A2J9KHY0 | KH domain-containing protein | C2840_05950 |
| A0A2J9KJA3 | UTP--glucose-1-phosphate uridylyltransferase | galU |
| A0A2J9KI79 | TlyA family rRNA (Cytidine-2'-O)-methyltransferase | C2840_05600 |
| A0A2J9KJ30 | UDP-N-acetylmuramoyl-tripeptide--D-alanyl-D-alanine ligase | C2840_03775 |
| A0A0B2E3F3 | 2-nitropropane dioxygenase | AM497_02530 |
| A0A2J9KLX5 | DNA polymerase III subunit delta | C2840_06395 |
| A0A2J9KK79 | Indole-3-glycerol-phosphate synthase | C2840_01430 |
| A0A2J9KJW2 | VirB8 family protein | C2840_02270 |
| A0A2J9KI37 | Riboflavin biosynthesis protein | C2840_05605 |
| A0A2J9KI28 | ATP F0F1 synthase subunit B | B0X61_02885 |
| A0A2J9KK92 | Cytochrome C biogenesis protein | C2840_01375 |
| A0A2J9KK66 | ABC transporter permease | C2840_01550 |
| A0A2J9KJE8 | Endolytic murein transglycosylase | mltG |
| O25487 | Iron(III) dicitrate transport protein (FecA) | HP_0807 |
| A0A2J9KJG3 | 2-oxoglutarate:acceptor oxidoreductase | oorC |
| A0A3S6H489 | 2-oxoglutarate oxidoreductase subunit KorB | korB |
| A0A2J9KI75 | Prokaryotic metallothionein family protein | C2840_05485 |
| A0A2J9KJ51 | Phosphatidylglycerophosphatase A | C2840_03760 |
| A0A2J9KK64 | ABC transporter substrate-binding protein | C2840_01540 |
| A0A2J9KJB3 | YggT family protein | C2840_03340 |
| A0A2J9KI11 | ATP synthase F(1) sector subunit delta | C2840_05865 |
| A0A2J9KLB0 | ComF family protein | C2840_07670 |
| O25980 | Uncharacterized protein | HP_1439 |
| A0A2J9KL15 | PAP2 family protein | C2840_08205 |
| A0A2J9KLP4 | ATP-binding protein | C2840_06860 |
| A0A2J9KJI8 | Sialidase | C2840_02885 |
| A0A2J9KL66 | Lipoprotein | C2840_08030 |
| A0A2J9KL77 | tRNA1(Val) (Adenine(37)-N6)-methyltransferase | C2840_07820 |
| A0A2J9KKT9 | Methyltransferase | C2840_00495 |
| A0A2J9KL48 | Uncharacterized protein | C2840_08135 |
| A0A2J9KLS9 | Biotin synthase | C2840_06515 |
| A0A024C162 | Ribosomal silencing factor RsfS | rsfS |
| A0A2J9KL18 | Uncharacterized protein | C2840_08150 |
| A0A2J9KII3 | 3-deoxy-D-manno-octulosonic acid transferase | C2840_04920 |
| A0A2J9KJH4 | Ribose 5-phosphate isomerase B | rpiB |
| A0A2J9KK74 | DUF4149 domain-containing protein | C2840_01490 |
| A0A0B2DRT0 | Pyruvate ferredoxin oxidoreductase | porC |
| A0A2J9KL75 | Dihydroneopterin aldolase | C2840_07850 |
| A0A2J9KI07 | Amino acid ABC transporter permease | C2840_06050 |
| A0A2J9KJ72 | Cyclic dehypoxanthine futalosine synthase | mqnC |
| A0A2J9KK9 | Bifunctional aminodeoxychorismate synthase component I/aminotransferase |  |
| A0A2J9KJW4 | Aminotransferase class V-fold PLP-dependent enzyme | C2840_02090 |
| A0A2J9KJ53 | Uncharacterized protein | C2840_03630 |
| A0A2J9KIN6 | Uncharacterized protein | C2840_04485 |
| A0A2J9KKD6 | CopG family transcriptional regulator | C2840_01160 |
| A0A3S6H3C8 | Outer membrane efflux protein | HPYLPMSS1_00812 |
| A0A2I8V959 | SH3 domain-containing protein | C2840_07010 |
| A0A2J9KKV6 | DUF1523 domain-containing protein | C2840_00165 |
| J0DPJ6 | LPS-assembly protein LptD | lptD |

**Table S4: List of unique metabolic pathways of the *H. pylori*.**

| **Entry** | **Name** | **Description** |
| --- | --- | --- |
| hpy01110 | Biosynthesis of secondary metabolites | NA |
| hpy01120 | Microbial metabolism in diverse environments | NA |
| hpy00460 | Cyanoamino acid metabolism | NA |
| hpy00680 | Methane metabolism | Methane is metabolized principally by methanotrophs and methanogens in the global carbon cycle. Meth... |
| hpy00300 | Lysine biosynthesis | NA |
| hpy00362 | Benzoate degradation | NA |
| hpy02020 | Two-component system | Two-component signal transduction systems enable bacteria to sense, respond, and adapt to changes in... |
| hpy00473 | D-Alanine metabolism | NA |
| hpy00643 | Styrene degradation | NA |
| hpy00261 | Monobactam biosynthesis | Monobactams are beta-lactam antibiotics containing a monocyclic beta-lactam nucleus, which is struct... |
| hpy00627 | Aminobenzoate degradation | NA |
| hpy01502 | Vancomycin resistance | Vancomycin (VCM) is a glycopeptide antibiotic agent that inhibits the synthesis of peptidolgycan in ... |
| hpy01503 | Cationic antimicrobial peptide (CAMP) resistance | Cationic antimicrobial peptides (CAMPs) play an important role in host defense against microbial inf... |
| hpy00401 | Novobiocin biosynthesis | NA |
| hpy00540 | Lipopolysaccharide biosynthesis | Lipopolysaccharide (LPS) is the major component of the outer membrane of Gram-negative bacteria cons... |
| hpy00550 | Peptidoglycan biosynthesis | Peptidoglycan is a macromolecule made of long aminosugar strands cross-linked by short peptides. It ... |
| hpy01501 | beta-Lactam resistance | The beta-lactam antibiotics are the most widely used group of antibiotics, which exert their effect ... |
| hpy02024 | Quorum sensing | Quorum sensing (QS) is a regulatory system that allows bacteria to share information about cell dens... |
| hpy02040 | Flagellar assembly | NA |
| hpy00541 | O-Antigen nucleotide sugar biosynthesis | The O-antigen is a repetitive glycan polymer with a repeat unit of two to six sugar residues, and co... |
| hpy02030 | Bacterial chemotaxis | Chemotaxis is the process by which cells sense chemical gradients in their environment and then move... |
| hpy00121 | Secondary bile acid biosynthesis | The secondary bile acids are derived from the primary bile acids by the enzymatic action of intestin... |
| hpy00521 | Streptomycin biosynthesis | Streptomycin is an aminocyclitol-aminoglycoside antibiotic produced by Streptomyces griseus. Strepto... |
| hpy01220 | Degradation of aromatic compounds | Microorganisms are known to be capable of degrading diverse chemical substances including man-made c... |
| hpy03070 | Bacterial secretion system | Gram-negative bacteria secrete a wide range of proteins whose functions include biogenesis of organe... |

**NA: Not Available*

**Table S5: List of proteins with KO number assigned**

| **Protein Accession number (Uniprot)** |
| --- |
| tr\|A0A2J9KJE1\|A0A2J9KJE1_HELPX |
| tr\|A0A2J9KL74\|A0A2J9KL74_HELPX |
| tr\|A0A0M9W8H0\|A0A0M9W8H0_HELPX |
| tr\|A0A024C6E6\|A0A024C6E6_HELPX |
| tr\|A0A2J9KJ54\|A0A2J9KJ54_HELPX |
| tr\|A0A0B2EVL1\|A0A0B2EVL1_HELPX |
| tr\|A0A2J9KL87\|A0A2J9KL87_HELPX |
| tr\|A0A0B2ED21\|A0A0B2ED21_HELPX |
| tr\|A0A2J9KLR0\|A0A2J9KLR0_HELPX K00336 |
| tr\|A0A2J9KJD7\|A0A2J9KJD7_HELPX K05366 |
| tr\|A0A2J9KIE6\|A0A2J9KIE6_HELPX K03293 |
| tr\|A0A2J9KKP0\|A0A2J9KKP0_HELPX K00407 |
| tr\|O25063\|O25063_HELPY |
| tr\|A0A2J9KJW2\|A0A2J9KJW2_HELPX K03203 |
| tr\|A0A2J9KI37\|A0A2J9KI37_HELPX K11753 |
| tr\|A0A2J9KI28\|A0A2J9KI28_HELPX K02109 |
| tr\|A0A2J9KK92\|A0A2J9KK92_HELPX K06196 |
| tr\|A0A2J9KK66\|A0A2J9KK66_HELPX K12370 |
| tr\|A0A2J9KJE8\|A0A2J9KJE8_HELPX K07082 |
| tr\|O25487\|O25487_HELPY K16091 |
| tr\|A0A2J9KJG3\|A0A2J9KJG3_HELPX K00177 |
| tr\|A0A3S6H489\|A0A3S6H489_HELPX K00175 |
| tr\|A0A2J9KI75\|A0A2J9KI75_HELPX K06950 |
| tr\|A0A2J9KJ51\|A0A2J9KJ51_HELPX K01095 |
| tr\|A0A2J9KK64\|A0A2J9KK64_HELPX K12368 |
| tr\|A0A2J9KJB3\|A0A2J9KJB3_HELPX K02221 |
| tr\|A0A2J9KI11\|A0A2J9KI11_HELPX K02113 |
| tr\|A0A2J9KLB0\|A0A2J9KLB0_HELPX K02242 |
| tr\|O25980\|O25980_HELPY |
| tr\|A0A2J9KL15\|A0A2J9KL15_HELPX K23159 |
| tr\|A0A2J9KLP4\|A0A2J9KLP4_HELPX K07133 |
| tr\|A0A2J9KJI8\|A0A2J9KJI8_HELPX |
| tr\|A0A2J9KL66\|A0A2J9KL66_HELPX |
| tr\|A0A2J9KL77\|A0A2J9KL77_HELPX |
| tr\|A0A2J9KKT9\|A0A2J9KKT9_HELPX K00571 |
| tr\|A0A2J9KL48\|A0A2J9KL48_HELPX |
| tr\|A0A2J9KLS9\|A0A2J9KLS9_HELPX K02169 |
| tr\|A0A024C162\|A0A024C162_HELPX K09710 |
| tr\|A0A2J9KL18\|A0A2J9KL18_HELPX |
| tr\|A0A2J9KII3\|A0A2J9KII3_HELPX K02527 |
| tr\|A0A2J9KJH4\|A0A2J9KJH4_HELPX K01808 |
| tr\|A0A2J9KK74\|A0A2J9KK74_HELPX |
| tr\|A0A0B2DRT0\|A0A0B2DRT0_HELPX K00172 |
| tr\|A0A2J9KL75\|A0A2J9KL75_HELPX K01633 |
| tr\|A0A2J9KI07\|A0A2J9KI07_HELPX K02029 |
| tr\|A0A2J9KJ72\|A0A2J9KJ72_HELPX K11784 |
| tr\|A0A2J9KJW4\|A0A2J9KJW4_HELPX |
| tr\|A0A2J9KJ53\|A0A2J9KJ53_HELPX |
| tr\|A0A2J9KIN6\|A0A2J9KIN6_HELPX |
| tr\|A0A2J9KKD6\|A0A2J9KKD6_HELPX |
| tr\|A0A3S6H3C8\|A0A3S6H3C8_HELPX |
| tr\|A0A2I8V959\|A0A2I8V959_HELPX |
| tr\|A0A2J9KKV6\|A0A2J9KKV6_HELPX |
| tr\|J0DPJ6\|J0DPJ6_HELPX |

**Table S6: The novel the membrane proteins as a drug target was analyzed using the Drug Bank database**

| **Uniprot ID** | **Name** | **Gene** | **Drug Bank ID** | **Drug** | **Drug Group** | **E-value** |
| --- | --- | --- | --- | --- | --- | --- |
| A0A2J9KLR0 | NADH-quinone oxidoreductase subunit G | NDUFS1 | DB00157 | NADH | approved, nutraceutical | 1.94E-27 |
| A0A2J9KJD7 | Penicillin binding protein | PBP1 | DB01060 | Amoxicillin | approved, vet_approved | 0 |
|  | Penicillin-binding protein 1B | ponB | DB01329 | Cefoperazone | Approved, Investigational | 4.22E-82 |
|  |  |  | DB00430 | Cefpiramide | Approved |  |
|  |  |  | DB00438 | Ceftazidime | Approved |  |
|  |  |  | DB09050 | Ceftolozane | Approved, Investigational | |
|  |  |  | DB14879 | Cefiderocol | Approved, Investigational | |
|  | Penicillin-binding protein 1B | mrcB | DB01598 | Imipenem | approved | 4.21E-69 |
|  |  |  | DB01329 | Cefoperazone | Approved, Investigational | |
|  |  |  | DB01332 | Ceftizoxime | Approved, Investigational | |
|  |  |  | DB01327 | Cefradine | approved |  |
|  |  |  | DB01331 | Cefazolin | approved |  |
|  |  |  | DB01328 | Cefoxitin | Approved, Investigational | |
|  |  |  | DB01415 | Cefonicid | Approved, Investigational | |
|  |  |  | DB00430 | Ceftibuten | approved |  |
|  |  |  | DB00438 | Cefpiramide | approved |  |
|  |  |  | DB00274 | Ceftazidime | Approved, Investigational | |
|  |  |  | DB00303 | Ertapenem | approved, investigational | |
|  |  |  | DB00689 | **Cephaloglycin** | Approved |  |
|  |  |  | DB01414 | Cefacetrile | experimental, vet_approved | |
|  |  |  | DB04570 | **Latamoxef** | Approved, Investigational | |
|  |  |  | DB06211 | Doripenem | Approved, Investigational | |
|  |  |  | DB11367 | Cefroxadine | withdrawn |  |
|  |  |  | DB00578 | Carbenicillin | Approved, Investigational | |
|  |  |  | DB09319 | Carindacillin | Approved, Investigational | |
|  |  |  | DB09050 | Ceftolozane | Approved, Investigational | |
|  |  |  | DB01602 | Bacampicillin | Approved, Investigational | |
|  |  |  | DB01000 | Cyclacillin | approved |  |
|  | Penicillin-binding protein 1A | pbpA | DB00229 | Cefotiam | approved, investigational | 3.55E-66 |
|  |  |  | DB00267 | Cefmenoxime | approved |  |
|  |  |  | DB00301 | Flucloxacillin | approved, investigational | |
|  |  |  | DB00417 | Phenoxymethylpenicillin | approved, vet_approved | |
|  |  |  | DB00447 | Loracarbef | approved, investigational, withdrawn | |
|  |  |  | DB00456 | Cefalotin | approved, investigational, vet_approved | |
|  |  |  | DB00713 | Oxacillin | approved, investigational | |
|  |  |  | DB00833 | Cefaclor | approved |  |
|  |  |  | DB00948 | Mezlocillin | approved, investigational | |
|  |  |  | DB01061 | Azlocillin | approved |  |
|  |  |  | DB01066 | Cefditoren | approved, investigational | |
|  |  |  | DB01112 | Cefuroxime | approved |  |
|  |  |  | DB01139 | Cefapirin | approved, vet_approved | |
|  |  |  | DB01147 | Cloxacillin | approved, investigational, vet_approved | |
|  |  |  | DB01604 | Pivampicillin | approved |  |
|  |  |  | DB01605 | Pivmecillinam | approved |  |
|  |  |  | DB04133 | Degraded Cephaloridine | experimental | |
|  | Penicillin-binding protein 1A | ponA | DB05659 | Faropenem medoxomil | investigational | 1.96E-59 |
|  |  |  | DB01150 | Cefprozil | approved |  |
|  | Penicillin-binding protein 1A | pbpA | DB08375 | (2R)-2-[(1R)-1-[[(2Z)-2-(2-Amino-1,3-thiazol-4-yl)-2-methoxyiminoacetyl]amino]-2-oxoethyl]-5-methylidene-2H-1,3-thiazine-4-carboxylic acid | experimental | 3.70E-58 |
|  |  |  | DB01140 | Cefadroxil | approved, vet_approved, withdrawn | |
|  |  |  | DB00493 | Cefotaxime | approved |  |
|  |  |  | DB00607 | Nafcillin | approved, investigational | |
|  |  |  | DB00415 | Ampicillin | approved, vet_approved | |
|  |  |  | DB00485 | Dicloxacillin | approved, investigational, vet_approved | |
|  |  |  | DB01163 | Amdinocillin | investigational, withdrawn | |
|  |  |  | DB01603 | Meticillin | approved, investigational |  |
|  |  |  | DB00456 | Cefalotin | approved, investigational, vet_approved | |
|  |  |  | DB00713 | Oxacillin | approved, investigational | |
|  |  |  | DB01331 | Cefoxitin | approved |  |
|  |  |  | DB00567 | Cephalexin | approved, investigational, vet_approved | |
|  |  |  | DB03313 | Cephalosporin C | experimental | |
|  |  |  | DB00438 | Ceftazidime | approved |  |
|  |  |  | DB08795 | Azidocillin | experimental | |
|  |  |  | DB00739 | Hetacillin | approved, vet_approved, withdrawn | |
|  |  |  | DB01000 | Cyclacillin | approved |  |
|  | Penicillin-binding protein 1C | pbpC | DB01327 | Cefazolin | approved | 3.38E-52 |
|  |  |  | DB09050 | Ceftolozane | approved, investigational | |
|  | Penicillin-binding protein 2a | pbp2a | DB00493 | Cefotaxime | approved | 2.21E-48 |
|  |  |  | DB00319 | Piperacillin | approved |  |
|  |  |  | DB00607 | Nafcillin | approved, investigational | |
|  |  |  | DB00415 | Ampicillin | approved, vet_approved | |
|  |  |  | DB00485 | Dicloxacillin | approved, investigational, vet_approved | |
|  |  |  | DB01163 | Amdinocillin | investigational, withdrawn | |
|  |  |  | DB01603 | Meticillin | approved, investigational | |
|  |  |  | DB00456 | Cefalotin | approved, investigational, vet_approved | |
|  |  |  | DB00713 | Oxacillin | approved, investigational | |
|  |  |  | DB01331 | Cefoxitin | approved |  |
|  |  |  | DB00567 | Cephalexin | approved, investigational, vet_approved | |
|  |  |  | DB03313 | Cephalosporin C | experimental | |
|  |  |  | DB08795 | Azidocillin | experimental | |
|  |  |  | DB01147 | Cloxacillin | approved, investigational, vet_approved | |
|  |  |  | DB00739 | Hetacillin | approved, vet_approved, withdrawn | |
|  | Penicillin-binding protein 1A | mrcA |  |  |  | 2.18E-46 |
|  | Penicillin-binding protein 2 | pbp2 | DB04147 | Dodecyldimethylamine N-oxide | experimental | 4.17E-43 |
|  | Penicillin-binding protein 1b | pbp1b |  |  |  | 1.69E-36 |
|  | Penicillin-binding protein 1B | pbp1b | DB08401 | (2e)-2-({(2s)-2-carboxy-2-[(phenoxyacetyl)amino] ethoxy}imino)pentanedioic acid | experimental | 1.69E-36 |
|  | Penicillin-binding protein 2 | penA | DB00535 | Cefdinir | approved | 8.75E-13 |
|  | Cell division protein | pbpB | DB01413 | Cefepime | approved, investigational | 1.51E-11 |
|  |  |  | DB01147 | Cloxacillin | approved, investigational, vet_approved | |
|  |  |  | DB01147 | Ceftolozane | approved, investigational | |
|  |  |  | DB06211 | Doripenem | approved, investigational | |
|  |  |  | DB14879 | Cefiderocol | approved, investigational | |
|  | Peptidoglycan D,D-transpeptidase FtsI | ftsI | DB05659 | Faropenem medoxomil | investigational | 4.78E-08 |
|  | Peptidoglycan synthase FtsI | ftsI | DB04918 | Ceftobiprole | approved, investigational | 4.78E-08 |
|  |  |  | DB00267 | Cefmenoxime | approved |  |
|  |  |  | DB01416 | Cefpodoxime | approved, vet_approved | |
|  |  |  | DB01329 | Cefoperazone | approved, investigational | |
|  |  |  | DB01327 | Cefazolin | approved |  |
|  |  |  | DB01331 | Cefoxitin | approved |  |
|  |  |  | DB01328 | Cefonicid | approved, investigational | |
|  |  |  | DB01413 | Cefepime | approved, investigational | |
|  |  |  | DB01415 | Ceftibuten | approved, investigational | |
|  |  |  | DB00430 | Cefpiramide | approved |  |
|  |  |  | DB00438 | Ceftazidime | approved |  |
|  |  |  | DB00274 | Cefmetazole | approved, investigational | |
|  |  |  | DB00303 | Ertapenem | approved, investigational | |
|  |  |  | DB00578 | Carbenicillin | approved, investigational | |
|  |  |  | DB09319 | Carindacillin | approved, investigational | |
|  |  |  | DB09050 | Ceftolozane | approved, investigational | |
|  |  |  | DB01602 | Bacampicillin | approved, investigational | |
|  |  |  | DB01000 | Cyclacillin | approved |  |
|  | Penicillin-binding protein 3 | pbpC | DB00355 | Aztreonam | approved | 2.73E-07 |
|  |  |  | DB00493 | Cefotaxime | approved |  |
|  |  |  | DB01598 | Imipenem | approved |  |
|  |  |  | DB04570 | Latamoxef | approved, investigational | |
| A0A2J9KI37 | Riboflavin biosynthesis protein | NA | DB03247 | Flavin mononucleotide | approved, investigational | 1.78E-18 |
|  |  |  | DB04272 | Citric acid | approved, nutraceutical, vet_approved | |
|  |  |  | DB04345 | Lumichrome | experimental | |
| O25487 | Fe(3+) dicitrate transport protein FecA | fecA | DB04079 | Heptane-1,2,3-Triol | experimental | 1.46E-41 |
|  |  |  | DB04147 | Dodecyldimethylamine N-oxide | experimental | |
|  | Colicin I receptor | cirA | DB04147 | Dodecyldimethylamine N-oxide | experimental | 1.23E-10 |
|  |  |  | DB02415 | N-Octyl-2-Hydroxyethyl Sulfoxide | experimental | |
|  |  |  | DB14879 | Cefiderocol | approved, investigational | |
|  | Vitamin B12 transporter BtuB | btuB | DB04233 | (Hydroxyethyloxy)Tri(Ethyloxy)Octane | experimental | 2.18E-09 |
|  |  |  | DB04147 | Dodecyldimethylamine N-oxide | experimental | |
|  |  |  | DB04039 | 3-Oxo-Pentadecanoic Acid | experimental | |
| A0A3S6H489 | Pyruvate synthase | por | DB01987 | Cocarboxylase | approved, experimental | 9.35E-08 |
|  |  |  | DB02410 | 2-Acetyl-3-[(4-Amino-2-Methyl-5-Pyrimidinyl)Methyl]-4-Methyl-5-(4,6,6-Trihydroxy-3,5-Dioxa-4,6-Diphosphahex-1-Yl)Thiazolium Inner Salt P,P'-Dioxide | experimental | |
|  |  |  | DB00507 | Nitazoxanide | approved, investigational, vet_approved | |
|  |  |  | DB00507 | Nitazoxanide | approved, investigational, vet_approved | |
| A0A2J9KK64 | Nickel-binding periplasmic protein | nikA | DB03374 | 3,5-Diiodotyrosine | experimental | 6.05E-35 |
|  | Periplasmic oligopeptide-binding protein | oppA | DB07365 | 1-Naphthyl-L-alanine | experimental | 2.08E-29 |
|  | Mannoside ABC transport system, sugar-binding protein | NA | DB01942 | Formic acid | experimental, investigational | 3.06E-15 |
| A0A2J9KKT9 | Modification methylase RsrI | rsrIM | DB01752 | S-adenosyl-L-homocysteine | experimental | 4.58E-07 |
|  |  |  | DB01910 | Sinefungin | experimental | |
|  |  |  | DB02282 | 5'-S-methyl-5'-thioadenosine | experimental | |
| A0A2J9KJH4 | Ribose 5-phosphate isomerase B | NA | DB03661 | L-cysteic acid | experimental | 1.86E-41 |
|  | Ribose-5-phosphate isomerase B | rpiB | DB03108 | 4-phospho-D-erythronic acid | experimental | 2.15E-15 |
|  |  |  | DB04496 | 4-Phospho-D-erythronohydroxamic acid | experimental | |
| A0A2J9KJW4 | Cysteine desulfurase | sufS | DB02345 | Selenocysteine | experimental | 5.40E-18 |
|  |  |  | DB02761 | S-Mercaptocysteine | experimental | |
|  |  |  | DB03049 | S-Selanyl Cysteine | experimental | |
|  |  |  | DB04217 | L-2-amino-3-butynoic acid | experimental | |
|  | Probable cysteine desulfurase | csd | DB02346 | 3'-O-N-Octanoyl-a-D-Glucopyranosyl-B-D-Fructofuranoside | experimental | 1.28E-15 |

**Table** **S7: Conservancy analysis of *H. pylori* 26695 LpxF sequence with other strains using BLASTp**

| **Select for downloading or viewing reports** | Description | Scientific Name | Max Score | Total Score | Query Cover | E value | Per. Ident | Acc. Len | **Accession** |  |
| --- | --- | --- | --- | --- | --- | --- | --- | --- | --- | --- |
| 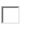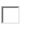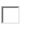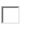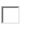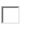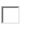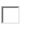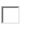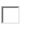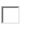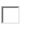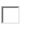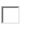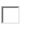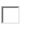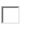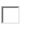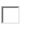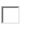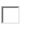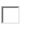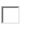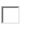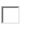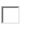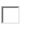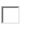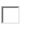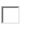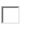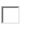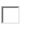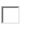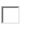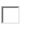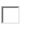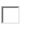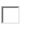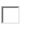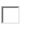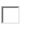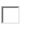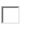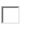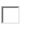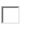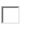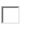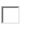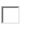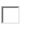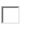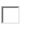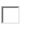   \| ref\|WP_000734125.1\| \| \| --- \| | lipid A 4'-phosphatase [Helicobacter pylori] | Helicobacter pylori | 402 | 402 | 100% | 3.00E-141 | 100.00% | 198 | WP_000734125.1 | |
| ref\|WP_064787658.1\| | lipid A 4'-phosphatase [Helicobacter pylori] | Helicobacter pylori | 398 | 398 | 100% | 6.00E-140 | 98.99% | 198 | WP_064787658.1 | |
| gb\|PUD41911.1\| | hypothetical protein C2R92_01920 [Helicobacter pylori] | Helicobacter pylori | 398 | 398 | 100% | 1.00E-139 | 98.48% | 220 | PUD41911.1 | |
| ref\|WP_001877740.1\| | lipid A 4'-phosphatase [Helicobacter pylori] | Helicobacter pylori | 397 | 397 | 100% | 1.00E-139 | 98.48% | 198 | WP_001877740.1 | |
| gb\|MUU54252.1\| | phosphatase PAP2 family protein [Helicobacter pylori] | Helicobacter pylori | 398 | 398 | 100% | 1.00E-139 | 98.48% | 220 | MUU54252.1 | |
| gb\|MUU49066.1\| | phosphatase PAP2 family protein [Helicobacter pylori] | Helicobacter pylori | 398 | 398 | 100% | 1.00E-139 | 98.48% | 220 | MUU49066.1 | |
| gb\|MUU44635.1\| | phosphatase PAP2 family protein [Helicobacter pylori] | Helicobacter pylori | 398 | 398 | 100% | 2.00E-139 | 98.48% | 220 | MUU44635.1 | |
| ref\|WP_164500185.1\| | lipid A 4'-phosphatase [Helicobacter pylori] | Helicobacter pylori | 397 | 397 | 100% | 2.00E-139 | 98.48% | 198 | WP_164500185.1 | |
| ref\|WP_000734132.1\| | lipid A 4'-phosphatase [Helicobacter pylori] | Helicobacter pylori | 397 | 397 | 100% | 2.00E-139 | 98.48% | 198 | WP_000734132.1 | |
| gb\|NHA57525.1\| | phosphatase PAP2 family protein [Helicobacter pylori] | Helicobacter pylori | 397 | 397 | 100% | 3.00E-139 | 97.98% | 220 | NHA57525.1 | |
| gb\|MUU75989.1\| | phosphatase PAP2 family protein [Helicobacter pylori] | Helicobacter pylori | 397 | 397 | 100% | 3.00E-139 | 97.98% | 220 | MUU75989.1 | |
| ref\|WP_156541729.1\| | lipid A 4'-phosphatase [Helicobacter pylori] | Helicobacter pylori | 396 | 396 | 100% | 3.00E-139 | 97.98% | 198 | WP_156541729.1 | |
| ref\|WP_108338003.1\| | lipid A 4'-phosphatase [Helicobacter pylori] | Helicobacter pylori | 396 | 396 | 100% | 3.00E-139 | 98.48% | 198 | WP_108338003.1 | |
| ref\|WP_120900291.1\| | lipid A 4'-phosphatase [Helicobacter pylori] | Helicobacter pylori | 396 | 396 | 100% | 4.00E-139 | 97.98% | 198 | WP_120900291.1 | |
| ref\|WP_001940762.1\| | lipid A 4'-phosphatase [Helicobacter pylori] | Helicobacter pylori | 396 | 396 | 100% | 4.00E-139 | 97.98% | 198 | WP_001940762.1 | |
| gb\|MUU22597.1\| | phosphatase PAP2 family protein [Helicobacter pylori] | Helicobacter pylori | 397 | 397 | 100% | 4.00E-139 | 97.98% | 220 | MUU22597.1 | |
| gb\|MUU26950.1\| | phosphatase PAP2 family protein [Helicobacter pylori] | Helicobacter pylori | 397 | 397 | 100% | 5.00E-139 | 97.98% | 220 | MUU26950.1 | |
| ref\|WP_000734126.1\| | lipid A 4'-phosphatase [Helicobacter pylori] | Helicobacter pylori | 395 | 395 | 100% | 5.00E-139 | 97.98% | 198 | WP_000734126.1 | |
| ref\|WP_164499577.1\| | lipid A 4'-phosphatase [Helicobacter pylori] | Helicobacter pylori | 395 | 395 | 100% | 6.00E-139 | 97.98% | 198 | WP_164499577.1 | |
| gb\|NHB16291.1\| | phosphatase PAP2 family protein [Helicobacter pylori] | Helicobacter pylori | 396 | 396 | 100% | 6.00E-139 | 97.98% | 220 | NHB16291.1 | |
| ref\|WP_001965779.1\| | lipid A 4'-phosphatase [Helicobacter pylori] | Helicobacter pylori | 395 | 395 | 100% | 7.00E-139 | 97.98% | 198 | WP_001965779.1 | |
| ref\|WP_078271457.1\| | lipid A 4'-phosphatase [Helicobacter pylori] | Helicobacter pylori | 395 | 395 | 100% | 8.00E-139 | 97.98% | 198 | WP_078271457.1 | |
| gb\|KAA6497660.1\| | phosphatase PAP2 family protein [Helicobacter pylori] | Helicobacter pylori | 396 | 396 | 100% | 8.00E-139 | 97.98% | 220 | KAA6497660.1 | |
| ref\|WP_001901772.1\| | lipid A 4'-phosphatase [Helicobacter pylori] | Helicobacter pylori | 395 | 395 | 100% | 9.00E-139 | 97.98% | 198 | WP_001901772.1 | |
| gb\|MUV08095.1\| | phosphatase PAP2 family protein [Helicobacter pylori] | Helicobacter pylori | 396 | 396 | 100% | 9.00E-139 | 97.98% | 220 | MUV08095.1 | |
| ref\|WP_000734133.1\| | lipid A 4'-phosphatase [Helicobacter pylori] | Helicobacter pylori | 395 | 395 | 100% | 9.00E-139 | 97.98% | 198 | WP_000734133.1 | |
| ref\|WP_000734379.1\| | lipid A 4'-phosphatase [Helicobacter pylori] | Helicobacter pylori | 395 | 395 | 100% | 9.00E-139 | 97.98% | 198 | WP_000734379.1 | |
| ref\|WP_000734374.1\| | lipid A 4'-phosphatase [Helicobacter pylori] | Helicobacter pylori | 395 | 395 | 100% | 9.00E-139 | 97.98% | 198 | WP_000734374.1 | |
| gb\|MUU32138.1\| | phosphatase PAP2 family protein [Helicobacter pylori] | Helicobacter pylori | 395 | 395 | 100% | 1.00E-138 | 97.98% | 220 | MUU32138.1 | |
| gb\|OOP93791.1\| | hypothetical protein B0X37_00445 [Helicobacter pylori] | Helicobacter pylori | 395 | 395 | 100% | 1.00E-138 | 97.47% | 220 | OOP93791.1 | |
| ref\|WP_001891965.1\| | lipid A 4'-phosphatase [Helicobacter pylori] | Helicobacter pylori | 394 | 394 | 100% | 2.00E-138 | 97.98% | 198 | WP_001891965.1 | |
| ref\|WP_172825141.1\| | lipid A 4'-phosphatase [Helicobacter pylori] | Helicobacter pylori | 394 | 394 | 100% | 2.00E-138 | 97.47% | 198 | WP_172825141.1 | |
| ref\|WP_162974695.1\| | lipid A 4'-phosphatase [Helicobacter pylori] | Helicobacter pylori | 394 | 394 | 100% | 2.00E-138 | 97.98% | 198 | WP_162974695.1 | |
| ref\|WP_001880993.1\| | lipid A 4'-phosphatase [Helicobacter pylori] | Helicobacter pylori | 394 | 394 | 100% | 2.00E-138 | 97.98% | 198 | WP_001880993.1 | |
| ref\|WP_201737469.1\| | phosphatase PAP2 family protein [Helicobacter pylori] | Helicobacter pylori | 394 | 394 | 100% | 2.00E-138 | 97.47% | 198 | WP_201737469.1 | |
| gb\|RKV03135.1\| | hypothetical protein DDP57_00090 [Helicobacter pylori] | Helicobacter pylori | 395 | 395 | 100% | 2.00E-138 | 97.47% | 220 | RKV03135.1 | |
| gb\|EMH28393.1\| | PAP2 family protein [Helicobacter pylori GAM268Bii] | Helicobacter pylori GAM268Bii | 395 | 395 | 100% | 2.00E-138 | 97.47% | 220 | EMH28393.1 | |
| gb\|RKV53740.1\| | hypothetical protein DD775_07950 [Helicobacter pylori] | Helicobacter pylori | 395 | 395 | 100% | 2.00E-138 | 97.47% | 220 | RKV53740.1 | |
| ref\|WP_140592929.1\| | lipid A 4'-phosphatase [Helicobacter pylori] | Helicobacter pylori | 394 | 394 | 100% | 2.00E-138 | 97.98% | 198 | WP_140592929.1 | |
| ref\|WP_120920308.1\| | lipid A 4'-phosphatase [Helicobacter pylori] | Helicobacter pylori | 394 | 394 | 100% | 2.00E-138 | 97.47% | 198 | WP_120920308.1 | |
| gb\|RVY95123.1\| | phosphatase PAP2 family protein [Helicobacter pylori] | Helicobacter pylori | 395 | 395 | 100% | 2.00E-138 | 97.47% | 220 | RVY95123.1 | |
| ref\|WP_001879017.1\| | lipid A 4'-phosphatase [Helicobacter pylori] | Helicobacter pylori | 394 | 394 | 100% | 2.00E-138 | 97.98% | 198 | WP_001879017.1 | |
| gb\|QEF30893.1\| | PAP2 family protein [Helicobacter pylori] | Helicobacter pylori | 395 | 395 | 100% | 2.00E-138 | 97.47% | 220 | QEF30893.1 | |
| ref\|WP_000734127.1\| | lipid A 4'-phosphatase [Helicobacter pylori] | Helicobacter pylori | 394 | 394 | 100% | 2.00E-138 | 97.47% | 198 | WP_000734127.1 | |
| Select seq gb\|NHA94340.1\| | phosphatase PAP2 family protein [Helicobacter pylori] | Helicobacter pylori | 395 | 395 | 100% | 2.00E-138 | 97.47% | 220 | NHA94340.1 | |
| gb\|RVY47576.1\| | phosphatase PAP2 family protein [Helicobacter pylori] | Helicobacter pylori | 395 | 395 | 100% | 2.00E-138 | 97.47% | 220 | RVY47576.1 | |
| ref\|WP_000734376.1\| | lipid A 4'-phosphatase [Helicobacter pylori] | Helicobacter pylori | 394 | 394 | 100% | 2.00E-138 | 97.47% | 198 | WP_000734376.1 | |
| ref\|WP_164499406.1\| | lipid A 4'-phosphatase [Helicobacter pylori] | Helicobacter pylori | 394 | 394 | 100% | 3.00E-138 | 97.98% | 198 | WP_164499406.1 | |
| gb\|MUU42466.1\| | phosphatase PAP2 family protein [Helicobacter pylori] | Helicobacter pylori | 395 | 395 | 100% | 3.00E-138 | 97.47% | 220 | MUU42466.1 | |
| ref\|WP_183001731.1\| | lipid A 4'-phosphatase [Helicobacter pylori] | Helicobacter pylori | 394 | 394 | 100% | 3.00E-138 | 97.47% | 198 | WP_183001731.1 | |
| ref\|WP_181322729.1\| | lipid A 4'-phosphatase [Helicobacter pylori] | Helicobacter pylori | 394 | 394 | 100% | 3.00E-138 | 97.47% | 198 | WP_181322729.1 | |
| ref\|WP_198935015.1\| | phosphatase PAP2 family protein [Helicobacter pylori] | Helicobacter pylori | 394 | 394 | 100% | 3.00E-138 | 97.47% | 198 | WP_198935015.1 | |
| ref\|WP_164500303.1\| | lipid A 4'-phosphatase [Helicobacter pylori] | Helicobacter pylori | 394 | 394 | 100% | 3.00E-138 | 97.47% | 198 | WP_164500303.1 | |
| gb\|KAA6507358.1\| | phosphatase PAP2 family protein [Helicobacter pylori] | Helicobacter pylori | 395 | 395 | 100% | 3.00E-138 | 97.47% | 220 | KAA6507358.1 | |
| ref\|WP_078272055.1\| | lipid A 4'-phosphatase [Helicobacter pylori] | Helicobacter pylori | 394 | 394 | 100% | 3.00E-138 | 97.47% | 198 | WP_078272055.1 | |
| ref\|WP_001899089.1\| | lipid A 4'-phosphatase [Helicobacter pylori] | Helicobacter pylori | 394 | 394 | 100% | 3.00E-138 | 97.47% | 198 | WP_001899089.1 | |
| ref\|WP_001881468.1\| | lipid A 4'-phosphatase [Helicobacter pylori] | Helicobacter pylori | 394 | 394 | 100% | 4.00E-138 | 97.47% | 198 | WP_001881468.1 | |
| gb\|RKV52956.1\| | hypothetical protein DD774_07985 [Helicobacter pylori] | Helicobacter pylori | 394 | 394 | 100% | 4.00E-138 | 97.47% | 220 | RKV52956.1 | |
| ref\|WP_164864154.1\| | lipid A 4'-phosphatase [Helicobacter pylori] | Helicobacter pylori | 394 | 394 | 100% | 4.00E-138 | 97.47% | 198 | WP_164864154.1 | |
| ref\|WP_033743804.1\| | lipid A 4'-phosphatase [Helicobacter pylori] | Helicobacter pylori | 394 | 394 | 100% | 4.00E-138 | 96.97% | 198 | WP_033743804.1 | |
| ref\|WP_000734122.1\| | lipid A 4'-phosphatase [Helicobacter pylori] | Helicobacter pylori | 394 | 394 | 100% | 4.00E-138 | 96.97% | 198 | WP_000734122.1 | |
| ref\|WP_000734131.1\| | lipid A 4'-phosphatase [Helicobacter pylori] | Helicobacter pylori | 394 | 394 | 100% | 4.00E-138 | 97.47% | 198 | WP_000734131.1 | |
| ref\|WP_001925529.1\| | lipid A 4'-phosphatase [Helicobacter pylori] | Helicobacter pylori | 394 | 394 | 100% | 4.00E-138 | 97.47% | 198 | WP_001925529.1 | |
| gb\|RVZ28184.1\| | phosphatase PAP2 family protein [Helicobacter pylori] | Helicobacter pylori | 394 | 394 | 100% | 4.00E-138 | 97.47% | 220 | RVZ28184.1 | |
| gb\|KAA6495834.1\| | phosphatase PAP2 family protein [Helicobacter pylori] | Helicobacter pylori | 394 | 394 | 100% | 4.00E-138 | 97.47% | 220 | KAA6495834.1 | |
| ref\|WP_001930879.1\| | lipid A 4'-phosphatase [Helicobacter pylori] | Helicobacter pylori | 394 | 394 | 100% | 4.00E-138 | 97.47% | 198 | WP_001930879.1 | |
| ref\|WP_001953015.1\| | lipid A 4'-phosphatase [Helicobacter pylori] | Helicobacter pylori | 394 | 394 | 100% | 4.00E-138 | 97.47% | 198 | WP_001953015.1 | |
| ref\|WP_001876362.1\| | lipid A 4'-phosphatase [Helicobacter pylori] | Helicobacter pylori | 394 | 394 | 100% | 4.00E-138 | 97.47% | 198 | WP_001876362.1 | |
| ref\|WP_079360663.1\| | lipid A 4'-phosphatase [Helicobacter pylori] | Helicobacter pylori | 394 | 394 | 100% | 4.00E-138 | 97.47% | 198 | WP_079360663.1 | |
| ref\|WP_120905332.1\| | lipid A 4'-phosphatase [Helicobacter pylori] | Helicobacter pylori | 393 | 393 | 100% | 5.00E-138 | 97.47% | 198 | WP_120905332.1 | |
| ref\|WP_001935451.1\| | lipid A 4'-phosphatase [Helicobacter pylori] | Helicobacter pylori | 393 | 393 | 100% | 5.00E-138 | 97.47% | 198 | WP_001935451.1 | |
| ref\|WP_001929994.1\| | lipid A 4'-phosphatase [Helicobacter pylori] | Helicobacter pylori | 393 | 393 | 100% | 5.00E-138 | 97.47% | 198 | WP_001929994.1 | |
| gb\|NHB35001.1\| | phosphatase PAP2 family protein [Helicobacter pylori] | Helicobacter pylori | 394 | 394 | 100% | 5.00E-138 | 96.97% | 220 | NHB35001.1 | |
| gb\|NHB46588.1\| | phosphatase PAP2 family protein [Helicobacter pylori] | Helicobacter pylori | 394 | 394 | 100% | 6.00E-138 | 96.97% | 220 | NHB46588.1 | |
| gb\|MUU21440.1\| | phosphatase PAP2 family protein [Helicobacter pylori] | Helicobacter pylori | 394 | 394 | 100% | 6.00E-138 | 96.97% | 220 | MUU21440.1 | |
| ref\|WP_165510382.1\| | lipid A 4'-phosphatase [Helicobacter pylori] | Helicobacter pylori | 393 | 393 | 100% | 6.00E-138 | 96.97% | 198 | WP_165510382.1 | |
| ref\|WP_078257001.1\| | lipid A 4'-phosphatase [Helicobacter pylori] | Helicobacter pylori | 393 | 393 | 100% | 7.00E-138 | 97.47% | 198 | WP_078257001.1 | |
| gb\|MUU33109.1\| | phosphatase PAP2 family protein [Helicobacter pylori] | Helicobacter pylori | 394 | 394 | 100% | 7.00E-138 | 96.97% | 220 | MUU33109.1 | |
| ref\|WP_120927257.1\| | lipid A 4'-phosphatase [Helicobacter pylori] | Helicobacter pylori | 393 | 393 | 100% | 7.00E-138 | 97.47% | 198 | WP_120927257.1 | |
| gb\|NHB47888.1\| | phosphatase PAP2 family protein [Helicobacter pylori] | Helicobacter pylori | 394 | 394 | 100% | 7.00E-138 | 96.97% | 220 | NHB47888.1 | |
| gb\|MUU19870.1\| | phosphatase PAP2 family protein [Helicobacter pylori] | Helicobacter pylori | 394 | 394 | 100% | 7.00E-138 | 97.47% | 220 | MUU19870.1 | |
| ref\|WP_198968284.1\| | phosphatase PAP2 family protein [Helicobacter pylori] | Helicobacter pylori | 393 | 393 | 100% | 7.00E-138 | 96.97% | 198 | WP_198968284.1 | |
| gb\|PUD21967.1\| | hypothetical protein C2S44_07380 [Helicobacter pylori] | Helicobacter pylori | 394 | 394 | 100% | 7.00E-138 | 96.97% | 220 | PUD21967.1 | |
| ref\|WP_001905320.1\| | lipid A 4'-phosphatase [Helicobacter pylori] | Helicobacter pylori | 393 | 393 | 100% | 7.00E-138 | 97.47% | 198 | WP_001905320.1 | |
| gb\|MUU55683.1\| | phosphatase PAP2 family protein [Helicobacter pylori] | Helicobacter pylori | 394 | 394 | 100% | 8.00E-138 | 96.97% | 220 | MUU55683.1 | |
| ref\|WP_000734123.1\| | lipid A 4'-phosphatase [Helicobacter pylori] | Helicobacter pylori | 393 | 393 | 100% | 8.00E-138 | 96.97% | 198 | WP_000734123.1 | |
| ref\|WP_165533572.1\| | lipid A 4'-phosphatase [Helicobacter pylori] | Helicobacter pylori | 393 | 393 | 100% | 8.00E-138 | 97.47% | 198 | WP_165533572.1 | |
| gb\|MUU38795.1\| | phosphatase PAP2 family protein [Helicobacter pylori] | Helicobacter pylori | 394 | 394 | 100% | 8.00E-138 | 96.97% | 220 | MUU38795.1 | |
| ref\|WP_121079003.1\| | lipid A 4'-phosphatase [Helicobacter pylori] | Helicobacter pylori | 393 | 393 | 100% | 8.00E-138 | 97.47% | 198 | WP_121079003.1 | |
| ref\|WP_014658559.1\| | lipid A 4'-phosphatase [Helicobacter pylori] | Helicobacter pylori | 393 | 393 | 100% | 8.00E-138 | 97.47% | 198 | WP_014658559.1 | |
| ref\|WP_033620541.1\| | lipid A 4'-phosphatase [Helicobacter pylori] | Helicobacter pylori | 393 | 393 | 100% | 8.00E-138 | 96.97% | 198 | WP_033620541.1 | |
| ref\|WP_165568595.1\| | lipid A 4'-phosphatase [Helicobacter pylori] | Helicobacter pylori | 393 | 393 | 100% | 9.00E-138 | 96.97% | 198 | WP_165568595.1 | |
| ref\|WP_162980605.1\| | lipid A 4'-phosphatase [Helicobacter pylori] | Helicobacter pylori | 393 | 393 | 100% | 9.00E-138 | 96.97% | 198 | WP_162980605.1 | |
| gb\|PUD46994.1\| | hypothetical protein C2R73_04680 [Helicobacter pylori] | Helicobacter pylori | 394 | 394 | 100% | 9.00E-138 | 96.97% | 220 | PUD46994.1 | |
| ref\|WP_097702434.1\| | lipid A 4'-phosphatase [Helicobacter pylori] | Helicobacter pylori | 392 | 392 | 100% | 9.00E-138 | 97.47% | 198 | WP_097702434.1 | |
| gb\|EQD89624.1\| | PAP2 superfamily protein [Helicobacter pylori SouthAfrica50] | Helicobacter pylori SouthAfrica50 | 392 | 392 | 100% | 9.00E-138 | 96.97% | 198 | EQD89624.1 | |
| ref\|WP_001938056.1\| | lipid A 4'-phosphatase [Helicobacter pylori] | Helicobacter pylori | 392 | 392 | 100% | 1.00E-137 | 96.97% | 198 | WP_001938056.1 | |
| gb\|PUD66003.1\| | hypothetical protein C2R76_07790 [Helicobacter pylori] | Helicobacter pylori | 393 | 393 | 100% | 1.00E-137 | 96.97% | 220 | PUD66003.1 | |
| gb\|RVZ06550.1\| | phosphatase PAP2 family protein [Helicobacter pylori] | Helicobacter pylori | 393 | 393 | 100% | 1.00E-137 | 96.97% | 220 | RVZ06550.1 | |
| gb\|PUD63892.1\| | hypothetical protein C2R77_04475 [Helicobacter pylori] | Helicobacter pylori | 393 | 393 | 100% | 1.00E-137 | 96.46% | 220 | PUD63892.1 | |

**Table S8. Virulence factor of the hypothetical protein of LpxF.**

| **Protein** | **Virulence Factors** | | | | |
| --- | --- | --- | --- | --- | --- |
| Lipid A 4'-phosphatase (lpxF) | **VFDB ID** | **Gene symbol** | **Description** | **Score (Bits)** | **E-value** |
|  | VFG002340 | fliR | Flagellar biosynthetic protein | 28 | 0.66 |
|  | VFG011439 | lpxE | Phosphatidyl glycerophosphates | 27 | 1.5 |
|  | VFG010882 | ccmF | Cytochrome c heme lyase subunit | 26 | 1.9 |
|  | VFG045531 | lpg1661 | Dot/Icm Type IV secretion synthase effect | 25 | 4.3 |
|  | VFG000495 | ssaC | Type III secretion system synthetin | 24 | 7.3 |
|  | VFG020191 | yadA | Trimeric autotransporter adhesin | 24 | 9.5 |
|  | VFG002437 | boaB | Autotransporter protein | 24 | 9.5 |

**Table** **S9**. Predicted interface residues involved in hydrogen bonding interaction with the TLR2, TLR4 and TLR5 with Vaccine construct.

| **Complex** | **Interaction type** | **Protein** | | **Vaccine** | | **Bond length (Å)** |
| --- | --- | --- | --- | --- | --- | --- |
|  |  | **Amino Acids** | **Atom** | **Amino Acids** | **Atom** |  |
| Vaccine- TLR2 | Hydrogen bonds | Thr611 | O1 | Lys157 | N | 2.67 |
|  |  | Ser613 | O1 | Lys157 | N | 2.50 |
|  |  | Cys609 | O1 | Lys160 | N | 3.10 |
|  |  | Asn579 | O1 | Lys160 | N | 2.65 |
|  |  | Glu608 | O1 | Lys160 | N | 2.91 |
|  |  | Glu586 | O2 | Lys154 | N | 2.52 |
|  |  | Glu586 | O1 | Lys154 | N1 | 2.59 |
|  |  | Asp580 | O1 | Lys160 | N | 2.84 |
|  |  | His458 | N1 | His134 | O | 2.82 |
|  |  | Asn409 | O | His134 | N1 | 2.81 |
|  |  | Gln578 | N2 | Asn126 | O | 2.94 |
|  |  | Asp550 | O1 | Arg128 | N1 | 2.85 |
|  |  | Asp550 | O1 | Arg128 | N2 | 2.69 |
|  |  | Glu509 | O1 | Asn149 | N | 3.02 |
|  |  | Glu509 | O2 | Asn149 | N2 | 2.97 |
|  |  | Glu509 | O1 | Gln148 | N2 | 2.79 |
|  |  | Lys560 | N1 | Gln148 | O1 | 2.59 |
|  |  | Gln505 | N1 | Thr129 | O | 2.90 |
|  |  | Asn531 | O1 | Ser145 | N | 2.86 |
|  |  | Asn35 | N1 | Asp18 | O | 2.85 |
|  |  | Cys29 | O1 | Arg13 | N1 | 2.72 |
|  |  | Glu27 | O1 | Arg13 | N1 | 2.66 |
|  |  | Glu31 | O2 | Ser21 | N | 2.83 |
|  | Hydrophobic interactions | Ala610  Gln616  Asn433  Arg606  His456  Leu553  His529  Ser552  Ser528  Phe533  Met557  His555  Pro34  Pro28  Tyr38 | | Gly158  Trp125  Thr133  Asn136  Gly135  Phe127  Gln130  Gly147  Phe156  Pro143  Gly144  Gly17  Phe15  Val120 | | |
|  | Salt bridges | Glu608 | O1 | Lys160 | N1 | |
|  |  | Asp580 | O2 | Lys159 | N1 | |
|  |  | His431 | N1 | Asp131 | O2 | |
| Vaccine- TLR4 | Hydrogen bonds | Lys615 | N1 | Glu183 | O | 2.74 |
|  |  | Ala610 | O | Arg179 | N1 | 2.71 |
|  |  | Ala610 | O | Arg179 | N2 | 2.94 |
|  |  | Gln616 | N1 | Asp181 | O2 | 2.89 |
|  |  | Gln616 | O1 | Arg179 | N2 | 2.79 |
|  |  | Asp614 | O | Ala182 | N | 2.99 |
|  |  | Glu605 | O1 | The197 | O1 | 2.89 |
|  |  | Glu135 | O2 | Asn149 | N2 | 2.88 |
|  |  | Asp580 | O1 | Phe15 | N | 2.95 |
|  |  | Leu553 | O | Arg13 | N1 | 2.57 |
|  |  | Asn554 | O | Arg13 | N1 | 2.65 |
|  |  | His529 | N2 | Arg13 | N2 | 2.94 |
|  |  | Arg606 | N | Met11 | O | 3.00 |
|  |  | Asn579 | O | Thr12 | O1 | 2.9 |
|  |  | Asn579 | N | Thr12 | O1 | 2.93 |
|  |  | Glu42 | O | Lys157 | N | 2.58 |
|  |  | Glu42 | O1 | Lys157 | N | 2.61 |
|  |  | Glu42 | O2 | Gly158 | N | 2.93 |
|  |  | Asp181 | O2 | Ser137 | N | 2.98 |
|  |  | Arg87 | N | Lys154 | O | 3.07 |
|  |  | Arg87 | N2 | Gln148 | O | 2.62 |
|  |  | Lys230 | N | His134 | O | 2.54 |
|  |  | Lys230 | N | Gly135 | O | 2.59 |
|  |  | Asp209 | O2 | Asn136 | N | 2.82 |
|  |  | Arg289 | N1 | Thr133 | O | 2.68 |
|  |  | Arg289 | N1 | Thr133 | O1 | 2.72 |
|  |  | Lys130 | N | Leu138 | O | 2.72 |
|  |  | Gln39 | O1 | Gln164 | N | 2.78 |
|  | Hydrophobic interactions | Mer41  Phe263  Leu623  Met618  Pro612  Ser613  Cys609  Gly617  His159  His555  Glu608  Gln578  Leu180  Thr110  Ser86  Phe63  Ala291  Val316  Thr232  Asn156  Glu154  His179  Vval130 | | Ala162  Arg184  Leu188  Lue186  Ile14  Val19  Val126  Gly10  Met161  Ile146  Val155  Gly147  Ser139  Gly163  Phe118 | | |
|  | Salt bridges | Lys615 | N1 | Glu183 | O2 | |
|  |  | Arg23 | N1 | Asp131 | O1 | |
| Vaccine- TLR5 | Hydrogen bonds | Asn510 | O1 | Arg34 | N2 | 2.97 |
|  |  | Gln559 | O1 | Arg34 | N | 2.96 |
|  |  | Asn512 | O1 | Tyr57 | O | 2.78 |
|  |  | Asn512 | N2 | Tyr57 | O | 3.23 |
|  |  | Arg537 | N1 | Gln80 | O1 | 2.87 |
|  | Hydrophobic interactions | Phe643  Cys646  Leu642  His511  Ala373  Lys414  Leu654  Leu650 | | Val645  Leu642  Cys36  Trp238  Thr656  Leu652  Phe653  Thr649  Phe55 | | |
